# Oxylipin-mediated metabolic signatures of symbiosis homeostasis and thermal stress in a model sea anemone

**DOI:** 10.1093/ismejo/wrag143

**Published:** 2026-06-07

**Authors:** Marina T Botana, Robert E Lewis, Kevin Mitchell, Olivier Salamin, Johanna Revol-Cavalier, Evan Heit, Andrea Gamba, Shaun P Wilkinson, Sabrina L Rosset, Clinton A Oakley, Matthew R Nitschke, Mats Hamberg, Arthur R Grossman, David J Suggett, Virginia M Weis, Craig E Wheelock, Simon K Davy

**Affiliations:** School of Biological Sciences, Victoria University of Wellington, Wellington 6012, New Zealand; Unit of Integrative Metabolomics, Institute of Environmental Medicine, Karolinska Institutet, 171 77 Stockholm, Sweden; School of Biological Sciences, Victoria University of Wellington, Wellington 6012, New Zealand; Callaghan Innovation, 69 Gracefield Rd, Lower Hutt 5010, New Zealand; Unit of Integrative Metabolomics, Institute of Environmental Medicine, Karolinska Institutet, 171 77 Stockholm, Sweden; Unit of Integrative Metabolomics, Institute of Environmental Medicine, Karolinska Institutet, 171 77 Stockholm, Sweden; Larodan Research Laboratory, Karolinska Institutet, 171 65 Stockholm, Sweden; School of Biological Sciences, Victoria University of Wellington, Wellington 6012, New Zealand; School of Biological Sciences, Victoria University of Wellington, Wellington 6012, New Zealand; Wilderlab, Miramar, Wellington, New Zealand; Hawaiʻi Institute of Marine Biology, University of Hawaiʻi at Manoa, Kāneʻohe, HI, United States; Department of Biochemistry and Molecular Biology, Michigan State University, East Lansing, MI, United States; School of Biological Sciences, Victoria University of Wellington, Wellington 6012, New Zealand; School of Biological Sciences, Victoria University of Wellington, Wellington 6012, New Zealand; Australian Institute of Marine Science, 1526 Cape Cleveland Road, Cape Cleveland, 4810 QLD, Australia; Unit of Integrative Metabolomics, Institute of Environmental Medicine, Karolinska Institutet, 171 77 Stockholm, Sweden; Larodan Research Laboratory, Karolinska Institutet, 171 65 Stockholm, Sweden; Department of Plant Biology, Carnegie Institution for Science, Stanford, CA 94305, United States; KAUST Coral Restoration Initiative (KCRI) and Division of Biological and Environmental Science and Engineering (BESE), King Abdullah University of Science and Technology, Thuwal 23955, Saudi Arabia; Climate Change Cluster (C3), University of Technology Sydney, Ultimo, NSW 2007, Australia; Department of Integrative Biology, Oregon State University, Corvallis, OR 97331, United States; Unit of Integrative Metabolomics, Institute of Environmental Medicine, Karolinska Institutet, 171 77 Stockholm, Sweden; Department of Respiratory Medicine and Allergy, Karolinska University Hospital, 171 76 Stockholm, Sweden; School of Biological Sciences, Victoria University of Wellington, Wellington 6012, New Zealand

**Keywords:** octadecanoid, lipidomics, Symbiodiniaceae, coral reef, *Exaiptasia diaphana*, coral bleaching, SFC–MS, LC–MS

## Abstract

Oxylipins are oxygenated products of fatty acids proposed to exert a regulatory role in cnidarian–dinoflagellate symbiosis; however, this has not been investigated in detail. We integrated physiological measurements and molecular phenotyping with comparative transcriptome mining to examine how the symbiotic cnidarian model, the sea anemone Aiptasia (i.e. *Exaiptasia diaphana*), and its dinoflagellate symbiont *Breviolum minutum* respond to symbiosis and thermal stress. We performed lipidomics in combination with the quantification of oxylipins, including octadecanoids, eicosanoids, and docosanoids derived from C18, C20, and C22 fatty acids, respectively, and reconstructed their putative biosynthetic routes through cross-phylogenetic protein sequence homology. Relative to aposymbiotic, symbiotic anemones were enriched with omega-3 fatty acids and downstream octadecanoids of symbiont origin, consistent with inter-partner metabolite flux. Cytochrome P450–derived eicosanoids and docosanoids increased up to 300-fold in symbiotic versus aposymbiotic anemones. Under elevated temperature, anemones showed minor changes in their physiology and lipid profiles; however, the symbiont fraction displayed multiple signatures of stress. In comparison, aposymbiotic anemones showed a 50% reduction in protein abundance as well as structural and storage lipids, while simultaneously accumulating oxylipins linked to inflammation and oxidative stress. Our findings report novel oxylipins that have not been previously observed in dinoflagellates. We identified regulatory pathways that are conserved across cnidarians and higher metazoans, advancing our understanding of cnidarian–dinoflagellate symbiosis and its response to warming climate. We are targeting specific oxylipins and signalling pathways for further research that may aid molecular intervention strategies for selective breeding and assisted evolution to enhance coral resilience in warming oceans.

## Introduction

Regulation of inter-partner homeostasis underpins the maintenance of the cnidarian–dinoflagellate symbiosis, which is vital for the ecological success of coral reefs. Previous research has suggested that homeostasis and thermal resilience are shaped, in part, by inter-partner immunoregulation between the dinoflagellate symbiont and its cnidarian host [1, 2], although the lipid-mediated signalling pathways that facilitate this regulation are largely unknown [3]. Warming seawater temperatures are linked to coral reef decline worldwide via a process known as coral bleaching, whereby the host coral loses its endosymbiotic dinoflagellates (family: Symbiodiniaceae), leading to severe risk of coral mortality [4, 5]. Bleaching events have increased in frequency and intensity, and while the improvement of monitoring tools has helped understand their occurrence and dynamics [6, 7], the molecular and cellular mechanisms involved in symbiosis homeostasis and dysfunction remain uncertain (reviewed in [4]).

Oxylipins are a broad family of oxygenated products of fatty acids, many of which have regulatory functions across biological systems, including roles in cell development, immunoregulation, and host/pathogen interactions [8, 9]. The synthesis of oxylipins is also influenced by environmental factors [10–12], and increased temperatures can lead to greater autoxidation of polyunsaturated fatty acids (PUFAs) in lipid membranes by reactive species (e.g. reactive oxygen and nitrogen species) [12–14]. Indeed, the ‘oxidative stress theory’ is widely discussed as the major cause of bleaching, in which warmer seawater temperatures are proposed to lead to higher rates of lipid peroxidation and consequent impairment of energy-transducing membranes, both in the host and symbionts [4, 15, 16]. It is still unknown whether dysbiosis is regulated by transcription networks involving oxylipins and other signalling metabolites, or whether it is caused by homeostatic breakdown and coral starvation [17], or, most likely, by a combination of both.

A myriad of C18, C20, and C22 fatty acids act as substrates for enzymatic and chemical oxylipin synthesis in the coral holobiont—the combination of the cnidarian host, algal symbionts, and other associated microbes—suggesting the presence of a highly diverse and complex array of related pathways [18–22]. Oxylipins from the lipoxygenase (LOX), cyclooxygenase (COX), and cytochrome P450 family (CYP) enzymatic pathways are well-known immunomodulators of inflammatory responses and apoptosis cascades in humans and other mammals [23]. The conservation of innate immunity pathways between cnidarians and more complex metazoans [24, 25] suggests that oxylipins play a role in the cnidarian–dinoflagellate symbiosis. For instance, eicosanoids from the COX pathway (e.g. prostaglandins) have been extensively described in corals [22, 26–28], but never functionally explored as signalling mediators. In the case of Symbiodiniaceae, a wide variety of octadecanoids with synthesis pathways linked to 9*S*- and 13*S*-LOXes, and CYP74 (analogous to the CYP family in higher metazoans) family of enzymes were reported in our previous study [19], but other potential oxylipins derived from C20 and C22 fatty acids and their synthesis pathways remain uncharacterized. To explore the role of oxylipins as lipid mediators of the cnidarian–dinoflagellate symbiosis, including under thermal stress, we used the model sea anemone *Exaiptasia diaphana* (commonly referred to as ‘Aiptasia’) when in symbiosis with its native symbiont *Breviolum minutum*. Symbiont-free (i.e. ‘aposymbiotic’) anemones were also analysed to determine the effects of symbiotic state and thermal stress in the host. For *Branchioglossum minutum*, only the effects of thermal stress were evaluated here, though a previous, unpublished dataset from cultured cells grown under different nutrient conditions was used to assess species-specific contributions to the biosynthesis of oxylipins. We measured lipids, photosynthetic pigments, and oxylipins (octadecanoids, eicosanoids, and docosanoids) and performed a physiological assessment of both host and symbiont. The acquired lipid and oxylipin profiles were used as guidance to perform comparative transcriptome mining to evaluate the expression of biosynthetic enzymes and construct oxylipin metabolic pathways.

## Materials and methods

### Experimental organisms

#### Aiptasia animal stocks

Long-term clonal stocks of the sea anemone *Exaiptasia diaphana* (Aiptasia) (culture ID: NZ1) of average pedal disc diameter 3 mm were rendered aposymbiotic using a menthol protocol [29]. They were confirmed to be symbiont-free by the absence of chlorophyll-*a* autofluorescence under confocal microscopy (Olympus Provis AX70; 100× magnification) and the absence of symbiont DNA amplification through polymerase chain reaction using ITS2_F and ITS_R primers from holobiont DNA extracts [30]. Half of the aposymbiotic anemones (~200 individuals) were inoculated with the homologous symbiont *Breviolum minutum* (ITS2 type B1; culture ID: CCMP830) and maintained in symbiosis for 6 months. All anemones were maintained in filtered seawater (0.22 μm) and fed once a week with freshly hatched *Artemia* sp. nauplii. All anemones were kept at 25°C and 90–110 μmol photons m^−2^.s^−1^ on a 12:12 h light/dark cycle using Philips 6500K bulbs.

#### *Breviolum minutum* cultures

*Breviolum minutum* cultures were grown for 8 years in f/2-enriched 0.22 μm filtered seawater [31]. Culture flasks were maintained inside an incubator set to 25°C (± 0.5°C), under cool fluorescent lights (Osram Dulux 36/W890) at 90–110 μmol photons m^−2^.s^−1^ on a 12:12 h light/dark cycle.

### Experimental set-up and sampling

Aposymbiotic and symbiotic anemones were distributed across eight 400-ml jars (*N* = 8 anemones per jar per symbiotic state) that were subsequently divided into two groups (*N* = 4 jars per group per symbiotic state). One group was set as the ‘Control’ (C), with the water bath temperature maintained consistently at 25°C. The other was set as the ‘Treatment’ (T), with temperature of the water bath ramping up by 0.5°C day^−1^ until reaching the peak temperature of 33.5°C, which was maintained for 36 h. Light conditions were maintained as described above for all treatment groups. On the day of sampling (i.e. at the end of the thermal ramp treatment), jars were dark acclimated for 15 min within 4 h of the start of the light-cycle, and the photophysiological status of the anemone algal endosymbionts assessed as the maximum quantum yield of photosystem II (*F*_v_/*F*_m_) using a Pulse Amplitude Modulated Fluorometer (Diving-PAM, Walz, Effeltrich, Germany; settings: measuring light = 4, saturation intensity = 8, saturation width = 0.8 s, gain = 3, and damping = 3). Subsequently, the eight anemones in each jar were pooled and washed with ice-cold 10 mM sodium phosphate buffer (pH 7.4) containing 100 μM deferoxamine mesylate and flash-frozen with liquid nitrogen to quench metabolism. For sample processing, anemones were homogenized in 1 ml ice-cold buffer using a glass tissue-grinder. Aposymbiotic anemone homogenates were flash-frozen, freeze-dried using a Heto lyophilizer (CT 60), and stored at −80°C in 1.5-ml sterile Eppendorf tubes. Symbiotic anemone homogenates were transferred to sterile Eppendorf tubes and centrifuged at 500×*g* for 5 min at 4°C to separate host and symbiont fractions. Host tissue supernatant was transferred to a separate sterile tube and the pelleted symbiont dinoflagellate fraction was washed by resuspension and centrifugation using the same buffer solution to eliminate host material, and the residue discarded. A 20-μl aliquot of host supernatant was then analysed for protein content using a fluorometric Qubit Protein Assay Kit. After resuspension a second time with 1 ml ice-cold buffer, 100 μl of the symbiont fraction were aliquoted and diluted 20-fold for cell counts (*N* = 10 counts per sample) which were performed using a confocal imaging system IN Cell analyzer 6500HS. Cell numbers were normalized to host protein content to estimate symbiont population density. Another 100-μl aliquot of the symbiont fraction were used to verify the symbiont species present through amplification and sequencing of Symbiodiniaceae internal transcriber sequence 2 (ITS2) using ITS2_F and ITS_R primers (species confirmation on Table S1) [30]. All steps were performed on ice and final fractions of host and symbiont were flash-frozen, freeze-dried, and stored at −80°C until further analysis. Aiptasia host fractions were evaluated for the effects of symbiotic state and thermal stress treatment. For *in hospite B. minutum*, only the effect of thermal stress was evaluated from this experimental dataset.

A posterior analysis of lipids and oxylipins in cultured *B. minutum* was added to our results and evaluated to assess species-specific lipid and oxylipin signatures. Symbionts were grown under different nutrient concentrations (i.e. f/2, f/4, and f/400) (*N* = 5 per condition) in 500-ml Erlenmeyer flasks for 5 months and refreshed monthly to allow acclimation. For the experiment, initial cell densities in flasks were 1000 cells/ml and culture growth was monitored using the confocal imaging system IN Cell analyzer 6500HS, where cell density was quantified with ImageJ processing software (10 counts per replicate). Cultures were monitored over time and biomass sampled after 15 days using sterile pipettes (250-ml per replicate). Samples were centrifuged at 4000×*g* for 5 min and the resulting algal pellets flash-frozen and freeze-dried before storage at −80°C in sterile 1.5-ml Eppendorf tubes.

### Lipidomics and photosynthetic pigment analyses

Lipids and pigments were extracted from freeze-dried biomass according to [32], with modifications (see Supplemental Methods). Dried lipid extracts were resuspended and analysed by Liquid chromatography-high resolution mass spectrometry (LC–HRMS) on a Waters Acquity H-Class UPLC employing a Waters Premier CSH C18 column (1.7 μm, 2.1 mm i.d. × 150 mm) column interfaced with a Waters Xevo G2-XS-QTOF Quadrupole Mass Spectrometer. Data for lipid molecular species identification and semi-quantification were obtained in SONAR-MSE Data Independent Acquisition (DIA) mode. SONAR quadrupole and MSE acquisition settings were ionization mode-specific (see Supplemental Methods). Lipid molecular species were identified using an in-house library. Waters MassLynx software version 4.2 was used for data acquisition and Waters UNIFI for data processing. The platform allowed for the screening of 107 analytes tested for sensitivity, linearity, and replicability with coefficients of variation ≤30%. The calculated semi-quantitative lipid and pigment data were normalized using dry biomass and concentrations reported in micrograms per milligram. All raw mass spectrometry data have been deposited to the MassIVE repository and are publicly accessible via the dataset identifier MSV000101404.

### Octadecanoid analysis

Octadecanoid extraction and quantification were performed according to [33], with modifications. Chiral separation was performed using a Waters Trefoil AMY1 column (3.0 × 150 mm, 2.5 μm) on a supercritical fluid chromatography (SFC) UPC^2^ system (Waters Corp, Milford, USA) coupled to a Xevo TQ-XS mass spectrometer (Waters Corp, Milford, USA). The MS source was operated in negative ionization mode. Quantification of octadecanoids was performed using isotopically labelled internal standards and final calculated data were normalized using dry biomass and concentrations reported in nanograms per gram. MassLynx software version 4.2 was used for data acquisition and TargetLynx for data processing. The method screened 157 octadecanoids (Table S2).

### Eicosanoid and docosanoid analyses

Eicosanoid and docosanoid quantifications were performed according to [34] with some modifications. Following SFC-MS/MS analysis, 10 μl of high performance liquid chromatography (HPLC)-grade water was added to sample vials. Reversed-phase separation was performed using a liquid tandem mass spectrometry (LC–MS/MS) ACQUITY Premier BEH C18 column (2.1 mm × 100 mm, 1.7 μm, Waters, Milford, MA) on a Waters Acquity H-Class UPLC Acquity coupled to a Xevo TQ-XS mass spectrometer (Waters Corp, Milford, USA). Quantification was performed using isotopically labelled internal standards and final calculated data were normalized using dry biomass and concentrations reported in nanograms per gram. MassLynx software version 4.2 was used for data acquisition and TargetLynx for data processing. The method screened 75 eicosanoids and 12 docosanoids (Table S2).

### *De novo* transcriptome assembly and mRNA abundance

To describe the transcriptomic responses of *B. minutum*, but also of other species of Symbiodiniaceae and Anthozoa for expanded evaluation, *de novo* alternatively spliced mRNA transcriptomes were assembled from previously sequenced mRNA-enriched RNAseq libraries (Table S3) with a bioinformatic pipeline as we previously published [19] (see Supplemental Methods).

### Identification of putative biosynthesis candidates and phylogeny assessment

To identify lipoxygenases (LOXes), allene-oxidase-synthase (AOS)-LOX, cyclooxygenases (COX), and prostaglandin-E synthases (PTGS-E) enzyme sequences in the protein-coding transcriptomes of Aiptasia and *B. minutum*, as well as in other species of Anthozoa and Symbiodiniaceae for expanded evaluation, a collection of proteomes of 14 species of different kingdoms in the tree-of-life was curated (including human *Homo sapiens*, rabbit *Oryctolagus cuniculus*, tunicate *Ciona intestinalis*, green algae *Monoraphidium neglectum* and *Auxenochlorella protothecoides*, red alga *Galdieria sulphuraria*, diatom *Skeletonema marinoi*, fungi *Fusarium oxysporum* and *Aspergillus fumigatus*, land plants *Arabidopsis thaliana, Physcomitrium patens*, and *Glycine max*, gorgonian *Paramuricea clavate*, and anemone *Exaiptasia diaphana*) (Table S4). All proteomes (including hypothetical novel sequences) were scanned for homology using OrthoFinder with default settings and with a cutoff of e-value = 1e^−5^ [35]. Presence of Pfam domains within the collection of candidates was confirmed with InterProScan (interproscan.sh v5.68-101.0) [36]. Protein sequence phylogeny of LOXes, AOS-LOXes, COX, and PTGS-E candidates for Anthozoa and Symbiodiniaceae were constructed from MUSCLE multiple sequence alignments, together with the other tree-of-life protein sequence references [37]. The resulting distance matrix provided information to generate the phylogenetic trees using the neighbour-joining clustering method, with branch lengths optimized via phylogenetic maximum-likelihood criterion [38], with Le and Gascuel model parameters [39]. The fitted trees were then bootstrapped 100 times. Characteristic motifs for LOXes, AOS-LOXes, COX, and PTGS-E were further evaluated by amino acid sequence alignment against experimentally validated trustable reference sequences for additional quality control (see Figs. S1–S5 and Table  S5 for details).

### Statistical analyses and data reproducibility

Before evaluating the alterations caused by symbiotic state and thermal treatment, we characterized the putative biosynthesis pathways of oxylipins in both Aiptasia and *B. minutum*, by combining lipid and oxylipin datasets with transcriptome mining. Because biomass was split in two for (1) lipids and photosynthetic pigments and (2) oxylipins, the datasets were individually log_10_ transformed and *z*-score standardized and then combined for further analysis. For the host fraction, permutational multivariate analysis of variance (PERMANOVA) was applied to quantitatively evaluate symbiotic state and thermal stress effects. Differences in abundances of individual lipids were tested with Tukey’s HSD test and the significance threshold set at *P* <.05 with Benjamini–Hochberg false discovery rate (FDR) corrections. Volcano plots were used to visualize differences in lipid and oxylipin abundances in pairwise comparisons between experimental groups using a significance threshold of >2 fold-change and *P* < .05 with the same FDR correction. For the symbiont, only the effect of thermal stress was evaluated, using the same Volcano plot pairwise parameters described above. MetaboAnalyst version 6.0 [40] and GraphPad software were used for statistical analyses and plots. Octadecanoid stereochemistry is reported as the enantiomeric excess (ee) and calculated as the following for each chiral centre:

%ee of (R) or (S) enantiomer = (% of major enantiomer) − (% of minor enantiomer).

## Results

### Biosynthesis pathways of oxylipins in Anthozoa and Symbiodiniaceae

**Figure 1:**
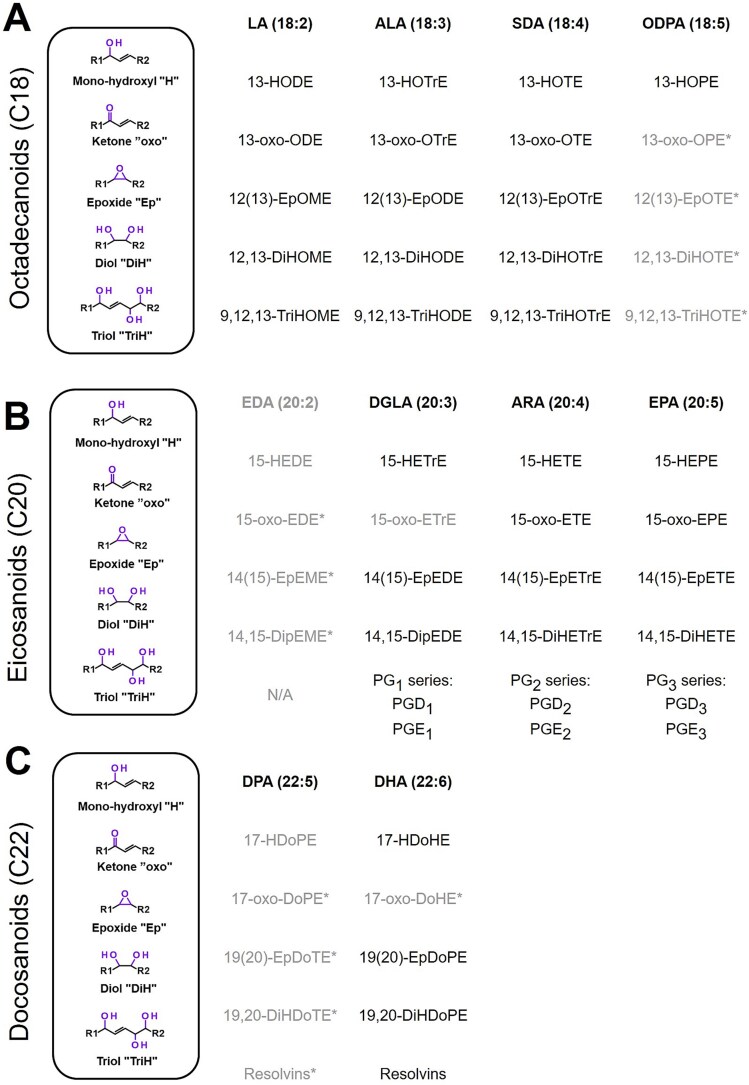
Abbreviated nomenclature system for oxylipins. (A) Octadecanoids, (B) eicosanoids, and (C) docosanoids. Compound names start with the positioning of the oxygenated moiety on the carbon backbone. The number(s) is followed by the abbreviation of the moiety (‘H’ for mono-hydroxyl, ‘oxo’ for ketone, ‘Ep’ for epoxide, ‘Di’ for diols, and ‘TriH’ for triols). Compounds represented in grey were not present in the analytical platforms used for oxylipin measurement in our study and N/a indicates compounds that have not been previously reported. N/a = not applicable.

**Figure 2:**
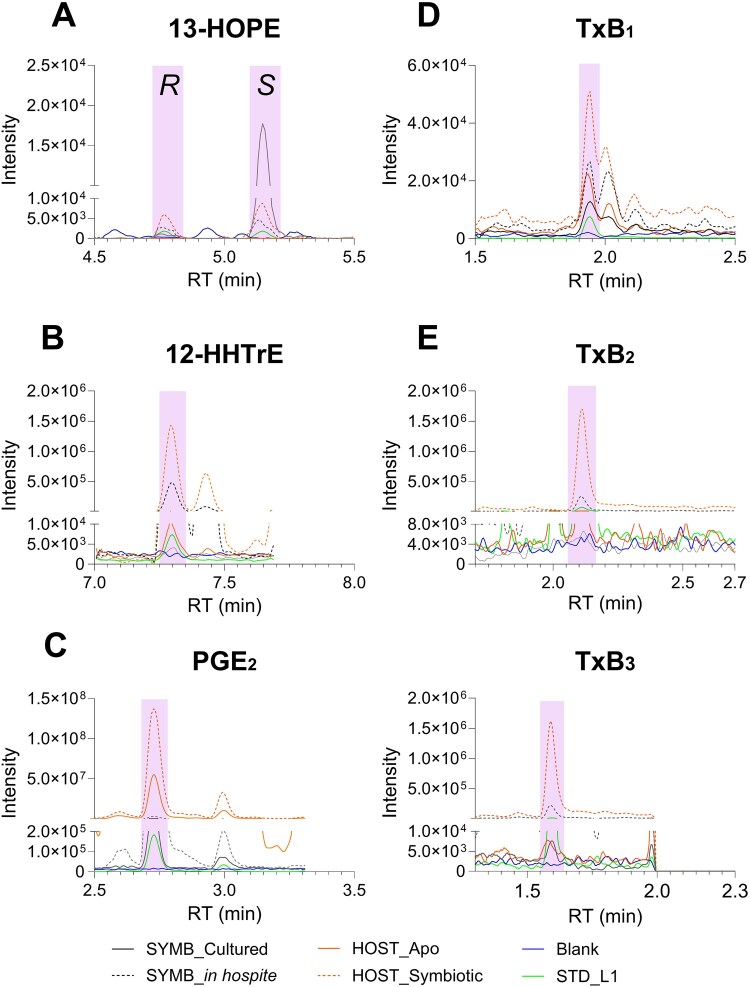
Representative chromatograms of selected oxylipins and their respective MRM transition windows. (A) Hydroxy-octadecapentaenoic acid (13-HOPE) enantiomers from ODPA (18:5, *n*−3); (B) 12-hydroxyheptadecatrienoic acid (12-HHTrE) from ARA (20:4, *n*−6); (C) prostaglandin E_2_ (PGE_2_) from ARA; (D) thromboxane B_1_ (TxB_1_) from DGLA (20:3, *n*−3); (E) thromboxane B_2_ (TxB_2_) from ARA; (F) thromboxane B_3_ (TxB_3_) from EPA (20:5, *n*−3). Peak identification and subsequent quantification were performed with their respective analytical standards. SYMB_cultured (brown solid line) indicates the cultured symbionts. SYMB_in hospite (brown dashed line) indicates the symbionts in symbiosis. HOST_Apo (orange solid line) indicates aposymbiotic Aiptasia. HOST_symbiotic (orange dashed line) indicates symbiotic Aiptasia. STD_L1 (green solid line) indicates the lowest level of the calibration curve (standard curve level 1). Blank (blue solid line) indicates an extraction blank for the analyses.

**Figure 3:**
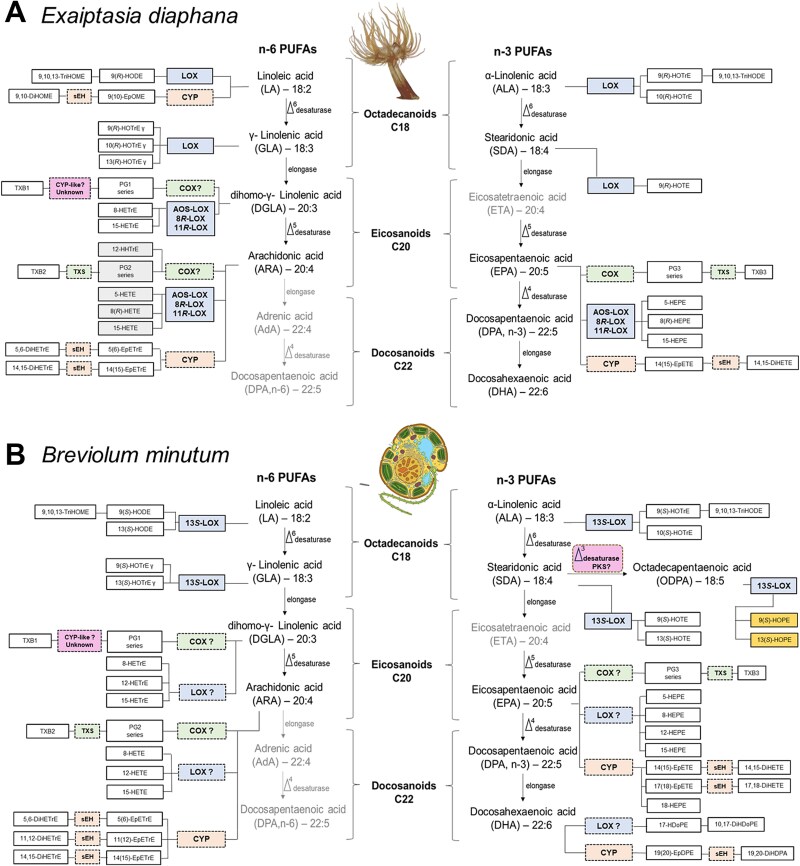
Conceptual model of biosynthesis pathways of fatty acids and oxylipins in (A) Aiptasia and (B) *Breviolum minutum.* Synthesis pathways were modelled based on transcriptome mining and the combination of lipids and oxylipin datasets. Polyunsaturated fatty acid (PUFA) synthesis is shown in the vertical axis and oxylipin biosynthesis is shown in the horizontal axis. Short-lived intermediate compounds were not represented (e.g. TXA_2_). Oxylipin stereochemistry is only indicated for octadecanoids. For those compounds in which the enantiomeric excess (ee) = 100%, enzymatic biosynthesis was inferred. Oxylipins in grey boxes have been reported in previous studies and all others were reported herein for the first time in this system. Oxylipins in yellow were characterized here for the first time in nature. PUFAs in grey were not detected in our dataset. ODPA is exclusively present in the symbiont. Enzymes presented in dashed boxes are proposed to exist, but require experimental validation. Abbreviations: LOX: lipoxygenases (blue boxes); COX: cyclooxygenases (green boxes); CYP: ‘Cytochrome P450-like’ family enzymes (red boxes); TXS: thromboxane synthase; sEH: soluble epoxide hydrolase; PKS: ‘Polyketide synthase’. Symbiont image was obtained from [100]. Oxylipin nomenclature is described in Fig. 1.

A general overview of oxylipin nomenclature is provided in Fig. 1. Compounds are named according to IUPAC rules depending upon their carbon chain length and the associated functional groups. A complete list of all oxylipins including their full and abbreviated names is provided in Table S5. Stereoselective formation of mono-hydroxy oxylipins (i.e. with one –OH group) is indicated by the enantiomeric excess (ee), which refers to the relative formation of one enantiomer versus the other (i.e. *S* vs. *R*). The % ee can be used to infer the route of formation of oxylipins because some enzymes are enantioselective (e.g. 8*R*-LOX) versus autoxidation, which produces a racemic mixture (50% *S* and 50% *R*; % ee = 0). Accordingly, oxylipins that evidence a high % ee value for a specific enantiomer can be inferred to be formed via enzymatic activity. Aposymbiotic anemones and cultured *B. minutum* were first examined to assess species-specific contributions to the putative biosynthesis of fatty acids and oxylipins. All measured PUFAs were detected in both Aiptasia and *B. minutum*, apart from octadecapentaenoic acid (ODPA, 18:5, *n*−3), which was exclusively present in the symbiont (Fig. 2; Tables S6). Oxylipin profiles in both Aiptasia and the dinoflagellate symbiont were highly diverse, encompassing octadecanoids, eicosanoids, and docosanoids putatively derived from the activity of LOX and diverse CYP family enzymes (Fig. 2). By contrast, prostaglandins and thromboxanes, products of COX, and thromboxane synthase (TXS), respectively, were mainly enriched in Aiptasia, except for the prostaglandins E_2_ and E_3_ (PGE_2_ and PGE_3_) and the thromboxane B_1_ (TXB_1_), which were detected in the cell cultures of *B. minutum* and when *in hospite* (Figs. 2–3; Table S6). Notably, the sole Aiptasia sequence previously annotated as COX (XP_020908730.1) was reclassified as a dioxygenase (Fig. S1–S2).

**Figure 4:**
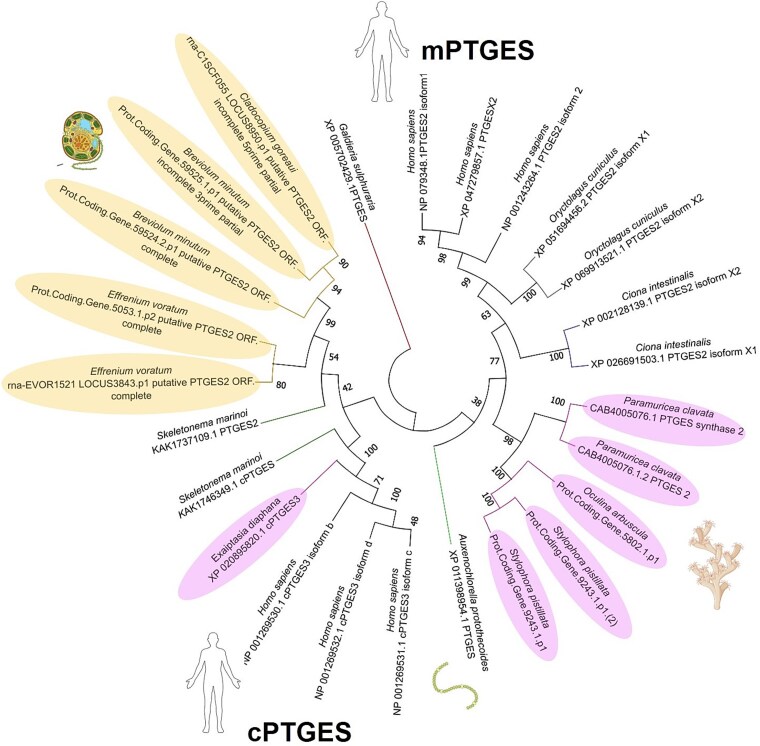
Putative prostaglandin E-synthase (PTGES) enzymes in cnidarians and Symbiodiniaceae. Phylogenetic tree of novel PTGES enzymes based on protein sequence homology. Sequence alignment and expression levels are presented in Fig. S2 and Table  S3. Branch length represents predicted evolutionary distance between enzymes and numbers at nodes represent bootstrap support values, indicating the reliability of each branch separation (see Methods section). Abbreviations: cPTGES: cytosolic PTGES; mPTGES: microsomal PTGES.

We expanded the transcriptomic mining of biosynthesis pathways to also include multiple species of Anthozoa and Symbiodiniaceae for a more comprehensive phylogenetic assessment. We identified enzyme sequences for LOXes (5-LOX, 8*R*-LOX, and 11*R*-LOX), 4 allene-oxide-synthase-LOX fusion proteins (AOS-LOX), and COX in different cnidarian species (Fig. 2; Table S7) (i.e. *Capnella imbricata, Gersemia fruticosa, Dendronephthya gigantea, Desmophyllum pertusum, Oculina arbuscula*, and *Stylophora pistillata*) consistent with previous reports [21, 26, 28]. In addition, we identified novel putative protein sequences for prostaglandin-E synthases (PTGES), which showed active constitutive expression across all tested RNAseq datasets and conserved motif homology with other well-characterized PTGES, including the *H. sapiens* isoforms (Fig. S3). Intriguingly, neither *B. minutum* nor the other Symbiodiniaceae species studied here possessed COX candidates, though we found actively expressed putative PTGES with high sequence homology to other cytosolic PTGES (cPTGES), whereas for cnidarians, all candidates were instead related to microsomal PTGES (mPTGES) (Fig. 4). Sequence alignments were performed for all reported enzyme candidates, including the sequences presented as ‘Protein-coding genes’, in order to verify the presence of conserved diagnostic motifs and confirm their annotation (Figs. S1, S3, and  S4). Phylogenetic trees for LOX and COX contained all enzyme candidates that were actively expressed (>1 transcript per million) (Fig. S2 and S5;  Tables  S3, S4, and S7).

### Influence of symbiosis in Aiptasia

**Figure 5:**
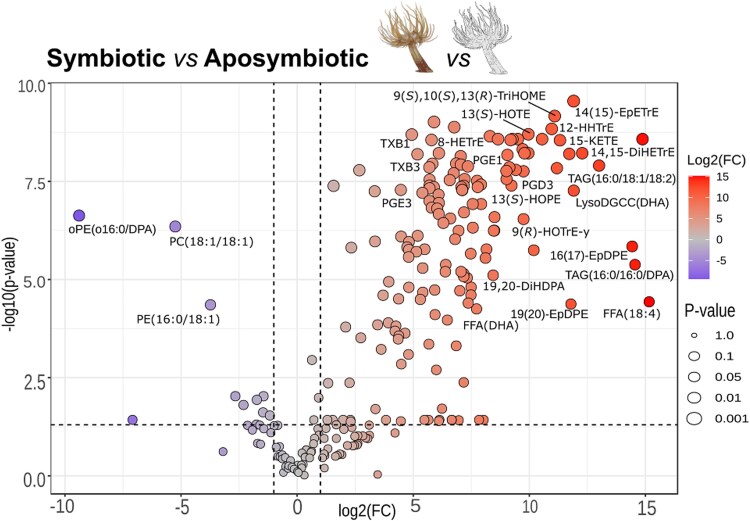
The effects of symbiotic state on the lipids and oxylipin profiles (octadecanoids, eicosanoids, and docosanoids) of Aiptasia colonized with *Breviolum minutum*. Volcano plot displaying significantly different oxylipins in pairwise comparisons: symbiotic (*N* = 4) vs. aposymbiotic (*N* = 4) (FC > 2; *P* < 0.05 with FDR). Upregulated and downregulated compounds are shown in red and blue, respectively. Out of 332 monitored compounds, 152 significantly increased and 11 significantly decreased with symbiosis effect. Dot size and position on the graph refer to *P*-value significance and intensity of variation (see Table S8 for detailed list of compounds and statistics). FC = fold change.

Symbiosis was marked by an increased abundance of storage lipids in the form of triacylglycerol (TAG) (*P* < .05 with FDR) and an up to 4-fold increase of free fatty acids, mainly linked to omega-3 acyl chains, such as stearidonic acid (SDA, 18:4, *n*−3) and docosahexaenoic acid (DHA, 22:6, *n*−3) in symbiotic versus aposymbiotic Aiptasia (Fig. 5; Table S8). Betaine lipids, here represented by DGCCs, were absent in the tissue of the anemone, except for Lyso DGCC-DHA, which only occurred in the symbiotic cohort (Fig. 5; Tables S8).

Symbiosis significantly changed the Aiptasia oxylipin profile (symbiotic vs. aposymbiotic: PERMANOVA *R*^2^ = 0.98, *P* = .003; Fig. 5; Table S8), demonstrated by the presence of 13(*S*)-enantiomers of hydroxy-octadecatetraenoic acid (HOTE) and hydroxy-octadecapentaenoic acid (HOPE) from SDA and ODPA (Fig. 5), which were absent in aposymbiotic anemones. The same pattern was observed for other (*S*) enantiomers of mono-hydroxy octadecanoids (see Fig. 1 for structure nomenclature), and the 9(*S*),10(*S*),13(*R*)-trihydroxy-octadecenoic acid (9(*S*),10(*S*),13(*R*)-TriHOMEs) had ee = 90%, for the 13(*R*) position (Fig. 5; Table S9). Symbiotic anemones also had an up to 300-fold increase of epoxides and their respective vicinal diols, indicative of CYP-dependent epoxidation and subsequent soluble epoxide hydrolase (sEH) conversion of eicosapentaenoic acid (EPA, 20:5, *n*−3), arachidonic acid (ARA, 20:4, *n*−6) and DHA. The greatest increase was for the pair 14(15)-epoxy-eicosatrienoic acid (14(15)-EpETrE) and 14,15-dihydroxy-eicosatrienoic acid (14,15-DiHETrE), also 19(20)-epoxy-docosapentaenoic acid (19(20)-EpDPE) and 19,20-dihydroxy-docosapentaenoic acid (19,20-DiHDoPE) (Fig. 5; Table S8). Upregulated dihomo-γ-linolenic acid (DGLA, 20:3, *n*−6) metabolism was observed for symbiotic anemones with an up to 200-fold increase in 8-hydroxy-eicosatrienoic acid (8-HETrE) (Fig. 5; Table S8). Chiral determination was only performed for the octadecanoids, and the stereochemistry of the 8-HETrE was therefore not determined; however, it is most likely formed from AOS-8*R-*LOX activity [26]. Thromboxane 2 and 3 series (i.e. TXB_2_ and TXB_3_, respectively) and 12-hydroxyheptadecatrienoic acid (12-HHTrE), all derived from thromboxane synthase (TXS) [41], also had an up to 200-fold increase (Fig. 5; Table S8). All COX-derived prostaglandins, including PG_1_ series (i.e. PGE_1_ and PGD_1_) from DGLA, PG_2_ series from ARA (i.e. PGD_2_), and PG_3_ series from EPA (i.e. PGD_3_), were enriched by up to 45-fold (Fig. 5; Table S8).

### Impacts of thermal stress treatment in Aiptasia

**Figure 6:**
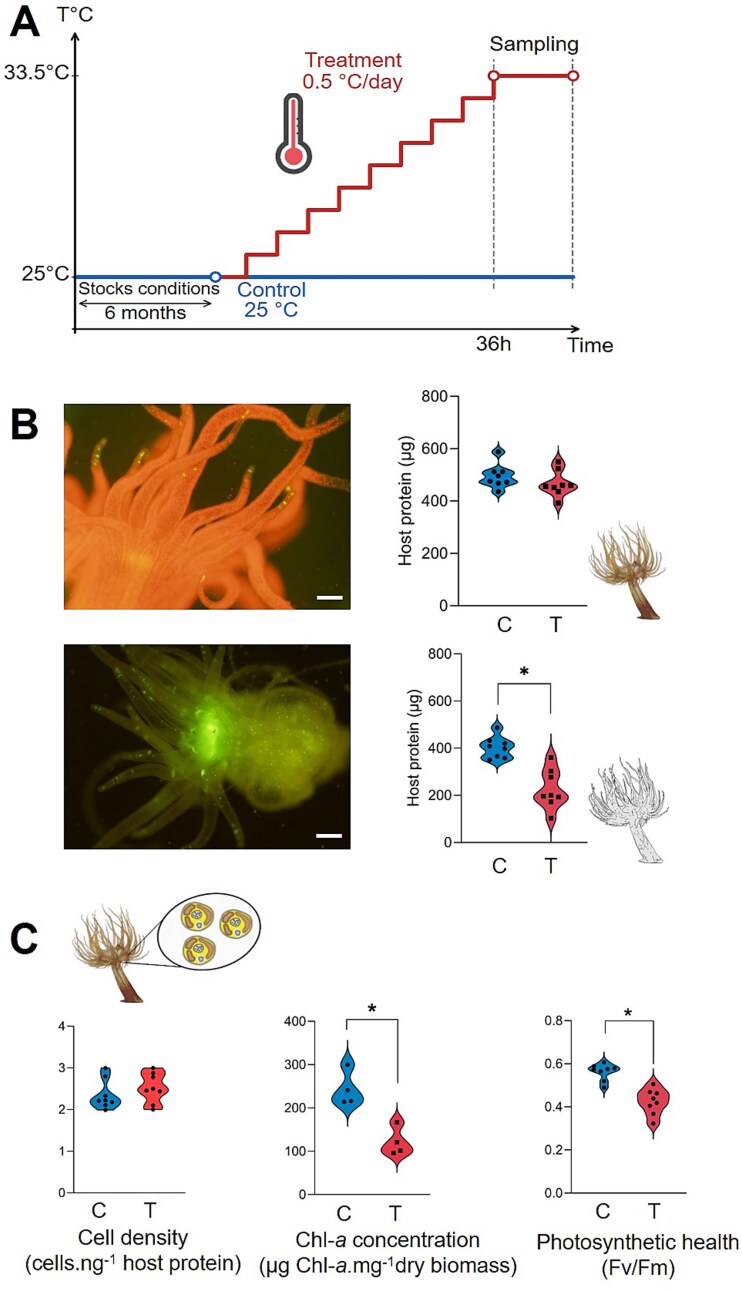
Onset of thermal stress in the physiology of Aiptasia and *Breviolum minutum*. (A) Thermal stress treatment experiment and physiological assessment of (B) Aiptasia and (C) *Breviolum minutum*. Fluorescence microscopy imaging of symbiotic and aposymbiotic anemones at 5× magnification. Scale bars represent 100 μm and autofluorescence of chlorophyll-*a* in symbiont cells is represented in red in symbiotic anemones only. *F*_v_/*F*_m_ (the maximum quantum yield of photosystem II) was used as a metric for symbiont photophysiological health. Blue bars represent organisms at control temperature (C; 25°C) and red bars represent organisms at treatment temperature (T). Values are mean ± SE (*n* = 4; each biological replicate consisted of eight pooled anemones). *P < 0.05 with FDR (see Tables S8 and S10 for detailed statistics).

**Figure 7:**
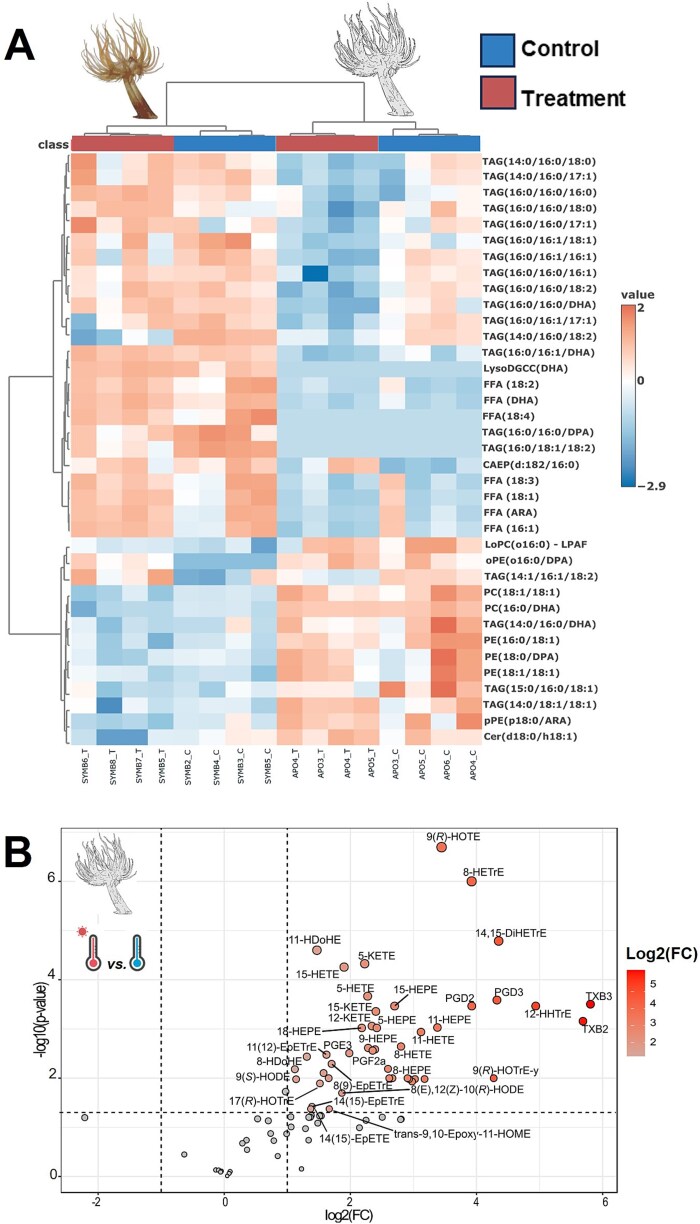
The effect of thermal stress on Aiptasia. (A) Alterations in the lipidome of aposymbiotic and symbiotic Aiptasia. A total of 37 lipids were significantly different among comparison groups. Each coloured cell in the heatmap corresponds to a normalized concentration. Statistical significance was determined via Tukey’s HSD; *P* < 0.05 with FDR (see Table S9). Individual concentrations of lipids are provided in Table S6; (B) the effect of thermal stress on the oxylipin profiles (octadecanoids, eicosanoids, and docosanoids) of aposymbiotic anemones*.* The volcano plot shows 45 significantly different compounds between treatment (elevated temperature; *N* = 4) vs. control (25°C; *N* = 4) (FC > 2; *P* < 0.05 with FDR) (see Table S8 for detailed list). Upregulated and downregulated compounds are shown in red and blue, respectively. Nonsignificant compounds are shown in grey.

Symbiotic anemones did not exhibit a decrease in protein content or symbiont density in response to the thermal stress treatment (increase of 0.5°C.day^−1^ to 33.5°C) (‘Treatment’ in Fig. 6A); however, the photophysiological ‘health’ (maximum quantum yield of Photosystem II, *F*_v_/*F*_m_) of the symbiont population declined (Fig. 6C). We also observed increased concentrations of individual molecular species of TAG (Fig. 7A), but no significant alterations in the oxylipin profiles of symbiotic anemones in response to treatment. In sharp contrast, aposymbiotic anemones experienced a 60% drop in their protein content (Fig. 6B), and a significant decrease in the abundance of their TAG storage lipids and free fatty acids (*P* < .05 with FDR, Fig. 7A) in response to treatment. At the membrane level, there was an increase in mono-unsaturated and saturated fatty acids esterified to the acyl chains of polar lipids (e.g. phosphatidyl-choline and -ethanolamine) (Fig. 7A; Table S10). Aposymbiotic anemones also displayed an up to 10-fold increase of (*R*) enantiomers of mono-hydroxy octadecanoids from GLA and SDA, with ee values ranging from 64% to 100%, together with higher autoxidation levels characterized by an 8-fold increase in EpODAs, EpODEs, and epoxy-HOMEs (ee < 15%) (Fig. 7B; Tables S9). The abundance of PGD_2_, PGD_3_, TXB_2_, and TXB_3_ increased up to 55-fold in response to treatment in these aposymbiotic anemones, alongside smaller increases in 14,15-DiHETrE and mono-hydroxy- and *oxo*-forms of ARA and EPA (e.g. 11-HETE, 15-*oxo*-ETE, 11-HEPE).

### Impacts of thermal stress treatment on the symbiont

**Figure 8:**
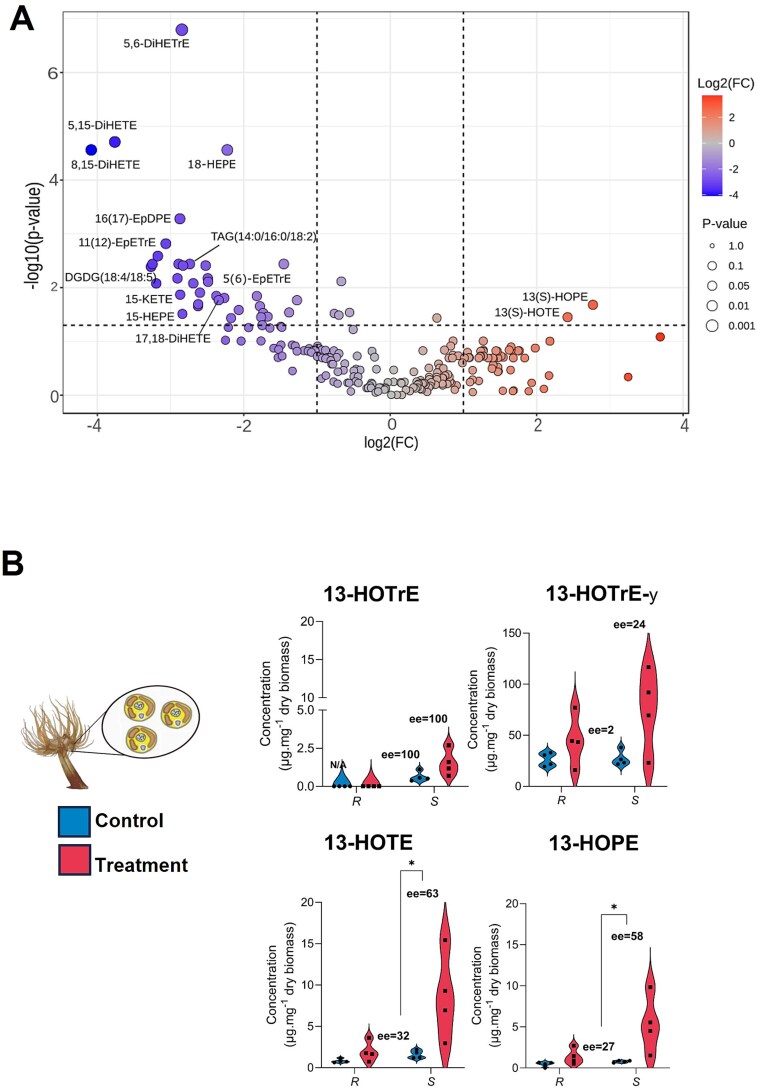
The effect of thermal stress on *Breviolum minutum*. (A) Oxylipin profiles (octadecanoids, eicosanoids, and docosanoids) and lipids when in symbiosis with Aiptasia in response to thermal stress treatment*.* The volcano plot shows 37 significantly different compounds between treatment (elevated temperature; *N* = 4) vs. control (25°C; *N* = 4) (FC > 2; *P* < 0.05 with FDR) (see Table S10 for detailed list of compounds). Upregulated and downregulated compounds are shown in red and blue, respectively. Nonsignificant compounds are shown in grey. (B) Abundance of *R* and *S* stereoisomers from different 13-regioisomers of mono-hydroxy octadecanoids. Enantiomeric excess values are presented as % ee for control and treatment. Box plots show average concentrations (μg.mg^−1^ of symbiont dry biomass) with error bars showing SE (*N* = 4). Nomenclature: 13-hydroxyoctadecatrienoic acid (13-HOTrE) from ALA (18:3, *n*−3); 13-hydroxyoctadecatrienoic acid gamma (13-HOTrE-γ) from GLA (18:3, *n*−6); 13-hydroxy-octadecatetraenoic acid (13-HOTE) from SDA (18:4, *n*−3); 13-hydroxy-octadecapentaenoic acid (13-HOPE) from ODPA (18:5, *n*−3).

Our observations were consistent with early stages of thermal stress for *B. minutum in hospite*, characterized by a decline in *F*_v_/*F*_m_ from 0.59 to 0.42 and a 30% decrease in chlorophyll-*a* content when normalized to symbiont dry biomass (Fig. 6C). Similarly, chloroplast membrane mono- and di-galactosyldiacylglycerol glycolipids (MGDG and DGDG) with SDA and ODPA acyl chains decreased by 25% and 50%, respectively, in the symbionts in response to thermal stress (*P* < .05 with FDR) (Fig. 8A; Table S10). The 13(*S*) mono-hydroxy metabolites of SDA and ODPA (13(*S*)-HOTE and 13(*S*)-HOPE, respectively) were the only two symbiont octadecanoids that increased in concentration, by 6-fold in both cases (Fig. 8B; Table S10). Their 50% increase in ee value also suggested higher 13*S*-LOX activity with thermal stress (Fig. 8B; Table S10). Other phenotypic changes in the symbionts with treatment were an acute drop in the abundance of epoxides and vicinal diols from the CYP and sEH pathways, such as a 92% decline in 5,6-DiHETrE levels (Fig. 8A; Table S10). The abundance of other mono-hydroxy and non-vicinal diols putatively associated with 5- and 15-LOX activity (e.g. 15-HETrE, 5-HETE, and 5,15-DiHETE), which use C20 as a substrate (DGLA, ARA, and EPA, respectively), also decreased by as much as 70% under treatment (Fig. 8A; Table S10); however, the chirality was not determined for these compounds.

## Discussion

Our study comprehensively profiled changes to the lipidome and oxylipin signatures of the Aiptasia–*Breviolum minutum* symbiosis across different symbiotic states and after a thermal stress. These data were then used to infer potential oxylipin biosynthesis pathways. Symbiosis led to marked host enrichment with symbiont-derived lipids and oxylipins, supporting their potential role in nutrition and immune regulation, respectively. Minor physiological and lipidomic alterations in the host after thermal treatment suggested greater thermal resilience in the symbiotic versus aposymbiotic state. Indeed, aposymbiotic animals displayed intense physiological dampening and dysregulation of lipid metabolism, marked by oxylipin profiles consistent with an upregulated immune response. In contrast to the symbiotic host, the dinoflagellate symbionts exhibited physiological quiescence and dysregulation of their lipid profiles. These events are consistent with previous evidence [15, 42–44] that symbiont dysfunction may be a primary driver of the bleaching cascade in the cnidarian–dinoflagellate symbiosis, although this remains an active area of debate.

### Lipid and oxylipin signatures of symbiosis

In Aiptasia, symbiosis was marked by an increased abundance of lipids and *n*−3 PUFAs (rather than *n*−6 PUFAs) that are thought to originate in the symbiont. Indeed, *n*−3 PUFAs have been used previously as biomarkers of trophic regime in corals and other cnidarians, where they reflect either a greater contribution of translocated photosynthate to the energy budget of the host [20, 45, 46] or the digestion of symbiont cells by the host [47]. In our study, only Lyso DGCC-DHA was detected in symbiotic but not in aposymbiotic anemones, suggesting that it, or its precursors, are derived from the symbiont. Both -lyso and -diacyl forms of DGCC are known to be especially abundant in Symbiodiniaceae [14] and other marine microalgae [48, 49], although both have also been reported for symbiotic cnidarian holobionts when host and symbiont fractions are analysed together [50–52]. Lysolipids are highly bioactive, binding to protein carriers and rapidly translocated across membranes [53]. Studies in mammals have pointed to their role in non-vesicular lipid transport for organelle biogenesis, membrane remodelling, and control of lipid homeostasis, especially under stress when vesicular lipid transport is compromised [54]. In the cnidarian–dinoflagellate symbiosis, the role of lysolipids and betaine lipids is unknown and might be a promising subject for future studies, particularly given the importance of understanding stress physiology.

Symbiosis was also marked by an up to 300-fold enrichment of epoxides and vicinal diols of eicosanoids and docosanoids suggestive of CYP and sEH enzymes activity, relative to the aposymbiotic state, consistent with previously observed patterns of CYP upregulation in Aiptasia observed in proteomics studies [55, 56]. The immunomodulatory role of oxylipins and other products of lipid peroxidation are mediated via their molecular binding to membrane receptors (i.e. G-protein coupled, GPCR), which, when activated, can trigger downstream nuclear transcription of inflammatory signatures, such as nuclear factor κB (NF-κB) [57]. Simultaneously, the same regulation at the transcription level can be provided by direct binding of oxylipins to nuclear receptors (i.e. peroxisome proliferator-activator receptors, PPARs), such as PPAR-γ, which have strong binding affinity with epoxides and vicinal diols [57, 58]. Other oxylipins, such as mono-hydroxy forms of octadecanoids, including the 13(*S*)-HOTE, have been identified as PPAR-γ ligands and immunoregulators of inflammatory cascades in mammals, showing that different oxylipins can bind to the same receptor even though their binding affinity is variable [59–61]. We previously proposed that this mechanism operates in the cnidarian–dinoflagellate symbiosis via PPAR-γ activation [19], which is known to also downregulate the transcription of NF-κB and potentially suppress the immune response of the host cnidarian [62–64]. Our data show the same pattern for 13(*S*)-HOTE and suggest a similar agonist role for 13(*S*)-HOPE, a putative 13*S*-LOX-derived product of ODPA that we report here for the first time. The high enantiomeric excess values observed for 13(*S*)-HOPE, together with the well-documented substrate permissibility of LOX enzymes across C18 PUFA substrates [65], support an enzymatic origin because non-enzymatic autoxidation characteristically yields racemic mixtures. Experimental confirmation of this specific LOX activity remains an important objective for future work.

Oxylipins arising from LOX and CYP oxidation pathways can bind to GPCRs [66], but they are often reported to have low or absent affinity, whereas COX-derived prostaglandins are strong GPCR agonists [67, 68]. Among these, prostaglandin-E receptors (EPs) are the most well-described GPCRs, and in cnidarians only the presence of EP2 and EP4 subtypes have been experimentally validated [69, 70]. In mammals, the affinity for these subtypes differs, as do the mechanisms by which they activate or suppress the propagation of signalling cascades (reviewed in [71]). In cnidarians, the molecular coupling of prostaglandins with receptors remains to be demonstrated; however, all three prostaglandins increased in symbiotic versus aposymbiotic anemones, with PGE_1_ levels 2.2-fold greater than PGE_2_ and PGE_3._ All three prostaglandins compete for GPCR receptor binding in Aiptasia, as in mammals and other higher metazoans, with the activation or suppression of the cnidarian immune system determined quantitatively at the oxylipin substrate level [72, 73]. We propose that shifts in the relative abundance of prostaglandins (PGE_1_, PGE_2_, PGE_3_) and other oxylipins during symbiosis modulate host immune tone, as in higher metazoans [71]. Although PGE_2_ may act as a pro-inflammatory signal through EP-like GPCRs [74, 75], PGE_1_ and PGE_3_ can exert anti-inflammatory effects [76, 77] possibly through biased signalling at the same receptor or via, yet unknown, pathways [71]. Given the conservation of the innate immune system of cnidarians and higher metazoans [25, 78], 13*S*-LOX- and CYP-derived mediators, in combination with prostaglandins, can all influence the host immune tone via crosstalk or metabolic competition, all collectively favouring an immunosuppressive state conducive to symbiosis stability.

Oxylipins are downstream products of fatty acid oxidation, which renders their metabolic and signalling pathways intrinsically co-related. This may in part explain why some corals, when exposed to omega-3 enriched diets (the most abundant fatty acids in marine zooplankton [79]), can recover from bleaching events by heterotrophic feeding [80, 81]. Indeed, fat reserves in host tissues have been suggested as an indicator of mortality risk in corals following thermal bleaching [82]. More specifically, the fatty acid class (*n*−3 vs. *n*−6) can determine cellular fate because the downstream oxidation products (e.g. oxylipins) and signalling cascades play a role in the control of host inflammation and immunity, as widely described for higher metazoans and humans (reviewed in [83, 84]). This hypothesis deserves more attention, not least given its relevance for developing nutraceuticals for coral restoration [6, 85].

### Unresolved prostaglandin pathways

The biosynthesis of COX-derived prostaglandins has been widely studied in cnidarians [20–22, 26], though many repository sequences contain incorrect annotations, as demonstrated here by verifying the presence of diagnostic sequence motifs. In dinoflagellates and other microalgal species, the presence and potential roles of COX and prostaglandins remain largely unknown. In diatoms, combined metabolite profiling and gene homology have demonstrated prostaglandin production under nutrient limitation, in stationary or senescent phases, and in response to grazers, suggesting a role in stress responses and cell–cell signalling [86, 87]. Similar pathways have been proposed in dinoflagellates, including the Symbiodiniaceae [88], although quantification of prostaglandin synthesis has been lacking until now. In our study, most prostaglandins were absent in *B. minutum* cultures across multiple conditions, including in nutrient-limited f/400 medium. All PGEs and PGDs monitored in our platform were detected in *B. minutum* when in symbiosis with Aiptasia, pointing to a role in inter-partner signalling. Supporting this hypothesis, our transcriptomic analyses revealed putative actively expressed biosynthesis candidates for cytosolic prostaglandin-E synthase (cPTGES) in both cultured and symbiotic *B. minutum*. Both cPTGES and microsomal mPTGES convert prostaglandin H_2_ (PGH_2_) into PGE_2_. PGH_2_ itself is produced from PGG_2_ through COX activity on ARA, which is less abundant in Symbiodiniaceae than other fatty acids [14]. Our homology searches did not identify canonical mammalian-like COX enzymes in Symbiodiniaceae, nor in Aiptasia. Instead, the intermediate biosynthesis may involve an uncharacterized enzyme from the CYP superfamily, or non-enzymatic free radical cyclization and peroxidation that generate prostaglandin analogues [89, 90], though these pathways need further investigation and experimental validation.

Our phylogenetic analysis identified cPTGES exclusively in both Symbiodiniaceae and Aiptasia, whereas mPTGES was recovered in other cnidarian species. These two enzymes belong to entirely distinct protein families: cPTGES is a member of the glutathione *S*-transferase superfamily, with *Galdieria sulphuraria* at the base of the cytosolic lineage, whereas mPTGES belongs to the MAPEG family, with *Auxenochlorella protothecoides* at the base of the microsomal lineage. Notably, neither *G. sulphuraria* nor *A. protothecoides* encode canonical COX sequences. The independent evolutionary origins of both synthase families in organisms lacking canonical COX sequences suggests that COX-independent PGE_2_ biosynthesis may represent an ancestral feature of non-metazoan eicosanoid pathways, consistent with convergent biochemical recruitment and the conservation of lipid signalling across biological systems [87, 91, 92]. In eukaryotic cells, cPTGES (also reported as p23 protein in protists, fungi, and plants) activity is regulated through post-translational modifications and complex formation with heat shock protein 90 (Hsp90) (78–80). Both PTGES forms additionally require glutathione (GSH) as a cofactor to stabilize the active site conformation for enzyme activation (81,82). In symbiosis, alterations to GSH availability and cellular redox balance may further regulate PTGES activity, providing a mechanistic basis for our observation that prostaglandin production in *B. minutum* is strongly influenced by symbiotic state. These findings underscore the need for further studies to confirm endogenous PGE_2_ synthesis and other oxylipin pathways in both host and symbiont, and to evaluate their role as a potential mechanism of thermal resilience in the holobiont, given the functional link between PTGES and Hsp90. Critically, this biosynthetic novelty was accessible only through a top-down strategy because the prior detection of PGE_2_ anchors the functional relevance of the putative PTGES candidates, rather than the repository annotations which commonly lack molecular evidence of the end product.

### Onset of thermal bleaching

The photophysiological impacts of thermal stress on the symbionts were consistent with previous reports of early stages of bleaching [15, 42–44]. Our experimental changes only led to minor physiological and molecular phenotypic alterations in Aiptasia. Importantly, the substrate levels of oxylipins potentially involved in symbiosis homeostasis, as discussed above, remained unchanged. This indicates that, within the context of our experimental conditions, the early stages of the thermal bleaching cascade only involve metabolic adjustments that are insufficient to trigger measurable changes in inter-partner lipid signalling in the host and that the individual levels of fatty acids are not the primary bleaching drivers. In contrast, aposymbiotic anemones were severely physiologically impacted, as suggested by the up to 55-fold increase of the inflammatory mediators TXB_2_ and TXB_3_, the stable forms of thromboxane A_2_ and A_3_ (TXA_2_ and TXA_3_), respectively. To our knowledge, this is the first report of the formation of thromboxanes in a cnidarian–dinoflagellate system. The enzyme TXS produces thromboxane and the mono-hydroxy 12(*S*)-hydroxy-5*Z*,8*E*,10*E*-heptadecatrienoic acid (12-HHTrE) in a 1:1 ratio from ARA [41]. In our current data, we observed increased levels of both TXB_2_ and 12-HHTrE (Table S7), further supporting the hypothesis that Aiptasia possesses TXS activity, but the route of formation of thromboxanes in both Aiptasia and Symbiodiniaceae remains to be resolved. TXA_2_ is a potent agonist of thromboxane-prostanoid-GPCRs, which activate platelets and induce vasoconstriction in mammals [32, 93] and may disrupt cellular homeostasis while amplifying immune responses linked to apoptosis in the cnidarian–dinoflagellate symbiosis. The oxylipin profile of aposymbiotic Aiptasia under elevated temperature strongly supports a connection between thermal stress and apoptosis that is independent of the algal symbionts, as previously suggested for this model [4, 62, 94].

For the symbiont, onset of thermal stress was apparent from the decreases in *F*_v_/*F*_m_ and chlorophyll-*a* and chloroplast membrane-specific lipid content. Alterations to lipid phenotype and oxylipin accumulation also corroborated this interpretation, notably because there was an increased abundance of both 13(*S*)-HOTE and 13(*S*)-HOPE. These increases might have resulted from upregulated 13*S*-LOX activity [34] and the oxidation of SDA and ODPA in their chloroplast membranes. Indeed, in plants, 13*S*-LOX activity increases in response to thermal stress until an optimum threshold is reached, after which it collapses [95]; the same might be the case for Symbiodiniaceae. In addition, decreased abundance of epoxides and vicinal diols potentially associated with CYP and sEH activity was observed in the symbionts under thermal stress. These enzymes have yet to be characterized for Symbiodiniaceae, but a few candidates have been reported [96–98]. In plants and other algae, the CYP74 superfamily includes allene oxide, epoxy alcohol, and divinyl ether synthases [65, 99], which may similarly be involved in the synthesis of the symbiont epoxides and diols observed in this study. For instance, reduced abundance of CYP38 proteins and CYP gene transcripts under thermal stress has been reported both when Symbiodiniaceae are in culture [97] and in symbiosis with corals [98], at 34 and 32°C, respectively. The temperature in our study peaked at 33.5°C, rendering a downregulation or denaturation of CYP enzymes in *B. minutum* plausible. Understanding the bioactivity and binding affinity of 13(*S*) octadecanoids, as well as the source of epoxide and vicinal diol synthesis, and identification of their specific receptors in the system, are promising goals for future work.

## Conclusion

This study proposes that symbiosis homeostasis and thermal stability is mediated by the temporal and spatial fine-tuning of specific oxylipins, which we suggest as targets for functional tests using inhibitors and gene editing to hinder bleaching cascades in controlled trials (Table 1). We discuss lipids, not only as key substrates for coral nutrition, but also as determinants of cellular fate via modulation of signalling pathways, across cnidarians and higher metazoans, including humans. This concept warrants further exploration with respect to the development of nutraceutical interventions for coral restoration. Our phenotypic screening, particularly the stereochemical resolution, provides a functional starting point for hypothesis-driven comparative genomics/transcriptomics of different symbioses. These tools may also be applicable to support the selective breeding and assisted evolution of more thermally resistant host–symbiont pairings, helping to secure the future of coral reefs.

**Table 1:** Oxylipins that potentially act as mediators of the cnidarian–dinoflagellate symbiosis and their biosynthesis and signalling pathways.

Oxylipin	Fatty acid substrate^a^	Synthesis pathway	Source	Potential receptor	Suggested bioactivity
Octadecanoids (C18)					
9(*R*),10(*S*),13(*R*)-TriHOME	LA (18:2, *n*−6)	LOX; AOS-LOX	Symbiont	Unknown	Support of symbiosis homeostasis
13(*S*)-HOTE	SDA (18:4, *n*−3)	13S-LOX	Symbiont	PPAR-γ; unknown	Support of symbiosis homeostasis and thermal resilience
13(*S*)-HOPE	ODPA (18:5, *n*−3)	13S-LOX	Symbiont	PPAR-γ; unknown	Support of symbiosis homeostasis and thermal resilience
Eicosanoids (C20)					
Epoxides and vicinal diols	DGLA (20:3, *n*−6) ARA (20:4, *n*−6) EPA (20:5, *n*−3)	CYP/sEH	Symbiont and host	High binding affinity with PPAR-γ; unknown	Support of symbiosis homeostasis and thermal resilience
PGE_1_	DGLA (20:3, *n*−6)	COX/PTGES	Host >> symbiont	GPCR: EP1 and EP4; unknown	Support of symbiosis homeostasis
PGE_2_	ARA (20:4, *n*−6)	COX/PTGES	Host	GPCR: EP1 and EP4; unknown	Physiological stress; activated immune response
PGE_3_	EPA (20:5, *n*−3)	COX/PTGES	Host >> symbiont	GPCR: EP1 and EP4; unknown	Support of symbiosis homeostasis
TXB_1_	DGLA (20:3, *n*−6)	COX/TXS	Host >> symbiont	Unknown	Support of symbiosis homeostasis
TXB_2_	ARA (20:4, *n*−6)	COX/TXS	Host	Unknown	Physiological stress; activated immune response
TXB_3_	EPA (20:5, *n*−3)	COX/TXS	Host >> symbiont	Unknown	Support of symbiosis homeostasis
Docosanoids (C22)					
Epoxides and vicinal diols	DHA (22:6, *n*−3)	CYP/sEH	Host >> symbiont	High binding affinity with PPAR-γ; unknown	Support of symbiosis homeostasis and thermal resilience

^a^LA = linoleic acid. SDA = stearidonic acid; ODPA = octadecapentaenoic acid; DGLA = dihomo-γ-linolenic acid; ARA = arachidonic acid; EPA = eicosapentaenoic acid; DHA = docosahexaenoic acid; LOX = lipoxygenase; AOS-LOX = allene-oxide synthase lipoxygenase; CYP = cytochrome P450; sEH = soluble epoxidase hydrolase; COX = cyclooxygenase; PTGES = prostaglandin-E synthase; TXS = thromboxane synthase; PPAR-γ = peroxisome proliferator-activator receptors; GPCR = G-protein coupled receptors.

## Supplementary Material

Supplementary_material_wrag143

## Data Availability

All raw untargeted mass spectrometry data (lipidomics) generated in this study are publicly available in the MassIVE repository under accession number MSV000101404 (MassIVE MSV000101404). The mRNA-enriched RNAseq libraries used for *de novo* transcriptome assembly were previously published and their accession numbers are listed in Table S3. All other data generated or analysed during this study are included in this published article and its supplementary information files.

## References

[1] Jacobovitz MR, Rupp S, Voss PA, et al. Dinoflagellate symbionts escape vomocytosis by host cell immune suppression. *Nat Microbiol* 2021;6:769–82. 10.1038/s41564-021-00897-w 33927382 PMC7611106

[2] Lehnert EM, Mouchka ME, Burriesci MS, et al. Extensive differences in gene expression between symbiotic and aposymbiotic cnidarians. *G3 GenesGenomesGenetics* 2014;4:277–95. 10.1534/g3.113.009084 PMC3931562 24368779

[3] Rosset SL, Oakley CA, Ferrier-Pagès C, et al. The molecular language of the cnidarian–dinoflagellate symbiosis. *Trends Microbiol* 2021;29:320–33. 10.1016/j.tim.2020.08.005 33041180

[4] Helgoe J, Davy SK, Weis VM, et al. Triggers, cascades, and endpoints: connecting the dots of coral bleaching mechanisms. *Biol Rev* 2024;99:715–52. 10.1111/brv.13042 38217089

[5] Oakley CA, Davy SK. Cell biology of coral bleaching. In: Van Oppen M.J.H., Lough J.M. (eds.), Coral Bleaching, Ecological Studies. Cham: Springer International Publishing, 2018, 233.189–211.

[6] Chille EE, Stephens TG, Nandi S, et al. Coral restoration in the omics era: development of point-of-care tools for monitoring disease, reproduction, and thermal stress. *BioEssays* 2025;47:e70007. 10.1002/bies.70007 40285547 PMC12101048

[7] Sully S, Burkepile DE, Donovan MK, et al. A global analysis of coral bleaching over the past two decades. *Nat Commun* 2019;10:1264. 10.1038/s41467-019-09238-2 30894534 PMC6427037

[8] Lee DS, Nioche P, Hamberg M, et al. Structural insights into the evolutionary paths of oxylipin biosynthetic enzymes. *Nature* 2008;455:363–8. 10.1038/nature07307 18716621

[9] Walley JW, Kliebenstein DJ, Bostock RM, et al. Fatty acids and early detection of pathogens. *Curr Opin Plant Biol* 2013;16:520–6. 10.1016/j.pbi.2013.06.011 23845737

[10] Mosblech A, Feussner I, Heilmann I. Oxylipins: structurally diverse metabolites from fatty acid oxidation. *Plant Physiol Biochem* 2009;47:511–7. 10.1016/j.plaphy.2008.12.011 19167233

[11] Mueller MJ . Archetype signals in plants: the phytoprostanes. *Curr Opin Plant Biol* 2004;7:441–8. 10.1016/j.pbi.2004.04.001 15231268

[12] Yin H, Xu L, Porter NA. Free radical lipid peroxidation: mechanisms and analysis. *Chem Rev* 2011;111:5944–72. 10.1021/cr200084z 21861450

[13] Goyen S, Pernice M, Szabó M, et al. A molecular physiology basis for functional diversity of hydrogen peroxide production amongst Symbiodinium spp. (Dinophyceae). *Mar Biol* 2017;164:46. 10.1007/s00227-017-3073-5

[14] M TB, Chaves-Filho AB, Inague A, et al. Thermal plasticity of coral reef symbionts is linked to major alterations in their lipidome composition. *Limnol Oceanogr* 2022;67:1456–69. 10.1002/lno.12094

[15] Lesser MP . Oxidative stress causes coral bleaching during exposure to elevated temperatures. *Coral Reefs* 1997;16:187–92. 10.1007/s003380050073

[16] Tchernov D, Gorbunov MY, de Vargas C. et al. Membrane lipids of symbiotic algae are diagnostic of sensitivity to thermal bleaching in corals. Proc Natl Acad Sci 2004;101(37):13531–5. 10.1073/pnas.0402907101. 15340154 PMC518791

[17] Rädecker N, Pogoreutz C, Gegner HM, et al. Heat stress destabilizes symbiotic nutrient cycling in corals. *Proc Natl Acad Sci* 2021;118:e2022653118. 10.1073/pnas.2022653118 33500354 PMC7865147

[18] Botana MT . Lipid Mediators and a New HOPE in the Cnidarian-Dinoflagellate Symbiosis, Te Herenga-Waka/Victoria University of Wellington, New Zealand, 2025.

[19] Botana MT, Lewis RE, Quaranta A, et al. Octadecanoids as emerging lipid mediators in cnidarian-dinoflagellate symbiosis. *Commun Biol* 2025;8:1519. 10.1038/s42003-025-09104-6 41188575 PMC12586643

[20] Imbs AB, Dembitsky VM. Coral lipids. *Mar Drugs* 2023;21:539. 10.3390/md21100539 37888474 PMC10608786

[21] Lõhelaid H, Järving R, Valmsen K, et al. Identification of a functional allene oxide synthase-lipoxygenase fusion protein in the soft coral *Gersemia fruticosa* suggests the generality of this pathway in octocorals. *Biochim Biophys Acta BBA - Gen Subj* 2008;1780:315–21. 10.1016/j.bbagen.2007.10.010 17996204

[22] Lõhelaid H, Samel N. Eicosanoid diversity of stony corals. *Mar Drugs* 2018;16:10. 10.3390/md16010010 29301345 PMC5793058

[23] Dyall SC, Balas L, Bazan NG, et al. Polyunsaturated fatty acids and fatty acid-derived lipid mediators: recent advances in the understanding of their biosynthesis, structures, and functions. *Prog Lipid Res* 2022;86:101165. 10.1016/j.plipres.2022.101165 35508275 PMC9346631

[24] Kvitt H, Rosenfeld H, Tchernov D. The regulation of thermal stress induced apoptosis in corals reveals high similarities in gene expression and function to higher animals. *Sci Rep* 2016;6:30359. 10.1038/srep30359 27460544 PMC4961959

[25] Palmer CV, Traylor-Knowles N. Towards an integrated network of coral immune mechanisms. *Proc R Soc B Biol Sci* 2012;279:4106–14. 10.1098/rspb.2012.1477 PMC3441085 22896649

[26] Brash AR, Boeglin WE, Chang MS, et al. Purification and molecular cloning of an 8R-lipoxygenase from the coral *Plexaura homomalla* reveal the related primary structures of R- and S-lipoxygenases. *J Biol Chem* 1996;271:20949–57. 10.1074/jbc.271.34.20949 8702854

[27] Imbs AB, Yakovleva IM, Pham LQ. Distribution of lipids and fatty acids in the zooxanthellae and host of the soft coral Sinularia sp. *Fish Sci* 2010;76:375–80. 10.1007/s12562-009-0213-y

[28] Valmsen K, Järving I, Boeglin WE, et al. The origin of 15 *R* -prostaglandins in the Caribbean coral *Plexaura homomalla*: molecular cloning and expression of a novel cyclooxygenase. *Proc Natl Acad Sci* 2001;98:7700–5. 10.1073/pnas.131022398 11427702 PMC35405

[29] Matthews JL, Sproles AE, Oakley CA, et al. Menthol-induced bleaching rapidly and effectively provides experimental aposymbiotic sea anemones (*Aiptasia* sp.) for symbiosis investigations. *J Exp Biol* 2015;219:306–310. 10.1242/jeb.128934 26596538

[30] Goulet T, Coffroth M. Genetic composition of zooxanthellae between and within colonies of the octocoral *Plexaura kuna*, based on small subunit rDNA and multilocus DNA fingerprinting. *Mar Biol* 2003;142:233–9. 10.1007/s00227-002-0936-0

[31] Guillard RRL, Ryther JH. Studies of marine planktonic diatoms: I. *Cyclotella nana* Hustedt, and *Detonula confervacea* (Cleve) Gran. *Can J Microbiol* 2011;8:229–39. 10.1139/m62-029 13902807

[32] Offermanns S . Activation of platelet function through G protein–coupled receptors. *Circ Res* 2006;99:1293–304. 10.1161/01.RES.0000251742.71301.16 17158345

[33] Quaranta A, Zöhrer B, Revol-Cavalier J, et al. Development of a chiral supercritical fluid chromatography–tandem mass spectrometry and reversed-phase liquid chromatography–tandem mass spectrometry platform for the quantitative metabolic profiling of octadecanoid oxylipins. *Anal Chem* 2022;94:14618–14626. 10.1021/acs.analchem.2c02601 PMC9607849 36219822

[34] Botana MT, Lewis RE, Quaranta A, et al. Octadecanoids as emerging lipid mediators in cnidarian-dinoflagellate symbiosis. *Commun Biol*, 2025;8:1519. 10.1038/s42003-025-09104-6 PMC12586643 41188575

[35] Emms DM, Kelly S. OrthoFinder: phylogenetic orthology inference for comparative genomics. *Genome Biol* 2019;20:238. 10.1186/s13059-019-1832-y 31727128 PMC6857279

[36] Jones P, Binns D, Chang HY, et al. InterProScan 5: genome-scale protein function classification. *Bioinformatics* 2014;30:1236–40. 10.1093/bioinformatics/btu031 24451626 PMC3998142

[37] Edgar RC . MUSCLE: multiple sequence alignment with high accuracy and high throughput. *Nucleic Acids Res* 2004;32:1792–7. 10.1093/nar/gkh340 15034147 PMC390337

[38] Posada D, Crandall KA. Felsenstein phylogenetic likelihood. *J Mol Evol* 2021;89:134–45. 10.1007/s00239-020-09982-w 33438113 PMC7803665

[39] Le SQ, Gascuel O. An improved general amino acid replacement matrix. *Mol Biol Evol* 2008;25:1307–20. 10.1093/molbev/msn067 18367465

[40] Pang Z, Lu Y, Zhou G, et al. MetaboAnalyst 6.0: towards a unified platform for metabolomics data processing, analysis and interpretation. *Nucleic Acids Res* 2024;52:398–406. 10.1093/nar/gkae253 PMC11223798 38587201

[41] Hamberg M . Transformations of 5,8,11,14,17-eicosapentaenoic acid in human platelets. *Biochim Biophys Acta BBA - Lipids Lipid Metab* 1980;618:389–98. 10.1016/0005-2760(80)90257-X 6249377

[42] Nandi S, Stephens TG, Chille EE, et al. Metaproteome analysis of the coral holobiont demonstrates algal symbiont dampening under thermal stress. *Eco and Evol,* 2026;16:e73275. 10.1002/ece3.73275 PMC13093290 42016983

[43] Wang C, Zheng X, Kvitt H, et al. Lineage-specific symbionts mediate differential coral responses to thermal stress. *Microbiome* 2023;11:211. 10.1186/s40168-023-01653-4 37752514 PMC10521517

[44] Xiao L, Johansson S, Rughöft S, et al. Photophysiological response of Symbiodiniaceae single cells to temperature stress. *ISME J* 2022;16:2060–2064. 10.1038/s41396-022-01243-6 PMC9296599 35474114

[45] Papina M, Meziane T, van WR. Symbiotic zooxanthellae provide the host-coral Montipora digitata with polyunsaturated fatty acids. *Comp Biochem Physiol B Biochem Mol Biol* 2003;135:533–7. 10.1016/S1096-4959(03)00118-0 12831773

[46] Luo YJ, Wang LH, Chen WNU, et al. Ratiometric imaging of gastrodermal lipid bodies in coral–dinoflagellate endosymbiosis. *Coral Reefs* 2009;28:289–301. 10.1007/s00338-008-0462-8

[47] Wiedenmann J, D’Angelo C, Mardones ML, et al. Reef-building corals farm and feed on their photosynthetic symbionts. *Nature* 2023;620:1018–24. 10.1038/s41586-023-06442-5 37612503 PMC10468396

[48] Becker KW, Collins JR, Durham BP, et al. Daily changes in phytoplankton lipidomes reveal mechanisms of energy storage in the open ocean. *Nat Commun* 2018;9:5179. 10.1038/s41467-018-07346-z 30518752 PMC6281602

[49] Holm HC, Fredricks HF, Bent SM, et al. Global ocean lipidomes show a universal relationship between temperature and lipid unsaturation. *Science* 2022;376:1487–91. 10.1126/science.abn7455 35737766

[50] Chan WY, Rudd D, van OMJ. Spatial metabolomics for symbiotic marine invertebrates. *Life Sci Alliance* 2023;6:e202301900. 10.26508/lsa.202301900 37202120 PMC10200813

[51] Roach TNF, Dilworth J, CM H, et al. Metabolomic signatures of coral bleaching history. *Nat Ecol Evol* 2021;5:495–503. 10.1038/s41559-020-01388-7 33558733

[52] Roach TNF, Drury C, Caruso C, et al. Intergenerational metabolomic signatures of bleaching resistance in corals. *Nat Commun* 2025;16:5971. 10.1038/s41467-025-61102-8 PMC12214687 40593676

[53] Tan ST, Ramesh T, Toh XR, et al. Emerging roles of lysophospholipids in health and disease. *Prog Lipid Res* 2020;80:101068. 10.1016/j.plipres.2020.101068 33068601

[54] Reinisch KM, Prinz WA. Mechanisms of nonvesicular lipid transport. *J Cell Biol* 2021;220:e202012058. 10.1083/jcb.202012058 33605998 PMC7901144

[55] Oakley CA, Ameismeier MF, Peng L, et al. Symbiosis induces widespread changes in the proteome of the model cnidarian Aiptasia. *Cell Microbiol* 2016;18:1009–23. 10.1111/cmi.12564 26716757

[56] Pankov KV, McArthur AG, Gold DA, et al. The cytochrome P450 (CYP) superfamily in cnidarians. *Sci Rep* 2021;11:9834. 10.1038/s41598-021-88700-y 33972594 PMC8110760

[57] Liu T, Zhang L, Joo D, et al. NF-κB signaling in inflammation. *Signal Transduct Target Ther* 2017;2:17023. 10.1038/sigtrans.2017.23 29158945 PMC5661633

[58] Liu Y, Zhang Y, Schmelzer K, et al. The antiinflammatory effect of laminar flow: the role of PPARγ, epoxyeicosatrienoic acids, and soluble epoxide hydrolase. *Proc Natl Acad Sci* 2005;102:16747–52. 10.1073/pnas.0508081102 16267130 PMC1276614

[59] Ávila-Román J, Talero E, de Los Reyes C, et al. Microalgae-derived oxylipins decrease inflammatory mediators by regulating the subcellular location of NFκB and PPAR-γ. *Pharmacol Res* 2018;128:220–30. 10.1016/j.phrs.2017.10.009 29129670

[60] De Los RC, Ávila-Román J, Ortega MJ, et al. Oxylipins from the microalgae *Chlamydomonas debaryana* and *Nannochloropsis gaditana* and their activity as TNF-α inhibitors. *Phytochemistry* 2014;102:152–61. 10.1016/j.phytochem.2014.03.011 24703579

[61] Itoh T, Fairall L, Amin K, et al. Structural basis for the activation of PPARγ by oxidized fatty acids. *Nat Struct Mol Biol* 2008;15:924–31. 10.1038/nsmb.1474 19172745 PMC2939985

[62] Cleves PA, Krediet CJ, Lehnert EM, et al. Insights into coral bleaching under heat stress from analysis of gene expression in a sea anemone model system. *Proc Natl Acad Sci* 2020;117:28906–17. 10.1073/pnas.2015737117 33168733 PMC7682557

[63] Mansfield KM, Carter NM, Nguyen L, et al. Transcription factor NF-κB is modulated by symbiotic status in a sea anemone model of cnidarian bleaching. *Sci Rep* 2017;7:16025. 10.1038/s41598-017-16168-w 29167511 PMC5700166

[64] Wolfowicz I, Baumgarten S, Voss PA, et al. Aiptasia sp. larvae as a model to reveal mechanisms of symbiont selection in cnidarians. *Sci Rep* 2016;6:32366. 10.1038/srep32366 27582179 PMC5007887

[65] Wasternack C, Feussner I. The oxylipin pathways: biochemistry and function. *Annu Rev Plant Biol* 2018;69:363–86. 10.1146/annurev-arplant-042817-040440 29166128

[66] Meijer L, Brash AR, Bryant RW, et al. Stereospecific induction of starfish oocyte maturation by (8R)-hydroxyeicosatetraenoic acid. *J Biol Chem* 1986;261:17040–7. 10.1016/S0021-9258(19)75996-1 3097019

[67] Dennis EA, Norris PC. Eicosanoid storm in infection and inflammation. *Nat Rev Immunol* 2015;15:511–23. 10.1038/nri3859 26139350 PMC4606863

[68] Weis WI, Kobilka BK. The molecular basis of G protein–coupled receptor activation. *Annu Rev Biochem* 2018;87:897–919. 10.1146/annurev-biochem-060614-033910 29925258 PMC6535337

[69] Anctil M, Hayward DC, Miller DJ, et al. Sequence and expression of four coral G protein-coupled receptors distinct from all classifiable members of the rhodopsin family. *Gene* 2007;392:14–21. 10.1016/j.gene.2006.10.025 17196770

[70] Gamba AG, Oakley CA, Ashley IA, et al. Oxylipin receptors and their role in inter-partner signalling in a model cnidarian-dinoflagellate symbiosis. *Environ Microbiol* 2024;26:e70015. 10.1111/1462-2920.70015 39702992

[71] Fujino H . The biased activities of prostanoids and their receptors: review and beyond. *Biol Pharm Bull* 2022;45:684–90. 10.1248/bpb.b21-01052 35650096

[72] Schöneberg T . Modulating vertebrate physiology by genomic fine-tuning of GPCR functions. *Physiol Rev* 2025;105:383–439. 10.1152/physrev.00017.2024 39052017

[73] Wu C, Xu Y, He Q, et al. Ligand-induced activation and G protein coupling of prostaglandin F2α receptor. *Nat Commun* 2023;14:2668. 10.1038/s41467-023-38411-x 37160891 PMC10169810

[74] Fujino H . The roles of EP4 Prostanoid receptors in cancer malignancy signaling. *Biol Pharm Bull* 2016;39:149–55. 10.1248/bpb.b15-00840 26830476

[75] Hull MA, Ko SCW, Hawcroft G. Prostaglandin EP receptors: targets for treatment and prevention of colorectal cancer? *Mol Cancer Ther* 2004;3:1031–9. 10.1158/1535-7163.1031.3.8 15299086

[76] Araki Y, Suganami A, Endo S, et al. PGE _1_ and E _3_ show lower efficacies than E _2_ to β-catenin-mediated activity as biased ligands of EP 4 prostanoid receptors. *FEBS Lett* 2017;591:3771–80. 10.1002/1873-3468.12878 28986997

[77] Yang P, Jiang Y, Fischer SM. Prostaglandin E3 metabolism and cancer. *Cancer Lett* 2014;348:1–11. 10.1016/j.canlet.2014.03.010 24657656 PMC4366418

[78] Mansfield KM, Cleves PA, Van Vlack E, et al. Varied effects of algal symbionts on transcription factor NF-κB in a sea anemone and a coral: possible roles in symbiosis and thermotolerance. Preprint. *Mol Biol* 2019. 10.1101/640177

[79] Brett MT, Müller-Navarra DC, Persson J. Crustacean zooplankton fatty acid composition. In: Kainz M., Brett M.T., Arts M.T. (eds.), Lipids in Aquatic Ecosystems. New York, NY: Springer, 2009, 115–46.

[80] Grottoli AG, Rodrigues LJ, Palardy JE. Heterotrophic plasticity and resilience in bleached corals. *Nature* 2006;440:1186–9. 10.1038/nature04565 16641995

[81] Martinez S, Grover R, Ferrier-Pagès C. Unveiling the importance of heterotrophy for coral symbiosis under heat stress. *mBio* 2024;15:e01966–24. 10.1128/mbio.01966-24 39207106 PMC11481558

[82] Anthony KRN, Hoogenboom MO, Maynard JA, et al. Energetics approach to predicting mortality risk from environmental stress: a case study of coral bleaching. *Funct Ecol* 2009;23:539–50. 10.1111/j.1365-2435.2008.01531.x

[83] Goldmann O, Nwofor OV, Chen Q, et al. Mechanisms underlying immunosuppression by regulatory cells. *Front Immunol* 2024;15:1328193. 10.3389/fimmu.2024.1328193 38380317 PMC10876998

[84] Hu A, Sun L, Lin H, et al. Harnessing innate immune pathways for therapeutic advancement in cancer. *Signal Transduct Target Ther* 2024;9:68. 10.1038/s41392-024-01765-9 38523155 PMC10961329

[85] Hughes TP, Baird AH, Morrison TH, et al. Principles for coral reef restoration in the Anthropocene. *One Earth* 2023;6:656–65. 10.1016/j.oneear.2023.04.008

[86] Fontana A, d’Ippolito G, Cutignano A, et al. Chemistry of oxylipin pathways in marine diatoms. *Pure Appl Chem* 2007;79:481–90. 10.1351/pac200779040481

[87] Di Dato V, Barbarinaldi R, Amato A, et al. Variation in prostaglandin metabolism during growth of the diatom *Thalassiosira rotula*. *Sci Rep* 2020;10:5374. 10.1038/s41598-020-61967-3 32214130 PMC7096440

[88] Di Dato V, Ianora A, Romano G. Identification of prostaglandin pathway in dinoflagellates by transcriptome data mining. *Mar Drugs* 2020;18:109. 10.3390/md18020109 32069885 PMC7073720

[89] Gupta K, Selinsky BS. Bacterial and algal orthologs of prostaglandin H2 synthase: novel insights into the evolution of an integral membrane protein. *Biochim Biophys Acta Biomembr* 2015;1848:83–94. 10.1016/j.bbamem.2014.09.011 25281773

[90] Hardy KD, Cox BE, Milne GL, et al. Nonenzymatic free radical-catalyzed generation of 15-deoxy-Δ12,14-prostaglandin J2-like compounds (deoxy-J2-isoprostanes) in vivo. *J Lipid Res* 2011;52:113–24. 10.1194/jlr.M010264 20944061 PMC2999919

[91] Varvas K, Kasvandik S, Hansen K, et al. Structural and catalytic insights into the algal prostaglandin H synthase reveal atypical features of the first non-animal cyclooxygenase. *Biochim Biophys Acta BBA - Mol Cell Biol Lipids* 2013;1831:863–71. 10.1016/j.bbalip.2012.11.010 23220097

[92] Hansen K, Varvas K, Järving I, et al. Novel membrane-associated prostaglandin E synthase-2 from crustacean arthropods. *Comp Biochem Physiol B Biochem Mol Biol* 2014;174:45–52. 10.1016/j.cbpb.2014.05.004 24947207

[93] Tian Y, Zong Y, Pang Y, et al. Platelets and diseases: signal transduction and advances in targeted therapy. *Signal Transduct Target Ther* 2025;10:159. 10.1038/s41392-025-02198-8 40374650 PMC12081703

[94] Dunn SR, Pernice M, Green K, et al. Thermal stress promotes host mitochondrial degradation in symbiotic cnidarians: are the batteries of the reef going to run out? *PLoS One* 2012;7:e39024. 10.1371/journal.pone.0039024 22815696 PMC3398039

[95] Park JY, Kim CH, Choi Y, et al. Catalytic characterization of heterodimeric linoleate 13S-lipoxygenase from black soybean (Glycine max (L.) Merr.). *Enzym Microb Technol* 2020;139:109595. 10.1016/j.enzmictec.2020.109595 32732043

[96] Mashini A, Oakley CA, Peng L, et al. Proteomes of native and non-native symbionts reveal responses underpinning host-symbiont specificity in the cnidarian–dinoflagellate symbiosis. *ISME J* 2024;18:wrae122. 10.1093/ismejo/wrae122 38988135 PMC11473927

[97] Oakley CA, Newson GI, Peng L, et al. The *Symbiodinium* proteome response to thermal and nutrient stresses. *Plant Cell Physiol* 2023;64:433–47. 10.1093/pcp/pcac175 36565060 PMC10109209

[98] Rosic NN, Pernice M, Dunn S, et al. Differential regulation by heat stress of novel cytochrome P450 genes from the dinoflagellate symbionts of reef-building corals. *Appl Environ Microbiol* 2010;76:2823–9. 10.1128/AEM.02984-09 20228102 PMC2863455

[99] Schaller A, Stintzi A. Enzymes in jasmonate biosynthesis – structure, function, regulation. *Phytochemistry* 2009;70:1532–8. 10.1016/j.phytochem.2009.07.032 19703696

[100] Keeling PJ, Eglit Y. Openly available illustrations as tools to describe eukaryotic microbial diversity. *PLoS Biol* 2023;21:e3002395. 10.1371/journal.pbio.3002395 37988341 PMC10662721

