# Supplementary material for: Oxylipin-mediated metabolic signatures of symbiosis homeostasis and thermal stress in a model sea anemone
Source: ISME J. 2026 Jun 7;20(1):wrag143. doi: 10.1093/ismejo/wrag143 (PMC13440314; doi:10.1093/ismejo/wrag143)
Supplement: Supplementary file 1 [file supplementary_material_wrag143.zip › Botana_et_al_Supplementary_formatted_wrag143.pdf]

Supplemental Information

Oxylipin-mediated metabolic signatures of symbiosis homeostasis and thermal stress in a model sea anemone

Marina T. Botana[1,2], Robert E. Lewis[1], Kevin Mitchell[3], Olivier Salamin[2], Johanna Revol-Cavalier[2,4], Evan Heit[1], Andrea Gamba[1], Shaun P. Wilkinson[5], Sabrina L. Rosset[6,7], Clinton A. Oakley[1], Matthew R. Nitschke[1,8], Mats Hamberg[2,4], Arthur R. Grossman[9], David J. Suggett[10,11], Virginia M. Weis[12], Craig E. Wheelock[2,13]*, Simon K. Davy[1]*

[1]School of Biological Sciences, Victoria University of Wellington, Wellington 6012, New Zealand.

[2]Unit of Integrative Metabolomics, Institute of Environmental Medicine, Karolinska Institutet, 171 77 Stockholm, Sweden.

[3]Callaghan Innovation, 69 Gracefield Rd, Lower Hutt, 5010, New Zealand.

[4]Larodan Research Laboratory, Karolinska Institutet, 171 65 Stockholm, Sweden.

[5]Wilderlab, Miramar, Wellington, New Zealand.

[6]Hawai‘i Institute of Marine Biology, University of Hawai‘i at Manoa, Kāne‘ohe, HI, United States

[7]Department of Biochemistry and Molecular Biology, Michigan State University, East Lansing, MI, USA

[8]Australian Institute of Marine Science, 1526 Cape Cleveland Road, Cape Cleveland 4810, Queensland, Australia.

[9]Carnegie Institution for Science, Department of Plant Biology, Stanford, CA 94305, USA.

[10]KAUST Coral Restoration Initiative (KCRI) and Division of Biological and Environmental Science and Engineering (BESE), King Abdullah University of Science and Technology, Thuwal 23955, Saudi Arabia

[11]Climate Change Cluster (C3), University of Technology Sydney, Ultimo, NSW 2007, Australia

[12]Department of Integrative Biology, Oregon State University, Corvallis, OR 97331, USA.

[13]Department of Respiratory Medicine and Allergy, Karolinska University Hospital, 171 76 Stockholm, Sweden.

*co-corresponding authors
Craig Wheelock
Unit of Integrative Metabolomics
Institute of Environmental Medicine
Karolinska Institutet
171 77 Stockholm, Sweden
craig.wheelock@ki.se

Simon Davy
School of Biological Sciences
Victoria University of Wellington
Wellington 6012, New Zealand
simon.davy@vuw.ac.nz

Table of Contents

Methods

M1. Characterization of lipids and photosynthetic pigments using LC-MS DIA……….……….3
M2. Characterization and quantification of eicosanoids and docosanoids using LC-MS………...6
M3. *De novo* transcriptome assembly and mRNA abundance……………………….………...6

Figures

Fig. S1. Sequence alignment of novel putative Cyclooxygenases (COX) enzymes……………..8
Fig. S2. Phylogenetic tree of Cyclooxygenases (COX)……………………………………......9
Fig. S3. Sequence alignment of novel putative prostaglandin E-synthase (PTGES) enzymes….10
Fig. S4. Sequence alignment of novel putative Lipoxygenases (LOXes)……..………………...11
Fig. S5. Phylogenetic tree of Lipoxygenases (LOXes) ………………………………………12
Fig. S6. Sensitivity comparison between MS^E and SONAR-MS^E……………………...……13
Fig. S7. Structural characterization of glycolipids…………………...………………14
Fig. S8. Structural characterization of betaine lipids…………………………………...……..15
Fig. S9. Instrument linearity check……………………………………………..…………..16

Tables

Table S1. Species confirmation for *Breviolum minutum* ………………………...……..……17
Table S2. Detailed list of all screened compounds and their associated analytical platforms…..18
Table S3. Actively expressed enzymes and access numbers for the LOX phylogeny tree ..……27
Table S4. References for genomes and RNAseq libraries………………………….……..27
Table S5. Full names and abbreviations of oxylipins monitored in our study...……...…………28
Table S6. Quantification of all lipids, pigments, and oxylipins across all datasets ……………....35
Table S7. Classification of putative LOX and AOS-LOX sequences……………..………..35
Table S8. Lipids and oxylipins that were significantly altered with symbiotic state and elevated temperature in Aiptasia ………………………...…………………………..35
Table S9. Relative amounts (%) of R and S enantiomers and ee values for octadecanoids...…..36
Table S10. Lipids, pigments and oxylipins that were significantly altered with thermal stress in *Breviolum minutum* ……………………..………...36
Table S11. Description of internal standards used for semi-quantification of different lipid classes………………………………………………………………..……………37
Table S12. Lipid library in both negative and positive modes …………………….………...37
Table S13. Reproducibility of quality controls of lipidomics method in negative and positive modes……………...………………………………………………………...….37
Table S14. Description of additional standard used for the quantification of glycolipids with external calibration curve……………………...……………………………….…...38
Table S15. Linearity check of ionization response in lipidomics method……………………....39

References……………………………………………………………………..……...40

Methods

M1. Characterization of lipids and photosynthetic pigments using LC-MS DIA

Chemicals and reagents

Lipids used as internal standards, *e.g.,* 1,2-diheptadecanoyl-sn-glycero-3-phosphocholine (PC 17:0/17:0), 1,2-diheptadecanoyl-sn-glycero-3-phosphoethanolamine (PE 17:0/17:0), 1-heptadecanoyl-2-hydroxy-sn-glycero-3-phosphocoline (LPC-17:0), N-heptadecanoyl-D-erythro-sphingosine (Cer d18:1/17:0) and 1,2,3-triheptadecanoyl glycerol (TAG 17:0/17:0/17:0) and for construction of external calibration curve *e.g.,* monogalactosyl dilinolenoyl glycerol (MGDG 18:3/18:3), were all purchased from Avanti Polar Lipids Inc. (Alabaster, AL. USA). Ammonium acetate, sodium phosphate and deferoxamine mesylate salts were obtained from Sigma-Aldrich (St Louis, MO, USA), all LCMS grade organic solvents and HPLC grade water were obtained from Thermo Fisher Scientific (Waltham, MA, USA).

Sample preparation

Anemones were sampled with sterile pipettes and homogenized with 1 mL of ice cold 10 mM sodium phosphate buffer (pH 7.4) containing deferoxamine mesylate (100 µM) using a glass tissue grinder. Aposymbiotic anemone homogenates were flash-frozen, freeze-dried, and stored at -80°C in 1.5 mL sterile Eppendorf tubes. Homogenates of symbiotic anemone were transferred to sterile Eppendorf tubes and centrifuged at 500 x *g* for 5 min at 4°C, for separation of host and symbiont fractions. Host supernatant biomass was transferred to a separate sterile tube and the symbiont dinoflagellate fraction was washed of residual host material by another resuspension and centrifugation using the same buffer solution. Both host and symbiont fractions were flash-frozen, freeze-dried, and stored at -80°C as before.

Lipid extraction

Lipid extraction was performed according to the method stablished by Matyash *et al.* (2008) [1] with modifications. A total of 200 µL of ice-cold 10 mM sodium phosphate buffer (pH 7.4) containing deferoxamine mesylate (100 µM) was added to freeze-dried biomass samples Eppendorf tubes with 600 µL of ice-cold methanol and 100 µL of each internal standard mix (**Table S11**) were mixed by vortex for 1 min. Methyl tert-butyl ether (2 mL) was added to the mixture and thoroughly vortexed for 30 s, and transferred into an ice-bath sonicator for 15 min. Samples were then incubated for 12 h at 20°C on a ThermoMixer shaking platform under constant mixing at 1000 rpm. Next, 600 µL of ice-cold MilliQ water was added and vortexed for 30 s. Tubes were centrifuged at 10,000x *g* for 10 min. at 4°C. The upper organic phase containing total lipid extracts were transferred to new clean glass vials (2 mL) and dried under N_2 gas. Dried extracts were resuspended in 100 µL of isopropanol (IPA) and transferred into 200 µL vial inserts and stored at -80°C until analysis.

Lipidomic analysis method development

Lipid extracts were analyzed by a Waters Acquity H-Class UPLC system interfaced with a Xevo G2-XS-QTOF Quadrupole Mass Spectrometer. A Waters Acquity Premier CSH C18 column (1.7 µm, 2.1 mm i.d. x 150 mm) with Acquity Premier CSH C18 1.7 µm VanGuard pre-column was used for separation with a flow rate of 0.2 mL min^{-1}, and column oven temperature set to 35°C. The autosampler sample chamber was maintained at 5°C and injection volume was set to

1.0 μL. IPA was used as the needle purge and wash solvents with pre-injection and post-injection washes of 3 s. For reversed-phase LC, mobile phase A consisted of water/acetonitrile (60:40 vol/vol.) and mobile phase B consisted of IPA/acetonitrile/water (88:10:2 vol./vol./vol.). Ammonium acetate was added at a final concentration of 10 mM into both mobile phases for runs performed in both positive and negative ionization modes. Lipids were separated by a 30 min linear gradient as follows: hold at 40% B for 30 s (starting conditions), from 40 to 100% B over 14.5 min., hold at 100% B from 15-18 min., decrease from 100 to 40% B from 18-19.5 min. and hold at 40% B from 19.5-30 min.

Preliminary data for lipid molecular species identification and quantification was obtained MS^E Data Independent Acquisition (DIA) mode, which resulted in higher sensitivity, but hampered annotation due to the fragmentation spectra of co-eluting lipids being combined in the high energy fragmentation channel at a given time point and was poorly reproducible (**Fig. S6**). Consequently, we obtained final data for lipid molecular species identification and quantification using SONAR-MS^E Data Independent Acquisition (DIA) mode as this allowed deconvolution of fragmentation spectra for co-eluting lipids where the ions were greater than 20 Da apart. ES capillary was set at 2 kV and sampling cone at 40 kV with 80 kV of source offset. Additional parameters included cone gas and desolvation gas flows set at 50 and 600 L/h, respectively. Source and desolvation temperatures were set at 120 and 250°C, respectively. SONAR quadrupole and MS^E acquisition settings were ionization mode specific (see below), with continuum acquisition of 1 scan sec^{-1}. The collision cell energy was consistently set at 40 eV. Lock spray was set to maintain mass accuracy in data acquisition using reference compound LueEnk (554.2615 *m*/*z* in negative mode and 556.2771 *m*/*z* in positive mode).

SONAR Quadrupole and MS^E settings

Negative mode
SONAR quadrupole scan range was split into two mass-to-charge ratio (*m*/*z*) windows; 150 to 600 *m*/*z* and 550 to1150 *m*/*z*. Quadrupole peak width set at 20 *m*/*z*. MS^E acquisition had low and high masses set at 50 and 600 *m*/*z*, and 500 to 1100 *m*/*z* respectively.

Positive mode
SONAR quadrupole scan range was set to 400-1000 *m*/*z* with quadrupole peak width set to 20 *m*/*z*. MS^E acquisition had low and high masses set to 50 and 1150 *m*/*z*, respectively.

Compound characterization
A curation of lipids previously reported for Symbiodiniaceae and multiple species of cnidarians [2–6] had their molecular structures individually generated by Chemdraw, and they were saved as .mol files and imported into UNIFI software for the creation of an in-house library. Putative *in silico* fragmentation patterns were generated for each precursor in each ionization mode, and exact masses of specific fragments and/or neutral losses for each precursor were also incorporated to scanning parameters for each ionization mode. For library optimization, manual annotation of the 100 most abundant compounds present in each sample in each ionization mode was used to confirm the presence of previously reported lipid species and to add new ones to the final library (**Table S12**). Annotations were based on high energy spectra fragments and the precursors exact masses (**Fig. S7** and **Fig. S8**). Trial samples (data not shown) were analyzed prior to the full study for individual compound confirmation and determination of retention times.

Lipidomic data processing

LC-MS instrument control and data acquisition was carried out using Waters MassLynx, with LC-MS data subsequently imported into UNIFI for post-acquisition processing. Lipids were identified using the in-house manufactured library described above. Precursors ions appeared in low energy mode and their respective specific fragments were obtained in high energy mode. A maximum error of 10 ppm was defined for the attribution of the precursor ions, with a variation of 0.6 min. tolerance set for retention time identification. In each batch, 0.3 min and 0.2 ms. were set as retention time and SONAR drift time tolerances, respectively, for comparison among samples. Isomers with the same *m*/*z* that presented retention times differences below 0.3 min. had specific fragments appearing in the same channel and therefore could not be separated and were reported as isomers in the data summary. Pigments and triacylglycerol (TAG) were analyzed in positive mode, whereas betaine lipids and glycolipids were identified in positive mode but quantified in negative mode following Botana *et al.* (2022) [6]. All other membrane lipids and free fatty acids (FFA) were analyzed in negative mode.

The summary of all adducts and isotope peak areas was used for quantification of each lipid molecular species. Each peak integration was manually inspected for correct peak detection and accurate area determination. The area ratio of each lipid was calculated by dividing the peak area of each lipid by the corresponding internal standard area, or by using the external calibration curve (*i.e.,* for glycolipids). The concentration of each lipid species was estimated by multiplying the area ratio by the concentration of the corresponding internal standard (IS) (**Table S12**). Glycolipids had external calibration curves relative to 1,2-diheptadecanoyl-sn-glycero-3-phosphocholine PC (17:0/17:0) IS and their concentration was estimated by multiplying the area ratio by the calculated correction response factor (**Table S13**). Glycolipid standards were injected separately generating dilution curves with seven points each, based on concentration ranges varying from 1.562-25 ng μL^{-1} in positive mode and 1.562-100 ng μL^{-1} in negative mode, based on MS lower and upper detection limits, in which instrument linearity (R^2>0.99) was maintained. For all dilution curves, each point had half the concentration of the previous point and correction factors were calculated as slopes from graph curves of each external standard divided by PC (17:0/17:0) IS. No external calibration was performed for betaine lipids and pigments. Betaine lipids, chlorophyl-*a* had their peak areas divided by the peak area of PC (17:0/17:0) IS and other pigments had their peak areas divided by the peak area of lysoPC-17:0 IS, given their similarities in retention time, but lacked absolute concentrations and hence relative can only be compared among samples. Because the number of characterised lipid species was greater than the number of commercially available standards, only semi-quantification of analytes was plausible. The total amount of each molecular species was expressed in $\mu g\ g^{-1}$ of tissue/cell dry biomass.

Method validation

Data reproducibility was checked by calculation of coefficients of variation (CV) for each lipid species, and internal standards from pooled samples (N = 9) in both positive and negative ionization modes, injected before and after biological samples (**Table S14**). Compounds that presented CV values ≥30% in one or more analysis batch were discarded from the analysis method. For stability checking, additional standard curves (N = 3) were measured in each ionization mode. Instrument linearity (R^2 > 0.9) was maintained in the range of 1.562-25 ng/µL in positive mode and 1.562-100 ng/µL in negative mode (**Table S15** and **Fig. S9**).

M2. Characterization and quantification of eicosanoids and docosanoids using LC-MS

10 µL of HPLC grade water and 10 µL of IS mix were added into samples vials after they were analysed in the SFC method. Eicosanoids and docosanoids were quantified according to Kolmert *et al.* (2018) [7]. Lipid extracts were analysed using LC-MS/MS on a Waters UPLC Acquity coupled to a Xevo TQ-XS mass spectrometer system using a BEH C18 column (2.1 mm × 150 mm, 1.7 µm, Waters, Milford, MA) set at 60 °C and the injection volume was set to 7.5 µL using a 10 µL sample loop and a partial loop with needle overfill injection mode. Mobile phase A consisted of water with 0.1% of acetic acid and B of acetonitrile/isopropanol 90:10, v/v at a flow rate of 0.5 mL min^{-1}. Gradient elution was performed with 80% of A as the starting condition, linearly decreased to 65% at 2.5 min, 60% at 4.5 min, 58% at 6 min, 50% at 8 min, 35% at 14 min, 27.5% at 15.5 min, and 0% at 16.6 min. The chromatographic system MS source was only operated in negative-ion ESI mode, with the capillary voltage set to 2.40 kV, the desolvation gas flow to 1000 L h^{-1}, the cone gas flow to 150 L h^{-1}, and the desolvation temperature to 600 °C. The cone voltage and collision energy were individually optimized for each analyte. For calibration, an 11-point linear calibration model (range, 0.002–834 ng mL^{-1}) was used, applying a 1/x weighted least-squared regression. On the day of analysis, 10 µL of the ISs working solution and 10 µL of Milli-Q water were added to 50 µL of each calibration aliquot, resulting in a final solvent composition of 6:1 v/v methanol/water. The lower limit of quantification (LLOQ) was defined as the lowest calibration level having a signal-to-noise ratio (S/N) > 5 and a measured concentration ≤ 20% of the nominal. Before data acquisition, a system suitability test was performed to ascertain instrument performance. MassLynx was used for data processing and normalization, as for processing the octadecanoids dataset. Only regioisomers were separated using this method and no stereochemistry information was obtained. The platform allowed for total screened of 75 eicosanoids and 12 docosanoids (**Table S2**).

M3. *De novo* transcriptome assembly and mRNA abundance

Alternatively spiced transcriptomes of multiple species of Anthozoa and Symbiodiniaceae mRNA encoding Pfam-homologous proteins were assembled *de novo* (without. gtf/.gff gene annotation guidance) from previously sequenced mRNA-enriched RNAseq libraries (**Table S3**) using Fastp, STAR, StringTie, and TransDecoder programs as we previously published in Botana et al (2025) [8]. Using Fastp, the raw paired-end RNA sequence files were trimmed and filtered to remove sequencing adaptors and bases with Phred quality scores below 30. Reads with more than 3010% low-quality bases, containing more than 5 N bases, and reads shorter than 50 bases after trimming were discarded.

Un-mapped filtered RNA reads of each evaluated species were collected and mapped without guidance to their respective genomes using STAR (v2.7.10b). Splice-junction aware alignment outputs were assembled into sample transcriptomes with a minimum transcript coverage of two mapped reads *per* transcript base using StringTie2 (v2.1.7) v2.0.395. Sample transcriptomes were then merged with StringTie into species RNA transcriptomes with a minimum transcript expression >1 TPM in at least one sample pair. All assembled RNA transcript open reading frames (ORFs) greater than 100 amino acids were predicted using TransDecoder (TransDecoder.Predict v5.7.1) to predict the most likely protein coding sequences (CDS regions) and reduce the ORF false discovery rate. The ORFs candidates (≥100 amino acids) were then filtered to retain only

transcripts with Pfam domain homology to any described or hypothetical protein in a collection of 8 species of reference genomes (**Table S4**) using HMMER (hmmscan v3.4) using cutoff of e-value $=1e^{-5}$. Internal unique transcripts were used as training data for the species-specific Markov Model-derived coding potential of the ORFs and to revise if start codon choice was statistically appropriate. After prediction, the best ORF candidates with revised starts were propagated to each genome and only transcripts with valid ORF orientations were written into new genomic protein-coding transcript annotations.

To quantify gene expression, mRNA transcript abundance, and test for differential expression, gene and transcript FPKM/TPM were estimated by StringTie and un-normalised transcript read counts summarised at the gene (meta-feature) level. Homologous protein-coding transcript annotations were performed on the RNA sequence STAR genomic alignments for each sample, using FeatureCounts [9] (Rsubread v2.16.1). All counted genes were prepped (prepDE.py) and tested for differential expression among experimental conditions from the original raw datasets using DeSeq2 [10], with default internal gene-filtering procedures (see **Table S3** for RNA-seq libraries used for and gene expression counts estimates of all investigated enzymes). Before expression testing, counted genes transcripts were pre-filtered to retain only genes with at least 10 15 un-normalised reads counted in every sample.

Figures

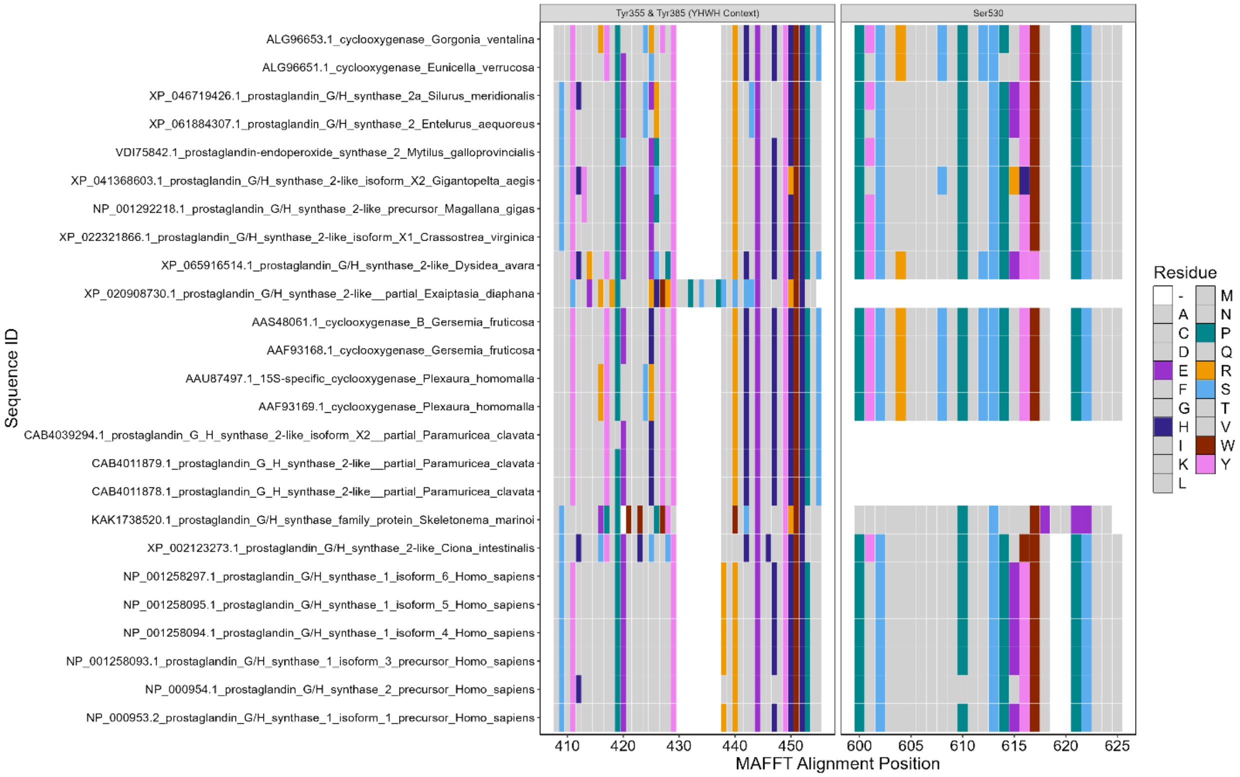

Figure S1. Sequence alignment of putative Prostaglandin G/H synthases (PTGS)/ Cyclooxygenases (COX) against the human COX-1 (NP_000953.2). *Exaiptasia diaphana* and *Skeletonema marinoi* confirmed their dioxygenase character by presenting the catalytic Tyr385, but both lacked further confirmation of Tyr355 and Ser530 motifs.

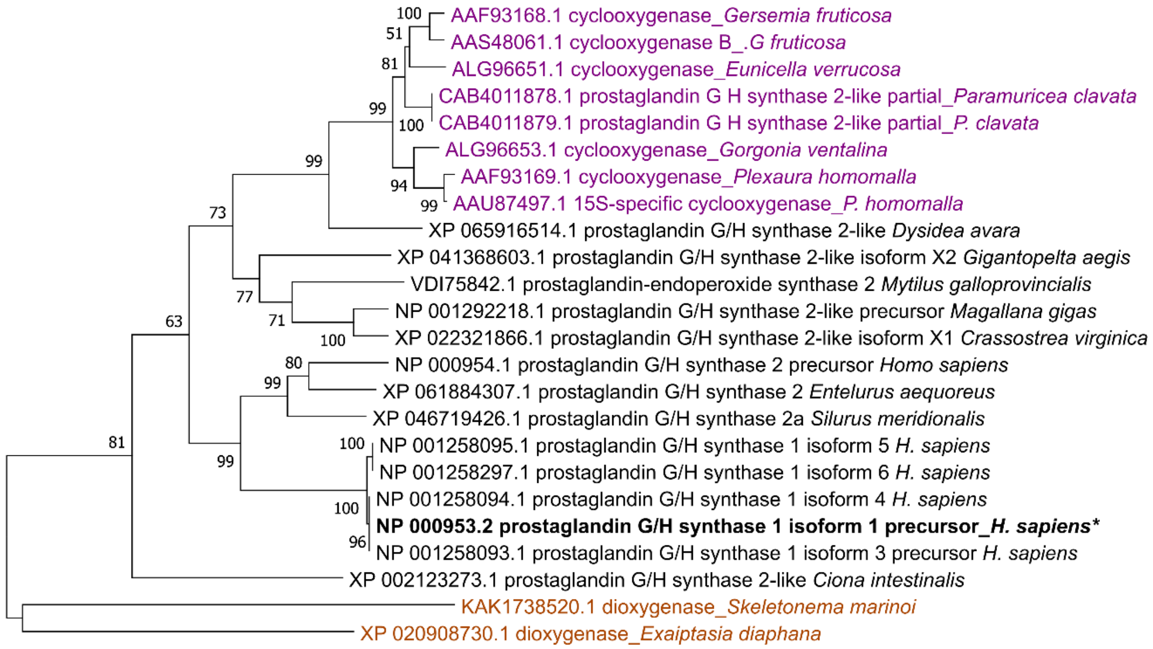

Figure S2. Phylogenetic tree of Prostaglandin G/H synthases (PTGS)/ Cyclooxygenases (COX) from different kingdoms, highlighting sequences for Anthozoa (represented in purple) based on protein sequence homology. Access numbers for sequences from publicly available domains are represented in the tree beside species names and listed in **Table S4**. Branch length represents predicted evolutionary distance between enzymes and numbers at nodes represent bootstrap support values, indicating the reliability of each branch separation. Human COX-1 (NP_000953.2) was used as a reference and is presented in bold. Sequences for *Exaiptasia diaphana* and *Skeletonema marinoi* were re-classified as dioxygenases according to Zámocký et al. (2015)[11].

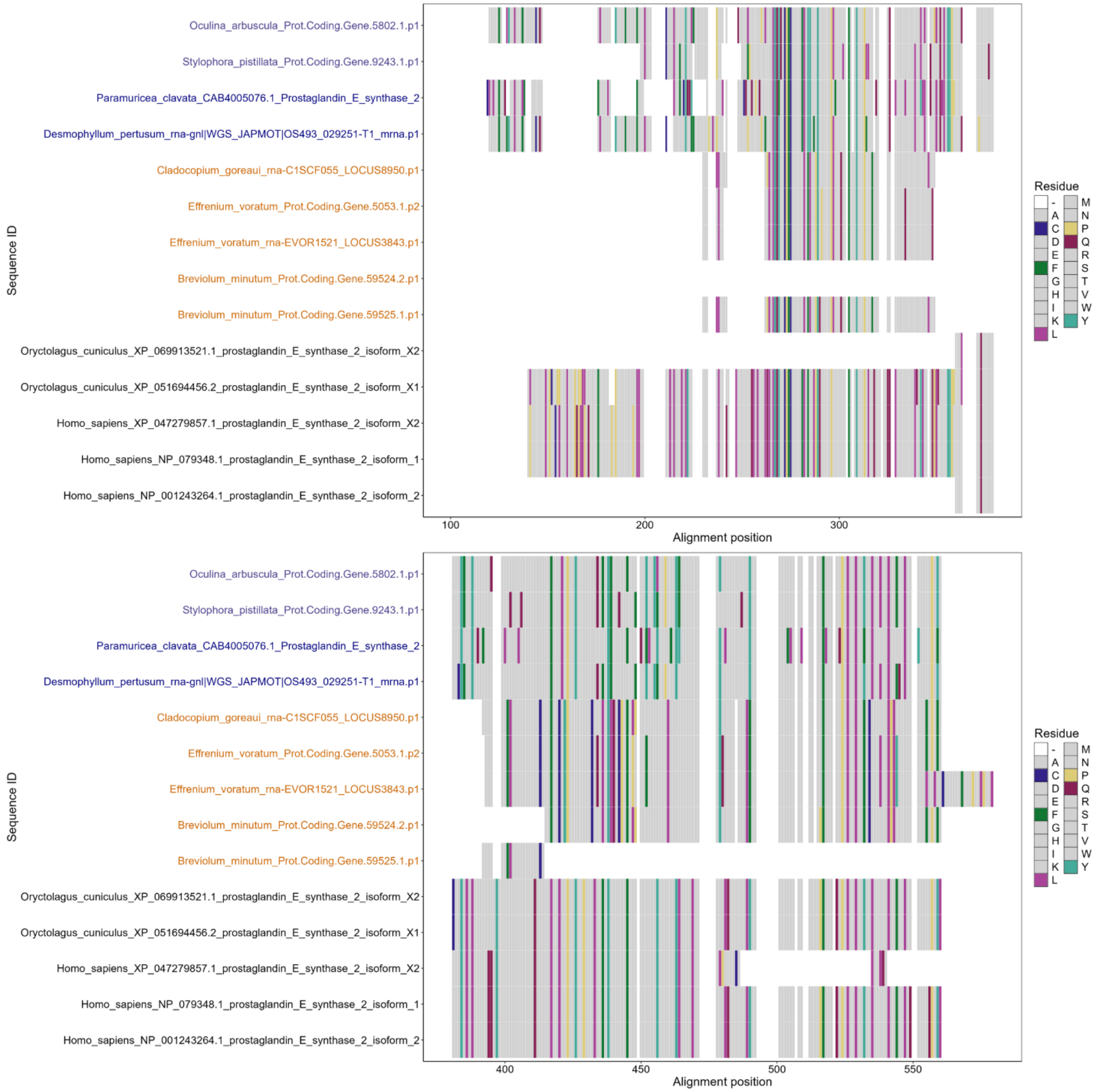

Figure S3. Sequence alignment of new prostaglandin E-synthase (PTGES) enzymes in cnidarians and Symbiodiniaceae. Amino acid alignment was performed against other species in the tree of life and only conserved motifs were colored coded to facilitate visualization. All sequences presented conserved LYQY and CTFC pair of motifs towards the N-terminus, with the human PTGES isoforms, followed by KW**W*D motif in the middle, and a F*GG motif towards the C-terminus. First 350 amino acids and the remaining ones are presented in upper and lower panels, respectively.

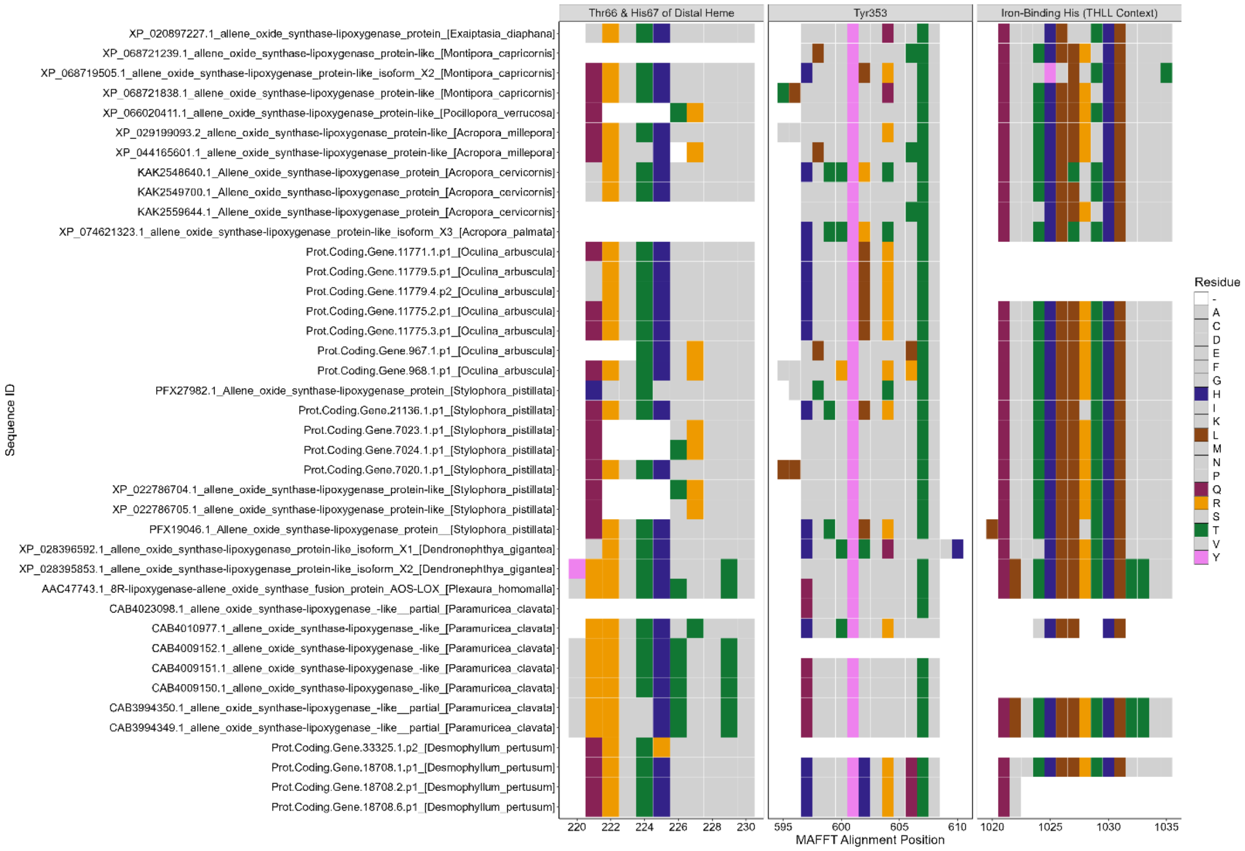

Figure S4. Sequence alignment of putative lipoxygenases (LOX) and allene-oxidase-LOX (AOS-LOX) enzymes of multiple cnidarians species against the *Plexura homomalla* AOS-LOX fusion (AAC47743.1). Classification of sequences was based on diagnostic motifs according to Koljak et al. (1997), Oldham et al. (2004), and Mashhadi et al. (2016) [12,13,15] and are further described in **Table S7**.

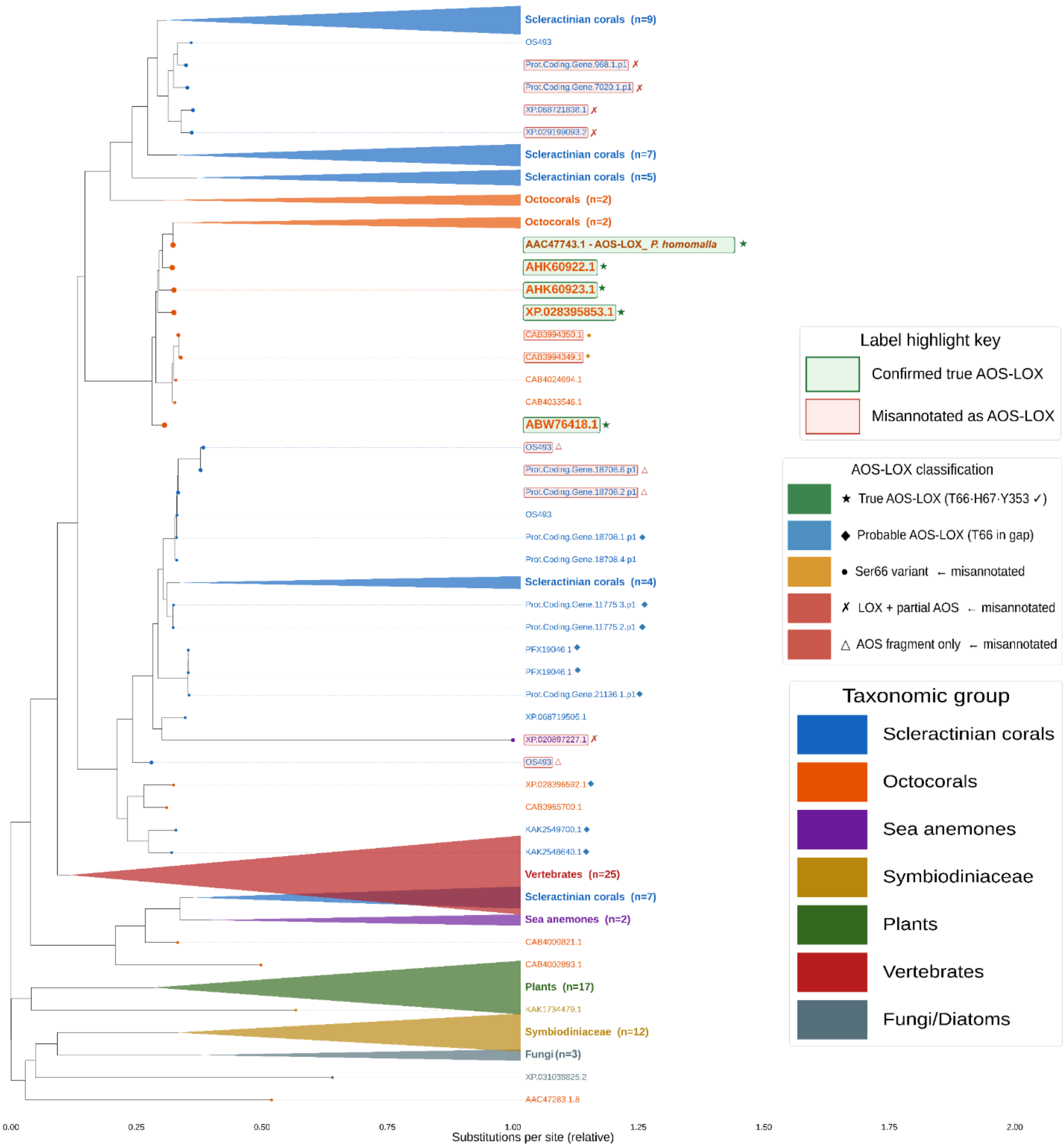

Figure S5. Phylogenetic tree of Lipoxygenases (LOXes) from different kingdoms, including novel sequences for Anthozoa and Symbiodiniaceae based on protein sequence homology. Access numbers for sequences from public domains are represented in the tree and detailed on **Table S3**. Enzyme sequence classification was based on criteria presented in **Table S7**. Branch length represents predicted evolutionary distance between enzymes.

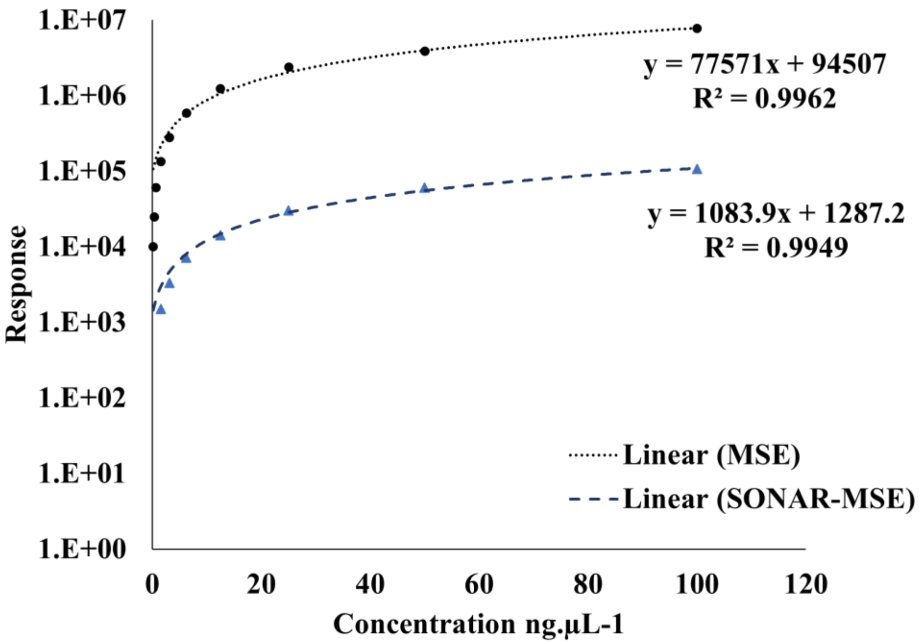

Figure S6. Sensitivity comparison between MS^E and SONAR-MS^E for the internal standard dihydroxyoctadec-4-en-2-yl-heptadecanamide (Cer (d18:1/17:0)) in negative ionization mode plotted in logarithmic scale. Concentration range showed no saturated peaks between 0.156-100 ng.μL^{-1} (ten concentration points) for MS^E and between 1.562-100 ng.μL^{-1} (seven concentration points) for SONAR-MS^E. For the latter, injections < 1.562 ng.μL^{-1} were below the detection limit. In both modes, instrument linearity was preserved along curve range, as represented by R^2 values.

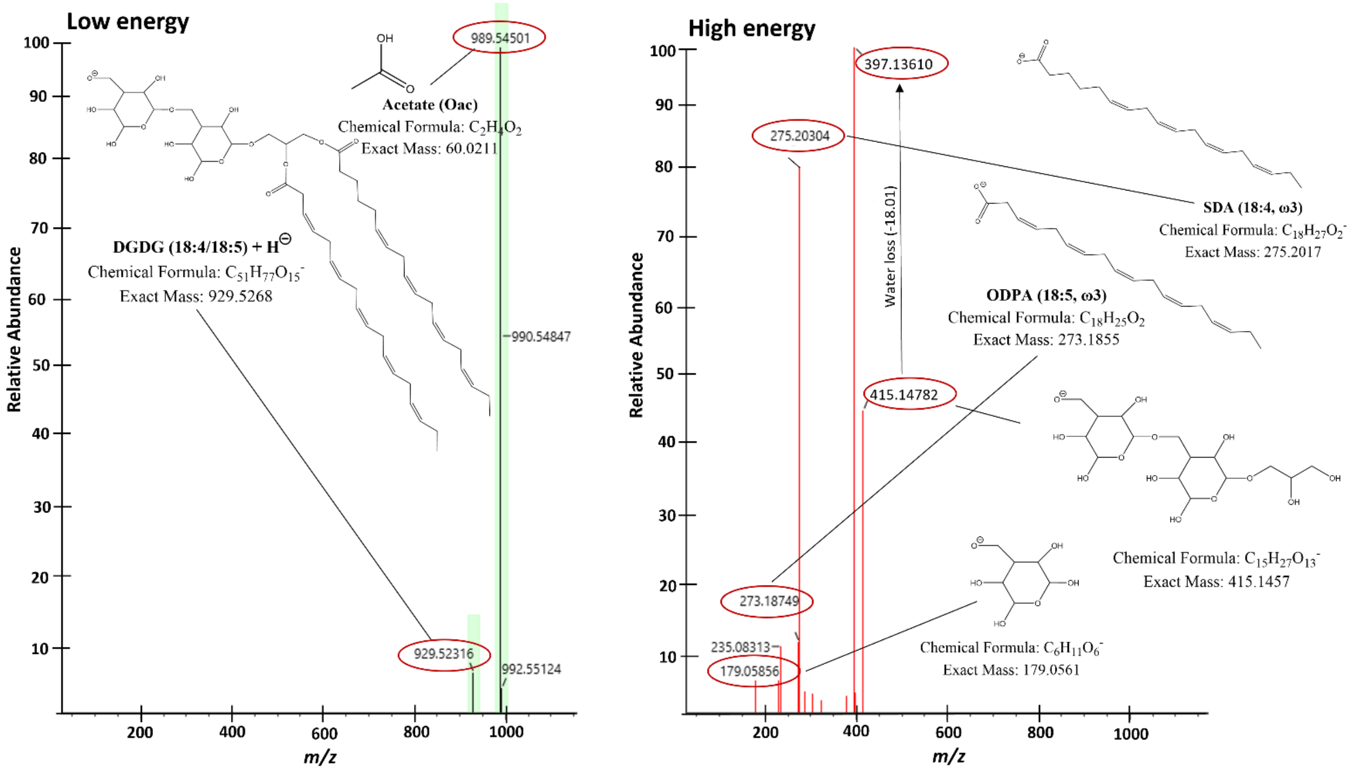

Figure S7. Structural characterization of the glycolipid digalactosyldiacylglycerol (DGDG) with stearidonic acid (SDA) and octadecapentaenoic acid (ODPA) acyl chains in negative ionization mode. Left: low energy mode in which only the precursors (ionized with $[H]^-$ and acetate adducts) appear. Right: high energy mode in which the characteristic fragments can be assigned for confirmation of compound identity.

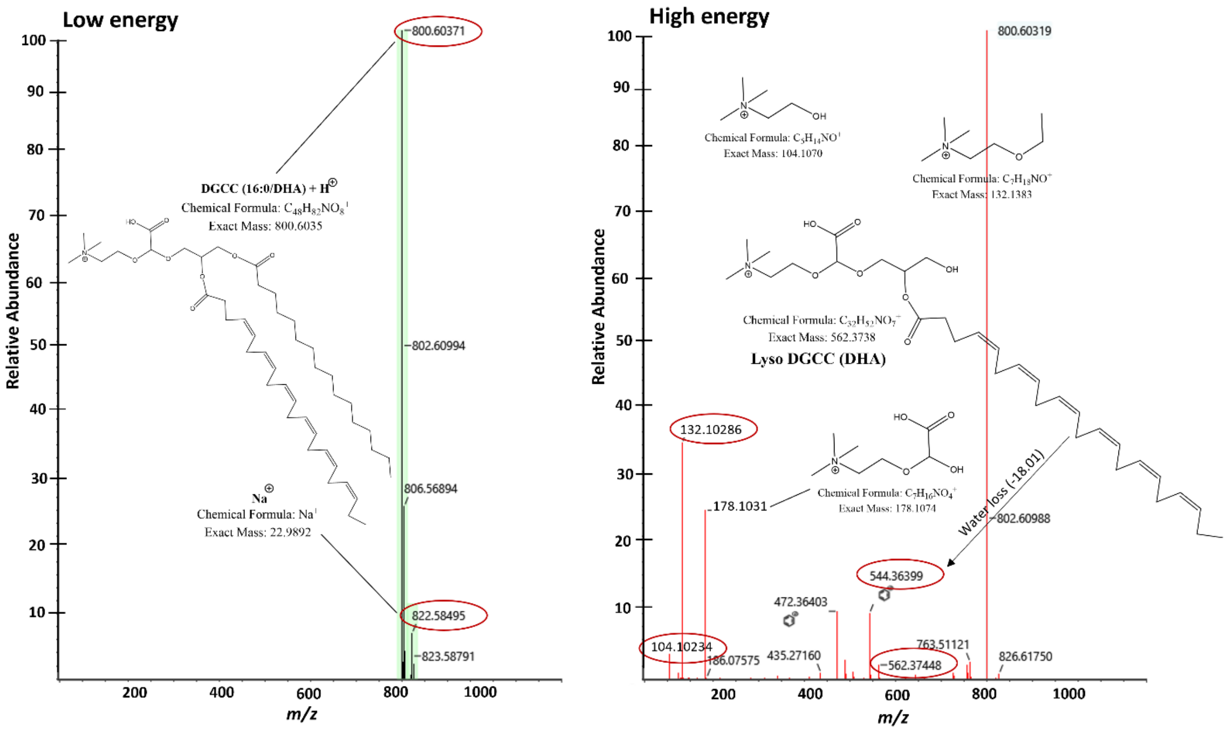

Figure S8. Structural characterization of the betaine lipid diacylglyceryl-3-O-carboxyhydroxymethylcholine (DGCC) with palmitic acid (16:0) and docosahexaenoic acid (DHA) fatty acids acyl chains in positive ionization mode. Left: low energy mode in which only the precursors (ionized with $[H]^+$ and $[Na]^+$ adducts) appear. Right: high energy mode in which the characteristic fragments can be assigned for confirmation of the compound identity. During fragmentation, the observed spectra peaks refer to the assigned fragments minus the loss of water (*i.e.,* -18.01 *m*/*z*).

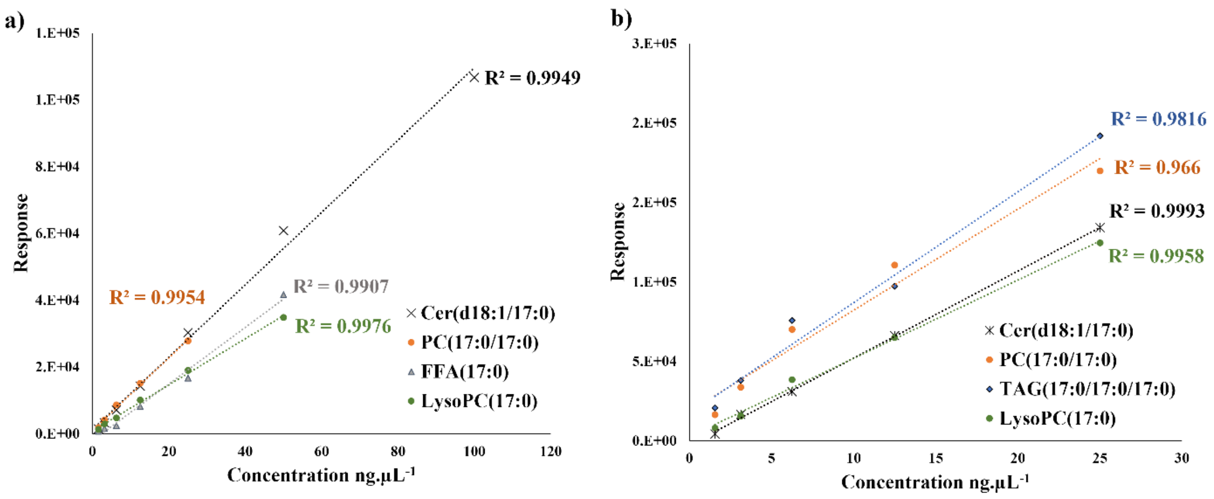

Figure S9. Instrument linearity check for each internal standard used as quality control with parameters described in **Table S13**. **a)** Negative ionization mode; **b)** Positive ionization mode.

Tables

Table S1. Species confirmation for *Breviolum minutum* in the Control and Treatment anemone samples. Genotyping was based on each replicate sample pool. *Complete table is in the Excel file in the electronic version.*

SAMPLE ID	Genetic identification
Culture of *B. minutum*	*Symbiodinium minutum* 18S rRNA gene, 5.8S rRNA gene, 28S rRNA gene, ITS1, and ITS2, strain CCMP830
Symbiont biomass from anemones symbiotic with *B. minutum* (Control)	*Symbiodinium minutum* 18S rRNA gene, 5.8S rRNA gene, 28S rRNA gene, ITS1, and ITS2, strain CCMP830
Symbiont biomass from anemones symbiotic with *B. minutum* (Treatment)	*Symbiodinium minutum* 18S rRNA gene, 5.8S rRNA gene, 28S rRNA gene, ITS1, and ITS2, strain CCMP830

Table S2: Detailed list of all screened compounds and their associated analytical platforms.

Lipid ID	Lipid class	Analytical platform	Observed in study
9-oxo-OTrE	Oxylipin octadecanoid	SFC	YES
13-oxo-OTrE	Oxylipin octadecanoid	SFC	YES
9-oxo-ODE	Oxylipin octadecanoid	SFC	YES
6(Z),9(Z)-13-oxo-ODE	Oxylipin octadecanoid	SFC	YES
9(Z),15(Z)-13-oxo-ODE	Oxylipin octadecanoid	SFC	YES
13-oxo-OME	Oxylipin octadecanoid	SFC	YES
10-oxo-ODA	Oxylipin octadecanoid	SFC	YES
13-oxo-ODA	Oxylipin octadecanoid	SFC	YES
trans-5,6-EpODE_1	Oxylipin octadecanoid	SFC	YES
trans-5,6-EpODE_2	Oxylipin octadecanoid	SFC	YES
cis-5,6-EpODE_1	Oxylipin octadecanoid	SFC	YES
cis-5,6-EpODE_2	Oxylipin octadecanoid	SFC	YES
trans-9,10-EpODA_1	Oxylipin octadecanoid	SFC	YES
trans-9,10-EpODA_2	Oxylipin octadecanoid	SFC	YES
cis-9S,10R-EpODA	Oxylipin octadecanoid	SFC	YES
cis-9R,10S-EpODA	Oxylipin octadecanoid	SFC	YES
cis-9S,10R-EpODE	Oxylipin octadecanoid	SFC	YES
cis-9R,10S-EpODE	Oxylipin octadecanoid	SFC	YES
cis-12,13-EpODE	Oxylipin octadecanoid	SFC	YES
cis-15,16-EpODE	Oxylipin octadecanoid	SFC	YES
trans-9,10-EpOME_1	Oxylipin octadecanoid	SFC	YES
trans-9,10-EpOME_2	Oxylipin octadecanoid	SFC	YES
cis-9S,10R-EpOME	Oxylipin octadecanoid	SFC	YES
cis-9R,10S-EpOME	Oxylipin octadecanoid	SFC	YES
trans-12,13-EpOME	Oxylipin octadecanoid	SFC	YES
cis-12(R),13(S)-EpOME	Oxylipin octadecanoid	SFC	YES
cis-12(S),13(R)-EpOME	Oxylipin octadecanoid	SFC	YES
9(R)-HOPE	Oxylipin octadecanoid	SFC	YES
9(S)-HOPE	Oxylipin octadecanoid	SFC	YES
10(R)-HOPE	Oxylipin octadecanoid	SFC	YES
10(S)-HOPE	Oxylipin octadecanoid	SFC	YES
13(R)-HOPE	Oxylipin octadecanoid	SFC	YES
13(S)-HOPE	Oxylipin octadecanoid	SFC	YES
15-HOPE	Oxylipin octadecanoid	SFC	NO
12(R)-HOPE	Oxylipin octadecanoid	SFC	YES
12(S)-HOPE	Oxylipin octadecanoid	SFC	YES
9(R)-HOTE	Oxylipin octadecanoid	SFC	YES
9(S)-HOTE	Oxylipin octadecanoid	SFC	YES

10(R)-HOTE	Oxylipin octadecanoid	SFC	YES
10(S)-HOTE	Oxylipin octadecanoid	SFC	YES
12(R)-HOTE	Oxylipin octadecanoid	SFC	YES
12(S)-HOTE	Oxylipin octadecanoid	SFC	YES
13(R)-HOTE	Oxylipin octadecanoid	SFC	YES
13(S)-HOTE	Oxylipin octadecanoid	SFC	YES
15(R,S)-HOTE	Oxylipin octadecanoid	SFC	YES
16(R)-HOTE	Oxylipin octadecanoid	SFC	YES
16(S)-HOTE	Oxylipin octadecanoid	SFC	YES
18-HOTE	Oxylipin octadecanoid	SFC	YES
5(E),9(Z),12(Z)-7(R,S)-HOTrE	Oxylipin octadecanoid	SFC	YES
6(Z),10(E),12(Z)-9(R)-HOTrE	Oxylipin octadecanoid	SFC	YES
6(Z),10(E),12(Z)-9(S)-HOTrE	Oxylipin octadecanoid	SFC	YES
6(Z),8(E),12(Z)-10(R)-HOTrE	Oxylipin octadecanoid	SFC	YES
6(Z),8(E),12(Z)-10(S)-HOTrE	Oxylipin octadecanoid	SFC	YES
6(Z),9(Z),13(E)-12(R,S)-HOTrE	Oxylipin octadecanoid	SFC	YES
6(Z),9(Z),11(E)-13(R)-HOTrE	Oxylipin octadecanoid	SFC	YES
6(Z),9(Z),11(E)-13(S)-HOTrE	Oxylipin octadecanoid	SFC	YES
6(Z),9(Z),12(Z)-18-HOTrE	Oxylipin octadecanoid	SFC	YES
9(R)-HOTrE	Oxylipin octadecanoid	SFC	YES
9(S)-HOTrE	Oxylipin octadecanoid	SFC	YES
10(R)-HOTrE	Oxylipin octadecanoid	SFC	YES
10(S)-HOTrE	Oxylipin octadecanoid	SFC	YES
12(R)-HOTrE	Oxylipin octadecanoid	SFC	YES
12(S)-HOTrE	Oxylipin octadecanoid	SFC	YES
13(R)-HOTrE	Oxylipin octadecanoid	SFC	YES
13(S)-HOTrE	Oxylipin octadecanoid	SFC	YES
15(R)-HOTrE	Oxylipin octadecanoid	SFC	YES
15(S)-HOTrE	Oxylipin octadecanoid	SFC	YES
16(R)-HOTrE	Oxylipin octadecanoid	SFC	YES
16(S)-HOTrE	Oxylipin octadecanoid	SFC	YES
17(R)-HOTrE	Oxylipin octadecanoid	SFC	YES
17(S)-HOTrE	Oxylipin octadecanoid	SFC	YES
18-HOTrE	Oxylipin octadecanoid	SFC	YES
6(Z),9(Z)-13(R)-HODE	Oxylipin octadecanoid	SFC	YES
6(Z),9(Z)-13(S)-HODE	Oxylipin octadecanoid	SFC	YES
9(Z),15(Z)-13(R)-HODE	Oxylipin octadecanoid	SFC	YES
9(Z),15(Z)-13(S)-HODE	Oxylipin octadecanoid	SFC	YES
9(R)-HODE	Oxylipin octadecanoid	SFC	YES
9(S)-HODE	Oxylipin octadecanoid	SFC	YES
8(E),12(Z)-10(R)-HODE	Oxylipin octadecanoid	SFC	YES

8(E),12(Z)-10(S)-HODE	Oxylipin octadecanoid	SFC	YES
9(Z),13(E)-12(R)-HODE	Oxylipin octadecanoid	SFC	YES
9(Z),13(E)-12(S)-HODE	Oxylipin octadecanoid	SFC	YES
13(R)-HODE	Oxylipin octadecanoid	SFC	YES
13(S)-HODE	Oxylipin octadecanoid	SFC	YES
9(Z),12(Z)-15(R,S)-HODE	Oxylipin octadecanoid	SFC	YES
9(Z),12(Z)-17(R,S)-HODE	Oxylipin octadecanoid	SFC	YES
9(Z),12(Z)-18-HODE	Oxylipin octadecanoid	SFC	YES
12(Z)-10-oxo-OME	Oxylipin octadecanoid	SFC	YES
11(E)-10-oxo-OME	Oxylipin octadecanoid	SFC	YES
12(Z),15(Z)-10-oxo-ODE	Oxylipin octadecanoid	SFC	YES
11(E),15(Z)-10-oxo-ODE	Oxylipin octadecanoid	SFC	YES
12(Z)-10(R)-HOME	Oxylipin octadecanoid	SFC	YES
12(Z)-10(S)-HOME	Oxylipin octadecanoid	SFC	YES
11(E)-10(R)-HOME	Oxylipin octadecanoid	SFC	YES
11(E)-10(S)-HOME	Oxylipin octadecanoid	SFC	YES
11(E),15(Z)-10(R)-HODE	Oxylipin octadecanoid	SFC	YES
12(Z),15(Z)-10(R)-HODE	Oxylipin octadecanoid	SFC	YES
11(E),15(Z)-10(S)-HODE	Oxylipin octadecanoid	SFC	YES
12(Z),15(Z)-10(S)-HODE	Oxylipin octadecanoid	SFC	YES
9(Z)-13(R)-HOME	Oxylipin octadecanoid	SFC	YES
9(Z)-13(S)-HOME	Oxylipin octadecanoid	SFC	YES
10(R)-HODA	Oxylipin octadecanoid	SFC	YES
10(S)-HODA	Oxylipin octadecanoid	SFC	YES
13(R)-HODA	Oxylipin octadecanoid	SFC	YES
13(S)-HODA	Oxylipin octadecanoid	SFC	YES
12,13-Epoxy-trans-9-KOME(EKODE)_1	Oxylipin octadecanoid	SFC	YES
12,13-Epoxy-trans-9-KOME(EKODE)_2	Oxylipin octadecanoid	SFC	YES
trans-9,10-Epoxy-11-HOME_1	Oxylipin octadecanoid	SFC	YES
trans-9,10-Epoxy-11-HOME_2	Oxylipin octadecanoid	SFC	YES
trans-9,10-Epoxy-11-HOME_3	Oxylipin octadecanoid	SFC	YES
trans-9,10-Epoxy-11-HOME_4	Oxylipin octadecanoid	SFC	YES
trans-9,10-Epoxy-13-HOME_1	Oxylipin octadecanoid	SFC	YES
trans-9,10-Epoxy-13-HOME_2	Oxylipin octadecanoid	SFC	YES
trans-9,10-Epoxy-13-HOME_3	Oxylipin octadecanoid	SFC	YES
trans-12,13-Epoxy-9-HOME_1	Oxylipin octadecanoid	SFC	YES
trans-12,13-Epoxy-9-HOME_2	Oxylipin octadecanoid	SFC	YES
trans-12,13-Epoxy-9-HOME_3	Oxylipin octadecanoid	SFC	YES
trans-12,13-Epoxy-11-HOME_1	Oxylipin octadecanoid	SFC	YES
trans-12,13-Epoxy-11-HOME_2	Oxylipin octadecanoid	SFC	YES

trans-12,13-Epoxy-11-HOME_3	Oxylipin octadecanoid	SFC	YES
trans-12,13-Epoxy-11-HOME_4	Oxylipin octadecanoid	SFC	YES
12-oxo-13-HOME	Oxylipin octadecanoid	SFC	YES
10(E),12(Z),14(E)-9R,16S-DiHOTrE	Oxylipin octadecanoid	SFC	NO
10(E),12(Z),14(E)-9R,16R-DiHOTrE	Oxylipin octadecanoid	SFC	NO
threo-9(R),10(R)-DiHODE	Oxylipin octadecanoid	SFC	YES
threo-9(S),10(S)-DiHODE	Oxylipin octadecanoid	SFC	YES
erythro-9,10-DiHODE	Oxylipin octadecanoid	SFC	YES
erythro-12,13-DiHODE	Oxylipin octadecanoid	SFC	YES
threo_1/_2/erythro_1-15,16-DiHODE	Oxylipin octadecanoid	SFC	YES
erythro-15,16-DiHODE_2	Oxylipin octadecanoid	SFC	YES
threo-9(R),10(R)-DiHOME	Oxylipin octadecanoid	SFC	YES
threo-9(S),10(S)-DiHOME	Oxylipin octadecanoid	SFC	YES
erythro-9,10-DiHOME_1	Oxylipin octadecanoid	SFC	YES
erythro-9,10-DiHOME_2	Oxylipin octadecanoid	SFC	YES
threo_1/_2/erythro_1-12,13-DiHOME	Oxylipin octadecanoid	SFC	YES
Erythro-12,13-DiHOME_2	Oxylipin octadecanoid	SFC	YES
threo-9,10-DiHODA_1	Oxylipin octadecanoid	SFC	YES
(threo_2/erythro_1)-9,10-DiHODA	Oxylipin octadecanoid	SFC	YES
erythro-9,10-DiHODA_2	Oxylipin octadecanoid	SFC	YES
9(S),10(S),13(S)-TriHODE	Oxylipin octadecanoid	SFC	YES
9(S),10(S),11(R)-TriHODE	Oxylipin octadecanoid	SFC	YES
9(S),12(S),13(S)-TriHODE	Oxylipin octadecanoid	SFC	YES
9(S),10(S),13(R)-TriHOME	Oxylipin octadecanoid	SFC	YES
9(R),10(S),13(R)-TriHOME	Oxylipin octadecanoid	SFC	YES
9(S),10(S),13(S)-TriHOME	Oxylipin octadecanoid	SFC	YES
9(R),10(S),13(S)-TriHOME	Oxylipin octadecanoid	SFC	YES
9(S),10(R),13(R)-TriHOME	Oxylipin octadecanoid	SFC	YES
9(S),10(R),13(S)-TriHOME	Oxylipin octadecanoid	SFC	YES
9(R),10(R),13(R)-TriHOME	Oxylipin octadecanoid	SFC	YES
9(R),10(R),13(S)-TriHOME	Oxylipin octadecanoid	SFC	YES
9(S),10(S),11(R)-TriHOME	Oxylipin octadecanoid	SFC	YES
11(R),12(S),13(S)-TriHOME	Oxylipin octadecanoid	SFC	YES
9(R),12(S),13(S)-TriHOME	Oxylipin octadecanoid	SFC	YES
9(S),12(S),13(R)-TriHOME	Oxylipin octadecanoid	SFC	YES
9,12,13-TriHOME	Oxylipin octadecanoid	SFC	YES
9(S),12(S),13(S)-TriHOME	Oxylipin octadecanoid	SFC	YES
9(S),12(R),13(R)-TriHOME	Oxylipin octadecanoid	SFC	YES
9(),12(),13()-TriHOME	Oxylipin octadecanoid	SFC	YES
19,20-DiHDPA	Oxylipin docosanoid	LC-GOM	YES
10,17-DiHDoHE	Oxylipin docosanoid	LC-GOM	YES

5,6-DiHETE	Oxylipin eicosanoid	LC-GOM	YES
5,15-DiHETE	Oxylipin eicosanoid	LC-GOM	YES
8,15-DiHETE	Oxylipin eicosanoid	LC-GOM	YES
14,15-DiHETE	Oxylipin eicosanoid	LC-GOM	YES
17,18-DiHETE	Oxylipin eicosanoid	LC-GOM	YES
5,6-DiHETrE	Oxylipin eicosanoid	LC-GOM	YES
8,9-DiHETrE	Oxylipin eicosanoid	LC-GOM	YES
11,12-DiHETrE	Oxylipin eicosanoid	LC-GOM	YES
14,15-DiHETrE	Oxylipin eicosanoid	LC-GOM	YES
16(17)-EpDPE	Oxylipin docosanoid	LC-GOM	YES
19(20)-EpDPE	Oxylipin docosanoid	LC-GOM	YES
14(15)-EpETE	Oxylipin eicosanoid	LC-GOM	YES
17(18)-EpETE	Oxylipin eicosanoid	LC-GOM	YES
5(6)-EpETrE	Oxylipin eicosanoid	LC-GOM	YES
8(9)-EpETrE	Oxylipin eicosanoid	LC-GOM	YES
11(12)-EpETrE	Oxylipin eicosanoid	LC-GOM	YES
14(15)-EpETrE	Oxylipin eicosanoid	LC-GOM	YES
4-HDoHE	Oxylipin docosanoid	LC-GOM	NO
8-HDoHE	Oxylipin docosanoid	LC-GOM	YES
11-HDoHE	Oxylipin docosanoid	LC-GOM	YES
14-HDoHE	Oxylipin docosanoid	LC-GOM	NO
17-HDoHE	Oxylipin docosanoid	LC-GOM	YES
11-HEDE	Oxylipin eicosanoid	LC-GOM	YES
15-HEDE	Oxylipin eicosanoid	LC-GOM	YES
5-HEPE	Oxylipin eicosanoid	LC-GOM	YES
8-HEPE	Oxylipin eicosanoid	LC-GOM	YES
9-HEPE	Oxylipin eicosanoid	LC-GOM	YES
11-HEPE	Oxylipin eicosanoid	LC-GOM	YES
12-HEPE	Oxylipin eicosanoid	LC-GOM	YES
15-HEPE	Oxylipin eicosanoid	LC-GOM	YES
18-HEPE	Oxylipin eicosanoid	LC-GOM	YES
5-HETE	Oxylipin eicosanoid	LC-GOM	YES
8-HETE	Oxylipin eicosanoid	LC-GOM	YES
9-HETE	Oxylipin eicosanoid	LC-GOM	YES
11-HETE	Oxylipin eicosanoid	LC-GOM	YES
12-HETE	Oxylipin eicosanoid	LC-GOM	YES
15-HETE	Oxylipin eicosanoid	LC-GOM	YES
19-HETE	Oxylipin eicosanoid	LC-GOM	NO
20-HETE	Oxylipin eicosanoid	LC-GOM	NO
5-HETrE	Oxylipin eicosanoid	LC-GOM	NO
8-HETrE	Oxylipin eicosanoid	LC-GOM	YES

15-HETrE	Oxylipin eicosanoid	LC-GOM	YES
12-HHTrE	Oxylipin eicosanoid	LC-GOM	YES
7,17-hydroxy-DPA	Oxylipin docosanoid	LC-GOM	NO
iPF2a-IV	Oxylipin eicosanoid	LC-GOM	NO
5-iPF2a-VI	Oxylipin eicosanoid	LC-GOM	NO
8,12-iPF2a-VI	Oxylipin eicosanoid	LC-GOM	NO
13,14-dihydro-15-keto-PGE2	Oxylipin eicosanoid	LC-GOM	YES
5-KETE	Oxylipin eicosanoid	LC-GOM	YES
12-KETE	Oxylipin eicosanoid	LC-GOM	YES
15-KEDE	Oxylipin eicosanoid	LC-GOM	NO
15-KETE	Oxylipin eicosanoid	LC-GOM	YES
LTB3	Oxylipin eicosanoid	LC-GOM	NO
LTB4	Oxylipin eicosanoid	LC-GOM	NO
6-trans-LTB4	Oxylipin eicosanoid	LC-GOM	NO
20-OH-LTB4	Oxylipin eicosanoid	LC-GOM	NO
20-COOH-LTB4	Oxylipin eicosanoid	LC-GOM	NO
LTE4	Oxylipin eicosanoid	LC-GOM	NO
14,15-LTE4	Oxylipin eicosanoid	LC-GOM	NO
LXA4_epiLXA4	Oxylipin eicosanoid	LC-GOM	NO
LXA5	Oxylipin eicosanoid	LC-GOM	NO
LXB4	Oxylipin eicosanoid	LC-GOM	NO
6-keto-PGF1a	Oxylipin eicosanoid	LC-GOM	NO
PGB2	Oxylipin eicosanoid	LC-GOM	YES
PGD1	Oxylipin eicosanoid	LC-GOM	YES
PGD2	Oxylipin eicosanoid	LC-GOM	YES
PGD3	Oxylipin eicosanoid	LC-GOM	YES
PGE1	Oxylipin eicosanoid	LC-GOM	YES
PGE2	Oxylipin eicosanoid	LC-GOM	YES
8-isoPGE2	Oxylipin eicosanoid	LC-GOM	NO
PGE3	Oxylipin eicosanoid	LC-GOM	YES
PGF2a	Oxylipin eicosanoid	LC-GOM	YES
8-iso-PGF2a	Oxylipin eicosanoid	LC-GOM	NO
8-iso-PGF3a	Oxylipin eicosanoid	LC-GOM	NO
11B-PGF2a	Oxylipin eicosanoid	LC-GOM	NO
15-deoxy-delta-12,14-PGJ2	Oxylipin eicosanoid	LC-GOM	YES
PGJ2anddelta-12-PGJ2	Oxylipin eicosanoid	LC-GOM	NO
RvD1	Oxylipin docosanoid	LC-GOM	NO
17-RvD1	Oxylipin docosanoid	LC-GOM	NO
RvD2	Oxylipin docosanoid	LC-GOM	NO
TXB1	Oxylipin eicosanoid	LC-GOM	YES
TXB2	Oxylipin eicosanoid	LC-GOM	YES

TXB2_P2	Oxylipin eicosanoid	LC-GOM	YES
11-keto-TXB2	Oxylipin eicosanoid	LC-GOM	YES
11-keto-TXB3	Oxylipin eicosanoid	LC-GOM	YES
TXB3	Oxylipin eicosanoid	LC-GOM	YES
CAEP(d:182/16:0)	Sphingolipid	LC-MS-SONAR	YES
Cer(d18:0/h16:1)	Sphingolipid	LC-MS-SONAR	YES
Cer(d18:0/h18:1)	Sphingolipid	LC-MS-SONAR	YES
Cer(t18:0/16:0)	Sphingolipid	LC-MS-SONAR	YES
Chlorophyll-a	Pigment	LC-MS-SONAR	YES
Chlorophyll-c2	Pigment	LC-MS-SONAR	YES
DGCC (16:0/18:3)	Betaine lipid	LC-MS-SONAR	YES
DGCC (16:0/DHA)	Betaine lipid	LC-MS-SONAR	YES
DGCC (16:0/DPA)	Betaine lipid	LC-MS-SONAR	YES
DGCC (18:0/28:7)	Betaine lipid	LC-MS-SONAR	YES
DGCC (18:0/DHA)	Betaine lipid	LC-MS-SONAR	YES
DGCC(DHA/DHA)	Betaine lipid	LC-MS-SONAR	YES
DGDG (18:4/18:4)	Glycolipid	LC-MS-SONAR	YES
DGDG (18:4/18:5)	Glycolipid	LC-MS-SONAR	YES
DGDG (18:4/DHA)	Glycolipid	LC-MS-SONAR	YES
FFA (14:0)	Free fatty acid (FFA)	LC-MS-SONAR	YES
FFA (16:0)	Free fatty acid (FFA)	LC-MS-SONAR	YES
FFA (18:1)	Free fatty acid (FFA)	LC-MS-SONAR	YES
FFA (18:2)	Free fatty acid (FFA)	LC-MS-SONAR	YES
FFA (18:3)	Free fatty acid (FFA)	LC-MS-SONAR	YES
FFA (18:4)	Free fatty acid (FFA)	LC-MS-SONAR	YES
FFA (18:5)	Free fatty acid (FFA)	LC-MS-SONAR	YES
FFA (20:3)	Free fatty acid (FFA)	LC-MS-SONAR	YES
FFA (ARA)	Free fatty acid (FFA)	LC-MS-SONAR	YES
FFA (DHA)	Free fatty acid (FFA)	LC-MS-SONAR	YES
FFA (DPA)	Free fatty acid (FFA)	LC-MS-SONAR	YES
FFA (EPA)	Free fatty acid (FFA)	LC-MS-SONAR	YES
LoPC(o16:0) - LPAF	Lysolipid	LC-MS-SONAR	YES
LPC (16:0)	Lysolipid	LC-MS-SONAR	YES
LPC(DHA)	Lysolipid	LC-MS-SONAR	YES
LysoDGCC(DHA)	Lysolipid	LC-MS-SONAR	YES
MGDG (18:2/18:3)	Glycolipid	LC-MS-SONAR	YES
MGDG (18:3/18:3)	Glycolipid	LC-MS-SONAR	YES
MGDG (18:4/18:4)	Glycolipid	LC-MS-SONAR	YES
MGDG (18:4/18:5)	Glycolipid	LC-MS-SONAR	YES
MGDG (18:4/DHA)	Glycolipid	LC-MS-SONAR	YES
oPC(o16:0/ARA)	Plasmalogen	LC-MS-SONAR	YES

oPC(o18:0/DHA)	Plasmalogen	LC-MS-SONAR	YES
oPC(o18:2/ARA)	Plasmalogen	LC-MS-SONAR	YES
oPC(o16:0/DHA)	Plasmalogen	LC-MS-SONAR	YES
oPE(o16:0/ARA)	Plasmalogen	LC-MS-SONAR	YES
oPE(o16:0/DPA)	Plasmalogen	LC-MS-SONAR	YES
oPE(o18:0/ARA)	Plasmalogen	LC-MS-SONAR	YES
PC (16:0/18:1)	Phospholipid-phosphatidylcholine	LC-MS-SONAR	YES
PC (16:0/DHA)	Phospholipid-phosphatidylcholine	LC-MS-SONAR	YES
PC (18:1/18:1)	Phospholipid-phosphatidylcholine	LC-MS-SONAR	YES
PC(DHA/DHA)	Phospholipid-phosphatidylcholine	LC-MS-SONAR	YES
PE (16:0/18:1)	Phospholipid-phosphatidylethanolamine	LC-MS-SONAR	YES
PE (18:0/22:4)	Phospholipid-phosphatidylethanolamine	LC-MS-SONAR	YES
PE (18:0/DPA)	Phospholipid-phosphatidylethanolamine	LC-MS-SONAR	YES
PE (18:1/18:1)	Phospholipid-phosphatidylethanolamine	LC-MS-SONAR	YES
PE (19:1/ARA)	Phospholipid-phosphatidylethanolamine	LC-MS-SONAR	YES
Peridinin	Pigment	LC-MS-SONAR	YES
Pheophytin-a	Pigment	LC-MS-SONAR	YES
pPE(p18:0/17:1)	Plasmalogen	LC-MS-SONAR	YES
pPE(p18:0/20:0)	Plasmalogen	LC-MS-SONAR	YES
pPE(p18:0/ARA)	Plasmalogen	LC-MS-SONAR	YES
pPE(p18:0/EPA)	Plasmalogen	LC-MS-SONAR	YES
TAG (12:0/16:0/18:1)	Triacylglycerol	LC-MS-SONAR	YES
TAG (14:0/14:0/16:1)	Triacylglycerol	LC-MS-SONAR	YES
TAG (14:0/14:0/DHA)	Triacylglycerol	LC-MS-SONAR	YES
TAG (14:0/16:0/17:1)	Triacylglycerol	LC-MS-SONAR	YES
TAG (14:0/16:0/18:0)	Triacylglycerol	LC-MS-SONAR	YES
TAG (14:0/16:0/18:1)	Triacylglycerol	LC-MS-SONAR	YES
TAG (14:0/16:0/18:2)	Triacylglycerol	LC-MS-SONAR	YES
TAG (14:0/16:0/DHA)	Triacylglycerol	LC-MS-SONAR	YES
TAG (14:0/18:1/18:1)	Triacylglycerol	LC-MS-SONAR	YES
TAG (14:0/18:1/18:3)	Triacylglycerol	LC-MS-SONAR	YES
TAG (14:0/18:1/22:1)	Triacylglycerol	LC-MS-SONAR	YES
TAG (14:1/16:1/18:2)	Triacylglycerol	LC-MS-SONAR	YES
TAG (15:0/16:0/18:1)	Triacylglycerol	LC-MS-SONAR	YES
TAG (16:0/16:0/16:0)	Triacylglycerol	LC-MS-SONAR	YES

TAG (16:0/16:0/16:1)	Triacylglycerol	LC-MS-SONAR	YES
TAG (16:0/16:0/17:1)	Triacylglycerol	LC-MS-SONAR	YES
TAG (16:0/16:0/18:0)	Triacylglycerol	LC-MS-SONAR	YES
TAG (16:0/16:0/18:1)	Triacylglycerol	LC-MS-SONAR	YES
TAG (16:0/16:0/18:2)	Triacylglycerol	LC-MS-SONAR	YES
TAG (16:0/16:0/18:3)	Triacylglycerol	LC-MS-SONAR	YES
TAG (16:0/16:0/DHA)	Triacylglycerol	LC-MS-SONAR	YES
TAG (16:0/16:0/DPA)	Triacylglycerol	LC-MS-SONAR	YES
TAG (16:0/16:1/16:1)	Triacylglycerol	LC-MS-SONAR	YES
TAG (16:0/16:1/17:1)	Triacylglycerol	LC-MS-SONAR	YES
TAG (16:0/16:1/18:1)	Triacylglycerol	LC-MS-SONAR	YES
TAG (16:0/16:1/DHA)	Triacylglycerol	LC-MS-SONAR	YES
TAG (16:0/17:0/18:0)	Triacylglycerol	LC-MS-SONAR	YES
TAG (16:0/18:0/18:1)	Triacylglycerol	LC-MS-SONAR	YES
TAG (16:0/18:0/DHA)	Triacylglycerol	LC-MS-SONAR	YES
TAG (16:0/18:1/18:2)	Triacylglycerol	LC-MS-SONAR	YES

Abbreviations: SFC: Supercritical-fluid chromatography; LC-GOM: Liquid Chromatography – General Oxylipin Method; LC-MS-SONAR: Liquid Chromatography-Mass Spectrometry in SONAR mode.

Table S3. Novel putative actively expressed enzyme sequences described for Anthozoa and Symbiodiniaceae. (**A**) Prostaglandin E-synthase (PTGES) enzymes in cnidarians and Symbiodiniaceae, (**B**) Lipoxygenases (LOXes) and Allene-oxidase-LOX (AOS-LOX) in cnidarians and (**C**) detailed access numbers for all enzymes in the LOX phylogeny tree.
See Excel file and .FASTA and .txt files in electronic version.

Table S4. References for genomes and RNAseq libraries used for *de-novo* transcriptome assenbly of Anthozoa and Symbiodiniaceae species.

	Species	Genome reference	RNAseq datasets
**Anthozoa**	*Desmophyllum pertusum*	GCA_029204205.1	SRR16705990- SRR16705992
	Oculina arbuscula	GCA_964656845.1	ERR13999140, ERR13999143
	Stylophora pistillata	GCA_964205215.1	SRR12011939-SRR12011941, SRR12011944-SRR12011946
	Nematostella vectensis	GCF_932526225.1	SRR24348396, SRR24348400, SRR24348404
	Calliactis parasitica	GCA_964340385.1	ERR14707636
**Symbiodiniaceae**	*Breviolum minutum*	GCA_965643015.1	SRR9128698-SRR9128703
	Effrenium voratum	GCA_963377175.1	ERR13925966, DRR658383
	Cladocopium goreaui	GCA_947184155.2	SRR14297378, SRR14297386, SRR14297387

Table S5: Full names and abbreviations of oxylipins monitored in the current study.

**Octadecanoids**		
**Compound**	**Parent PUFA**	**Abbreviation**
trans-9,10-epoxy-octadecanoic acid	OA	*trans*-9(10)-EpODA
cis-9,10-epoxy-octadecanoic acid	OA	*cis*-9(10)-EpODA
threo-9,10-dihydroxy-octadecanoic acid	OA	*threo*-9,10-DiHODA
erythro-9,10-dihydroxy-octadecanoic acid	OA	*erythro*-9,10-DiHODA
10(*S*)-hydroxy-8(*E*)-octadecenoic acid	OA	8(*E*)-10(*S*)-HOME
7(*S*),10(*S*)-dihydroxy-octadecenoic acid	OA	7(*S*),10(*S*)-DiHOME
trans-5,6-epoxy-octadecadienoic acid	CA	*trans*-5(6)-EpODE
erythro-5,6-dihydroxy-octadecadienoic acid	CA	*erythro*-5,6-DiHODE
cis-5,6-epoxy-octadecadienoic acid	PA	*cis*-5(6)-EpODE
trans-9,10-epoxy-octadecenoic acid	LA	*trans*-9(10)-EpOME
cis-9,10-epoxy-octadecenoic acid	LA	*cis*-9(10)-EpOME
trans-12,13-epoxy-octadecenoic acid	LA	*trans*-12(13)-EpOME
cis-12,13-epoxy-octadecenoic acid	LA	*cis*-12(13)-EpOME
9-oxo-octadecadienoic acid	LA	9-oxo-ODE
13-oxo-octadecadienoic acid	LA	13-oxo-ODE
10-oxo-11(*E*)-octadecenoic acid	LA	11(E)-10-oxo-OME
10-oxo-12(*Z*)-octadecenoic acid	LA	12(Z)-10-oxo-OME
13-oxo-9(*Z*)-octadecenoic acid	LA	9(Z)-13-oxo-OME
10-oxo-octadecanoic acid	LA	10-oxo-ODA
13-oxo-octadecanoic acid	LA	13-oxo-ODA
13-hydroxy-octadecadienoic acid	LA	13-HODE
9-hydroxy-octadecadienoic acid	LA	9-HODE
10-hydroxy-octadecadienoic acid	LA	10-HODE
12-hydroxy-octadecadienoic acid	LA	12-HODE
15-hydroxy-octadecadienoic acid	LA	15-HODE
17-hydroxy-octadecadienoic acid	LA	17-HODE
18-hydroxy-octadecadienoic acid	LA	18-HODE
10-hydroxy-12(*Z*)-octadecenoic acid	LA	12(*Z*)-10-HOME
10-hydroxy-11(*E*)-octadecenoic acid	LA	11(*E*)-10-HOME
13-hydroxy-9(*Z*)-octadecenoic acid	LA	9(*Z*)-13-HOME
10-hydroxy-octadecanoic acid	LA	10-HODA
13-hydroxy-octadecanoic acid	LA	13-HODA
12-oxo-13-hydroxy-octadecenoic acid	LA	12-oxo-13-HOME
9-hydroxy-*trans*-12,13-epoxy-octadecenoic acid	LA	9-OH-*trans*-12(13)-EpOME
11-hydroxy-*trans*-12,13-epoxy-octadecenoic acid	LA	11-OH-*trans*-12(13)-EpOME
11-hydroxy-*trans*-9,10-epoxy-octadecenoic acid	LA	11-OH-*trans*-9(10)-EpOME
13-hydroxy-*trans*-9,10-epoxy-octadecenoic acid	LA	13-OH-*trans*-9(10)-EpOME
9-oxo-*trans*-12,13-epoxy-octadecenoic acid	LA	EKODE
8(*R*),11(*S*)-dihydroxyoctadecadienoic acid	LA	8(*R*),11(*S*)-DiHODE

threo-9,10-dihydroxy-octadecenoic acid	LA	*threo*-9,10-DiHOME
erythro-9,10-dihydroxy-octadecenoic acid	LA	*erythro*-9,10-DiHOME
threo-12,13-dihydroxy-octadecenoic acid	LA	*threo*-12,13-DiHOME
erythro-12,13-dihydroxy-octadecenoic acid	LA	*erythro*-12,13-DiHOME
10,13-dihydroxy-octadecanoic acid	LA	10,13-DiHODA
9,10,13-trihydroxy-octadecenoic acid	LA	9,10,13-TriHOME
11(*R*),12(*S*),13(*S*)-trihydroxy-octadecenoic acid	LA	11(*R*),12(*S*),13(*S*)-TriHOME
9(*S*),10(*S*),11(*R*)-trihydroxy-octadecenoic acid	LA	9(*S*),10(*S*),11(*R*)-TriHOME
9,12,13-trihydroxy-octadecenoic acid	LA	9,12,13-TriHOME
cis-9,10-epoxy-octadecadienoic acid	ALA	*cis*-9(10)-EpODE
cis-12,13-epoxy-octadecadienoic acid	ALA	*cis*-12(13)-EpODE
cis-15,16-epoxy-octadecadienoic acid	ALA	*cis*-15(16)-EpODE
9-oxo-octadecatrienoic acid	ALA	9-oxo-OTrE
13-oxo-octadecatrienoic acid	ALA	13-oxo-OTrE
13-oxo-9(*Z*),15(*Z*)-octadecadienoic acid	ALA	13-oxo-ODE-α
10-oxo-12(*Z*),15(*Z*)-octadecadienoic acid	ALA	12(Z),15(Z)-10-oxo-ODE
10-oxo-11(*E*),15(*Z*)-octadecadienoic acid	ALA	11(E),15(Z)-10-oxo-ODE
10-oxo-15(*Z*)-octadecenoic acid	ALA	15(Z)-10-oxo-OME
9-hydroxy-octadecatrienoic acid	ALA	9-HOTrE
10-hydroxy-octadecatrienoic acid	ALA	10-HOTrE
12-hydroxy-octadecatrienoic acid	ALA	12-HOTrE
13-hydroxy-octadecatrienoic acid	ALA	13-HOTrE
15-hydroxy-octadecatrienoic acid	ALA	15-HOTrE
16-hydroxy-octadecatrienoic acid	ALA	16-HOTrE
17-hydroxy-octadecatrienoic acid	ALA	17-HOTrE
18-hydroxy-octadecatrienoic acid	ALA	18-HOTrE
10-hydroxy-11(*E*),15(*Z*)-octadecadienoic acid	ALA	11(*E*),15(*Z*)-10-HODE
10-hydroxy-12(*Z*),15(*Z*)-octadecadienoic acid	ALA	12(*Z*),15(*Z*)-10-HODE
13-hydroxy-9(*Z*),15(*Z*)-octadecadienoic acid	ALA	13-HODE-α
10-hydroxy-15(*Z*)-octadecenoic acid	ALA	15(*Z*)-10-HOME
9(*R*),16(*S*)-dihydroxy-octadecatrienoic acid	ALA	9(*R*),16(*S*)-DiHOTrE
9(*R*),16(*R*)-dihydroxy-octadecatrienoic acid	ALA	9(*R*),16(*R*)-DiHOTrE
threo-9,10-dihydroxy-octadecadienoic acid	ALA	*threo*-9,10-DiHODE
erythro-9,10-dihydroxy-octadecadienoic acid	ALA	*erythro*-9,10-DiHODE
erythro-12,13-dihydroxy-octadecadienoic acid	ALA	*erythro*-12,13-DiHODE
threo-12,13-dihydroxy-octadecadienoic acid	ALA	*threo*-12,13-DiHODE
erythro-15,16-dihydroxy-octadecadienoic acid	ALA	*erythro*-15,16-DiHODE
threo-15,16-dihydroxy-octadecadienoic acid	ALA	*threo*-15,16-DiHODE
10,13-dihydroxy-15(*Z*)-octadecenoic acid	ALA	15(*Z*)-10,13-DiHOME
9(*S*),10(*S*),13(*S*)-trihydroxy-octadecadienoic acid	ALA	9(*S*),10(*S*),13(*S*)-TriHODE
9(*S*),10(*S*),11(*R*)-trihydroxy-octadecadienoic acid	ALA	9(*S*),10(*S*),11(*R*)-TriHODE
9(*S*),12(*S*),13(*S*)-trihydroxy-octadecadienoic acid	ALA	9(*S*),12(*S*),13(*S*)-TriHODE
10-oxo-6(*Z*),12(*Z*)-octadecadienoic acid	GLA	6(Z),12(Z)-10-oxo-ODE
10-oxo-6(*Z*),11(*E*)-octadecadienoic acid	GLA	6(Z),11(E)-10-oxo-ODE

10-oxo-6(*Z*)-octadecenoic acid	GLA	6(Z)-10-oxo-OME
7-hydroxy-octadecatrienoic acid-γ	GLA	7-HOTrE-γ
9-hydroxy-octadecatrienoic acid-γ	GLA	9-HOTrE-γ
10-hydroxy-octadecatrienoic acid-γ	GLA	10-HOTrE-γ
12-hydroxy-octadecatrienoic acid-γ	GLA	12-HOTrE-γ
13-hydroxy-octadecatrienoic acid-γ	GLA	13-HOTrE-γ
18-hydroxy-octadecatrienoic acid-γ	GLA	18-HOTrE-γ
13-hydroxy-6(*Z*),9(*Z*)-octadecadienoic acid	GLA	13-HODE-γ
10-hydroxy-6(*Z*),12(*Z*)-octadecadienoic acid	GLA	6(*Z*),12(*Z*)-10-HODE
10-hydroxy-6(*Z*),11(*E*)-octadecadienoic acid	GLA	6(*Z*),11(*E*)-10-HODE
10-hydroxy-6(*Z*)-octadecenoic acid	GLA	6(*Z*)-10-HOME
9-hydroxy-octadecatetraenoic acid	SDA	9-HOTE
10-hydroxy-octadecatetraenoic acid	SDA	10-HOTE
12-hydroxy-octadecatetraenoic acid	SDA	12-HOTE
13-hydroxy-octadecatetraenoic acid	SDA	13-HOTE
15-hydroxy-octadecatetraenoic acid	SDA	15-HOTE
16-hydroxy-octadecatetraenoic acid	SDA	16-HOTE
18-hydroxy-octadecatetraenoic acid	SDA	18-HOTE
threo-6,7-dihydroxy-9(Z),12(Z),15(Z)-octadecatrienoic acid	SDA	*threo*-6,7-DiHOTrE
threo-9,10-dihydroxy-6(Z),12(Z),15(Z)-octadecatrienoic acid	SDA	*threo*-9,10-DiHOTrE
threo-12,13-dihydroxy-6(Z),9(Z),15(Z)-octadecatrienoic acid	SDA	*threo*-12,13-DiHOTrE
threo-15,16-dihydroxy-6(Z),9(Z),12(Z)-octadecatrienoic acid	SDA	*threo*-15,16-DiHOTrE
9-hydroxy-octadecapentaenoic acid	ODPA	9-HOPE
10-hydroxy-octadecapentaenoic acid	ODPA	10-HOPE
12-hydroxy-octadecapentaenoic acid	ODPA	12-HOPE
13-hydroxy-octadecapentaenoic acid	ODPA	13-HOPE
15-hydroxy-octadecapentaenoic acid	ODPA	15-HOPE

Eicosanoids		
**Compound**	**Parent PUFA**	**Abbreviation**
9α,11α,15S-trihydroxy-(8β)-prosta-5Z,13E,17Z-trien-1-oic acid	EPA	8-iso Prostaglandin F3α
9α,11α,15S-trihydroxy-(8β)-prosta-5Z,13E-dien-1-oic acid	ARA	8-iso Prostaglandin F2α
9α,11α,15S-trihydroxy-prosta-5Z,13E-dien-1-oic acid	ARA	prostaglandin F2α; dinoprost
9α,11β,15S-trihydroxy-prosta-5Z,13E-dien-1-oic acid	ARA	9α,11β-prostaglandin F2α
6-oxo-9α,11α,15S-trihydroxy-prost-13E-en-1-oic acid	ARA	6-keto-prostaglandin F1α
9α,11,15S-trihydroxy-thromba-5Z,13E,17Z-trien-1-oic acid	EPA	thromboxane B3
9,11,15-trihydroxy-thrombox-13-en-1-oic acid	DGLA	thromboxane B1
9α,11,15S-trihydroxythromba-5Z,13E-dien-1-oic acid	ARA	thromboxane B2
5S,12R,20-trihydroxy-6Z,8E,10E,14Z-eicosatetraenoic acid	ARA	20-hydroxy-leukotriene B4
5S,12R-dihydroxy-6Z,8E,10E,14Z-eicosatetraene-1,20-dioic acid	ARA	20-carboxy-leukotriene B4
5S,12R-dihydroxy-6E,8E,10E,14Z-eicosatetraenoic acid	ARA	6-trans-leukotriene B4
5S,12R-dihydroxy-6Z,8E,10E,14Z-eicosatetraenoic acid	ARA	leukotriene B4
(8S)-10-[(1R,2S,3S,5R)-3,5-dihydroxy-2-pentylcyclopentyl]-8-hydroxydeca-5,9-dienoic acid	ARA	iPF2α -IV
9α,15S-dihydroxy-11-oxo-thromba-5Z,13E,17Z-trien-1-oic acid	EPA	11-dehydro Thromboxane B3
9α,15S-dihydroxy-11-oxothromba-5Z,13E-dien-1-oic acid	ARA	11-dehydro Thromboxane B2
5,9α,11α-trihydroxy-(8β)-prosta-6E,14Z-dien-1-oic acid	ARA	5-iPF2α -VI
5S,6R,15S-trihydroxy-7E,9E,11Z,13E,17Z-eicosapentaenoic acid	EPA	5(S),6(R),15(S)-lipoxin A5
5S,14R,15S-trihydroxy-6E,8Z,10E,12E-eicosatetraenoic acid	ARA	5(S),14(R)-lipoxin B4
5S,6R,15S-trihydroxy-7E,9E,11Z,13E-eicosatetraenoic acid	ARA	5(S),6(R)-lipoxin A4
9-oxo-11α,15S-dihydroxy-prosta-5Z,13E,17Z-trien-1-oic acid	EPA	prostaglandin E3
9-oxo-11α,15S-dihydroxy-(8β)-prosta-5Z,13E-dien-1-oic acid	ARA	8-iso-prostaglandin E2
9α,15S-dihydroxy-11-oxo-prosta-5Z,13E,17Z-trien-1-oic acid	EPA	prostaglandin D3
9α,15S-dihydroxy-11-oxo-prosta-5Z,13E-dien-1-oic acid	ARA	prostaglandin D2
9α,15S-dihydroxy-11-oxo-prost-13E-en-1-oic acid	DGLA	prostaglandin D1
9-oxo-11α,15S-dihydroxy-prost-13E-en-1-oic acid	DGLA	prostaglandin E1
9,15-dioxo-11α-hydroxy-prost-5Z-en-1-oic acid	ARA	13,14-dihydro-15-keto-PGE2
5S,6R,15R-trihydroxy-7E,9E,11Z,13E-eicosatetraenoic acid	ARA	15(R)-Lipoxin A4
15S-hydroxy-14R-(S-cysteinyl)-5Z,8Z,10E,12E-eicosatetraenoic acid	ARA	14,15-leukotriene E4
11-oxo-15S-hydroxy-prosta-5Z,9,12E-trien-1-oic acid	ARA	delta-12-PGJ2

11-oxo-15S-hydroxy-prosta-5Z,9,13E-trien-1-oic acid	ARA	prostaglandin J2
9-oxo-15S-hydroxy-prosta-5Z,8(12),13E-trien-1-oic acid	ARA	prostaglandin B2
8,15-dihydroxy-5Z,9E,11Z,13E-eicosatetraenoic acid	ARA	8,15-DiHETrE
17,18-dihydroxy-5Z,8Z,11Z,14Z-eicosatetraenoic acid	EPA	17,18-DiHETE
5,15-dihydroxy-6E,8Z,10Z,13E-eicosatetraenoic acid	ARA	5,15-DiHETrE
(±)14,15-dihydroxy-5Z,8Z,11Z-eicosatrienoic acid	ARA	14,15-DHETrE
(±)14,15-dihydroxy-eicosa-5,8,11,17-tetraenoic acid	EPA	14,15-DiHETE
(±)11,12-dihydroxy-5Z,8Z,14Z-eicosatrienoic acid	ARA	11,12-DHETrE
12-hydroxy-5Z,8E,10E-heptadecatrienoic acid	ARA	12-HHTrE
(±)-15-hydroxy-11Z,13E-eicosadienoic acid	EDA	(±)15-HEDE
(±)-11-hydroxy-12E,14Z-eicosadienoic acid	EDA	(±)11-HEDE
(±)-18-hydroxy-5Z,8Z,11Z,14Z,16E-eicosapentaenoic acid	EPA	18-HEPE
(±)-15-hydroxy-5Z,8Z,11Z,13E,17Z-eicosapentaenoic acid	EPA	15-HEPE
(±)15-hydroxy-5Z,8Z,11Z,13E-eicosatetraenoic acid	ARA	15-HETE
15S-hydroxy-8Z,11Z,13E-eicosatrienoic acid	DGLA	15-HETrE
(±)8,9-dihydroxy-5Z,11Z,14Z-eicosatrienoic acid	ARA	8,9-DiHETrE
5,6-dihydroxy-8Z,11Z,14Z,17Z-eicosatetraenoic acid	EPA	5,6-DiHETE
(±)5,6-dihydroxy-8Z,11Z,14Z-eicosatrienoic acid	ARA	5,6-DiHETrE
11-oxo-prosta-5Z,9,12E,14E-tetraen-1-oic acid	ARA	15-deoxy-delta-12,14-PGJ2
±)19-hydroxy-5Z,8Z,11Z,14Z-eicosatetraenoic acid	ARA	19-HETE
(±)20-hydroxy-5Z,8Z,11Z,14Z-eicosatetraenoic acid	ARA	20-HETE
(±)-11-hydroxy-5Z,8Z,12E,14Z,17Z-eicosapentaenoic acid	EPA	11-HEPE
(±)-12-hydroxy-5Z,8Z,10E,14Z,17Z-eicosapentaenoic acid	EPA	12-HEPE
(±)-8-hydroxy-5Z,9E,11Z,14Z,17Z-eicosapentaenoic acid	EPA	8-HEPE
(±)-9-hydroxy-5Z,7E,11Z,14Z,17Z-eicosapentaenoic acid	EPA	9-HEPE
(±)11-hydroxy-5Z,8Z,12E,14Z-eicosatetraenoic acid	ARA	11-HETE
(±)12-hydroxy-5Z,8Z,10E,14Z-eicosatetraenoic acid	ARA	12-HETE
(±)8-hydroxy-5Z,9E,11Z,14Z-eicosatetraenoic acid	ARA	8-HETE
(±)-9-hydroxy-5Z,7E,11Z,14Z-eicosatetraenoic acid	ARA	9-HETE
(±)-5-hydroxy-6E,8Z,11Z,14Z,17Z-eicosapentaenoic acid	EPA	5-HEPE
(±)5-hydroxy-6E,8Z,11Z,14Z-eicosatetraenoic acid	ARA	5-HETE
8S-hydroxy-9E,11Z,14Z-eicosatrienoic acid	DGLA	8-HETrE
5S-hydroxy-6E,8Z,11Z-eicosatrienoic acid	MA	5-HETrE
(±)17(18)-epoxy-5Z,8Z,11Z,14Z-eicosatetraenoic acid	EPA	17,18-EpOTE
(±)14(15)-epoxy-5Z,8Z,11Z,17Z-eicosatetraenoic acid	EPA	14,15-EpOTE
(±)14(15)-epoxy-5Z,8Z,11Z-eicosatrienoic acid	ARA	14(15)-EpETrE
(±)11,12-dihydroxy-5Z,8Z,14Z-eicosatrienoic acid	ARA	11(12)-EpETrE
15-oxo-5Z,8Z,11Z,13E-eicosatetraenoic acid	ARA	15-oxoETE
12-oxo-5Z,8Z,10E,14Z-eicosatetraenoic acid	ARA	12-oxoETE
5-oxo-6E,8Z,11Z,14Z-eicosatetraenoic acid	ARA	5-oxoETE
(±)8(9)-epoxy-5Z,11Z,14Z-eicosatrienoic acid	ARA	8(9)-EETrE
(±)5(6)-epoxy-8Z,11Z,14Z-eicosatrienoic acid	ARA	5(6)- EETrE
18-hydroxy-5Z,8Z,11Z,14Z,16E-eicosapentaenoic acid	EPA	18-HEPE
15-hydroxy-5Z,8Z,11Z,13E-eicosatetraenoic acid	AA	15-HETE

11-hydroxy-5Z,8Z,12E,14Z-eicosatetraenoic acid	AA	11-HETE
12-hydroxy-5Z,8Z,10E,14Z-eicosatetraenoic acid	AA	12-HETE
5-hydroxy-6E,8Z,11Z,14Z-eicosatetraenoic acid	AA	5-HETE
15S-hydroxy-14R-(S-glutathionyl)-5Z,8Z,10E,12E-eicosatetraenoic acid	AA	14,15-leukotriene C4
5S-hydroxy-6R-(S-glutathionyl)-7E,9E,11Z,14Z-eicosatetraenoic acid	AA	leukotriene C4
5S-hydroxy-6R-(S-cysteinylglycinyl)-7E,9E,11Z,14Z-eicosatetraenoic acid	AA	leukotriene D4

Docosanoids		
**Compound**	**Parent PUFA**	**Abbreviation**
7,17-dihydroxy-8E,10Z,13Z,15E,19Z-docosapentaenoic acid	DPA	7,17-HDPA
7S,16R,17S-trihydroxy-4Z,8E,10Z,12E,14E,19Z-docosahexaenoic acid	DHA	resolvin D2
7S,8R,17S-trihydroxy-4Z,9E,11E,13Z,15E,19Z-docosahexaenoic acid	DHA	resolvin D1
7S,8R,17R-trihydroxy-4Z,9E,11E,13Z,15E,19Z-docosahexaenoic acid	DHA	17-epi-resolvin D1
10(S),17(S)-dihydroxy-4Z,7Z,11E,13Z,15E,19Z-docosahexaenoic acid	DHA	10,17-DiHDoHE
7S,14S-dihydroxy-4Z,8E,10E,12Z,16Z,19Z-docosahexaenoic acid	DHA	7-epi Maresin 1
(±)19,20-dihydroxy-4Z,7Z,10Z,13Z,16Z-docosapentaenoic acid	DHA	19,20-DiHDPA
(±)17-hydroxy-4Z,7Z,10Z,13Z,15E,19Z-docosahexaenoic acid	DHA	17-HDoHE
(±)14-hydroxy-4Z,7Z,10Z,12E,16Z,19Z-docosahexaenoic acid	DHA	14-HDoHE
(±)11-hydroxy-4Z,7Z,9E,13Z,16Z,19Z-docosahexaenoic acid	DHA	(±)11- HDoHEs
(±)8-hydroxy-4Z,6E,10Z,13Z,16Z,19Z-docosahexaenoic acid	DHA	(±)8- HDoHEs
(±)4-hydroxy-5E,7Z,10Z,13Z,16Z,19Z-docosahexaenoic acid	DHA	(±)4- HDoHEs
(±)16(17)-epoxy-4Z,7Z,10Z,13Z,19Z-docosapentaenoic acid	DHA	16(17)-EpDPE
(±)19(20)-epoxy-4Z,7Z,10Z,13Z,16Z-docosapentaenoic acid	DHA	19(20)-EpDPE
17-hydroxy-4Z,7Z,10Z,13Z,15E,19Z-docosahexaenoic acid	DHA	17-HDoHE
14-hydroxy-4Z,7Z,10Z,12E,16Z,19Z-docosahexaenoic acid	DHA	14-HDoHE

Table S6. Quantification of all lipids, pigments, and oxylipins presented as ng/g dry biomass across all datasets. (**A**) onset of thermal bleaching experiment and (**B**) additional dataset of cultured *B. minutum* under different nutrient concentrations.
See Excel file in electronic version.

Table S7. AOS-LOX classification of putative lipoxygenase (LOX) and allene oxide synthase-lipoxygenase (AOS-LOX) fusion protein candidates, based on multiple sequence alignment against the *Plexaura homomalla* AOS-LOX fusion protein (AAC47743.1; 1066 aa) as reference. Sequences were screened for the presence and identity of three diagnostic residues in the AOS domain (Thr66, His67, Tyr353), and three in the LOX domain (LH1, LH2, and C-terminal Ile/Val), following the molecular criteria established in Koljak et al. (1997), Oldham et al. (2004), and Mashhadi et al. (2016) [12,13,15]. Further description of table categories is presented below.
See Excel file in electronic version.

Table S8. Lipids and oxylipins that were significantly altered with symbiotic state and elevated temperature in Aiptasia evaluated by PERMANOVA and pairwise comparisons for each tested effect using Volcano-plots with F C> 2; $P < 0.05$ with FDR.
See Excel file in electronic version.

Table S9. Relative amounts (%) of *R* and *S* enantiomers and calculated **ee** values for octadecanoids in sample groups monitored in the SFC platform. Values suggesting enzymatic biosynthesis are highlighted.
See Excel file in electronic version.

Table S10. Lipids, pigments and oxylipins that were significantly altered with thermal stress in *Breviolum minutum* (Treatment *vs.* Control; t-test, $P < 0.05$ with FDR).
See Excel file in electronic version.

Table S11. Description of internal standards used for semi-quantification of different lipid classes described in the methods section. Asterisks indicate lipids that had external calibration curves described in **Table S14.**

**Standard MIX for Lipidomics**			
**IS MIX 1**			
**Internal standard**	**Quantified lipid classes**	**Work Concentration (ng µL $^{-1}$)**	**Source**
PC (17:0/17:0)	PC, oPC, DGCC, MGDG*, DGDG*	10	APL
PE (17:0/17:0)	PE, oPE	10	APL
Lyso PC-(17:0)	LysoPC, LysoDGCC, LoPC, Pigments	10	APL
FFA (17:0)	FFA	10	APL
**IS MIX 2**			
Ceramide (d18:1/17:0)	Cer, CAEP	10	APL
TAG (17:0/17:0/17:0)	TAG	10	APL

Abbreviation: APL: Avanti Polar Lipids.

Table S12. Lipid library in both negative and positive modes. * Indicates compounds that were characterised in positive mode but quantified in negative mode. *See Excel file in electronic version*.

Table S13. Reproducibility of quality controls (*e.g.,* pooled samples mix – N = 9 measured after every 30 samples) of lipidomics method in negative and positive modes. *See Excel file in electronic version*.

Table S14. Description of additional standard used for the quantification of glycolipids with external calibration curve.

Standard	Slope	R^2	Calibrated range (ng)
MGDG (18:3/18:3)	733.68	0.99	156-25000
PC (17:0/17:0)	1093.80	0.99	156-25000

External calibration curve was used to determine response factor using 5 different concentrations. Each point on dilution curve had half the concentration of the previous point and lower limits were based on MS inferior limit of detection. Correction factor was calculated by the ratio of the slope of each external standards against PC (17:0/17:0) IS. Standard was obtained from Avanti Polar Lipids. Response factor was calculated by the ratio of the slope of external MGDG (18:3/18:3) standard against PC (17:0/17:0) = 1.176.

Table S15. Linearity check of ionization response in lipidomics method (*e.g.,* pooled samples mix – N = 9 measured after every 30 samples) in negative and positive modes.

**Negative mode**	**Concentration range (ng µL^{-1})**	**Response range (x 10^4)**	**R^2**	**CV (%)**	**Observed RT (min)**
**UPPER WINDOW (550-1150 *m/z*)**					
**STD_Cer(d18:1/17:0)**	1.562-100	0.15-10.76	0.9954	7.02	14.55±0.01
**STD_PC (17:0/17:0)**	1.562-25*	0.18-2.78	0.9950	6.30	14.78±0.15
**LOWER WINDOW (150-600 *m/z*)**					
**STD_FFA (17:0)**	1.562-50*	0.15-4.17	0.9907	12.63	9.69±0.15
**STD_LysoPC (17:0)**	1.562-50*	0.13-5.55	0.9976	14.03	6.26±0.12
**Positive mode**					
**(400-1000 *m/z*)**					
**STD_LysoPC (17:0)**	1.562-25	0.81-12.46	0.9816	5.22	6.26±0.14
**STD_Cer(d18:1/17:0)**	1.562-25	0.42-13.41	0.9993	20.06	14.55±0.01
**STD_PC (17:0/17:0)**	1.562-25	1.64-16.98	0.9660	9.83	14.78±0.15
**STD_TAG (17:0/17:0/17:0)**	1.562-25	2.0-19.20	0.9958	21.35	19.09±0.03

Additional standard curves (N = 3) were measured after every 30 samples in each ionization mode as quality controls and for checking instrument linearity for controls. For PC (17:0/17:0), FFA (17:0) and lysoPC (17:0) in negative mode instrument linearity was not maintained until 100 ng µg^{-1}, so concentrations and calibrations were adjusted. RT: Retention times ±STD.

References

1. Matyash V, Liebisch G, Kurzchalia TV *et al.* Lipid extraction by methyl-tert-butyl ether for high-throughput lipidomics. *J Lipid Res* 2008;**49**(5):1137–46. https://doi.org/10.1194/jlr.D700041-JLR200.

2. Ermolenko EV, Sikorskaya TV. Lipidome of the reef-building coral Acropora cerealis: Changes under thermal stress. *Biochem Syst Ecol* 2021;**97**:104276. https://doi.org/10.1016/j.bse.2021.104276.

3. Imbs AB, Ermolenko EV, Grigorchuk VP *et al.* Current Progress in Lipidomics of Marine Invertebrates. *Mar Drugs* 2021;**19**(12):660. https://doi.org/10.3390/md19120660.

4. Sikorskaya TV. Coral Lipidome: Molecular Species of Phospholipids, Glycolipids, Betaine Lipids, and Sphingophosphonolipids. *Mar Drugs* 2023;**21**(6):335. https://doi.org/10.3390/md21060335.

5. Sikorskaya TV, Efimova KV, Imbs AB. Lipidomes of phylogenetically different symbiotic dinoflagellates of corals. *Phytochemistry* 2021;**181**:112579. https://doi.org/10.1016/j.phytochem.2020.112579.

6. T. Botana M, Chaves-Filho AB, Inague A *et al.* Thermal plasticity of coral reef symbionts is linked to major alterations in their lipidome composition. *Limnol Oceanogr* 2022;**67**(7):1456–69.

7. Kolmert J, Fauland A, Fuchs D *et al.* Lipid Mediator Quantification in Isolated Human and Guinea Pig Airways: An Expanded Approach for Respiratory Research. *Anal Chem* 2018;**90**(17):10239–48. https://doi.org/10.1021/acs.analchem.8b01651.

8. Botana MT, Lewis RE, Quaranta A *et al.* Octadecanoids as emerging lipid mediators in cnidarian-dinoflagellate symbiosis. *Commun Biol* 2025;**8**(1):1519. https://doi.org/10.1038/s42003-025-09104-6.

9. Liao Y, Smyth GK, Shi W. featureCounts: an efficient general purpose program for assigning sequence reads to genomic features. *Bioinformatics* 2014;**30**(7):923–30. https://doi.org/10.1093/bioinformatics/btt656.

10. Love MI, Huber W, Anders S. Moderated estimation of fold change and dispersion for RNA-seq data with DESeq2. *Genome Biol* 2014;**15**(12):550. https://doi.org/10.1186/s13059-014-0550-8.

11. Zámocký M, Hofbauer S, Schaffner I *et al.* Independent evolution of four heme peroxidase superfamilies. *Arch Biochem Biophys* 2015;**574**:108–19. https://doi.org/10.1016/j.abb.2014.12.025.

12. Oldham ML, Brash AR, Newcomer ME. Insights from the X-ray Crystal Structure of Coral 8R-Lipoxygenase. *J Biol Chem* 2005;**280**(47):39545–52. https://doi.org/10.1074/jbc.M506675200.

13. Koljak R, Boutaud O, Shieh BH *et al.* Identification of a Naturally Occurring Peroxidase-Lipoxygenase Fusion Protein. *Science* 1997;**277**(5334):1994–6. https://doi.org/10.1126/science.277.5334.1994.

14. Oldham ML, Brash AR, Newcomer ME. The structure of coral allene oxide synthase reveals a catalase adapted for metabolism of a fatty acid hydroperoxide. *Proc Natl Acad Sci* 2005;**102**(2):297–302. https://doi.org/10.1073/pnas.0406352102.

15. Mashhadi Z, Newcomer ME, Brash AR. The Thr–His Connection on the Distal Heme of Catalase-Related Hemoproteins: A Hallmark of Reaction with Fatty Acid Hydroperoxides. *ChemBioChem* 2016;**17**(21):2000–6. https://doi.org/10.1002/cbic.201600345.
